# Microcystins with Modified Adda^5^-Residues from a Heterologous Microcystin Expression System

**DOI:** 10.1021/acsomega.4c03332

**Published:** 2024-06-12

**Authors:** Christopher O. Miles, Pearse McCarron, Krista Thomas, Bakir Al-Sinawi, Tianzhe Liu, Brett A. Neilan

**Affiliations:** † Biotoxin Metrology, National Research Council Canada, Halifax, Nova Scotia B3H 3Z1, Canada; ‡ Norwegian Veterinary Institute, Postboks 64, 1431 Ås, Norway; § Diagnostic Technology Pty. Ltd., Sydney 2085, NSW, Australia; ∥ Department of Chemistry and Food Chemistry, Technical University of Dresden, 01069 Dresden, Germany; ⊥ School of Environmental and Life Sciences, The University of Newcastle, Callaghan 2308, NSW, Australia; # ARC Centre of Excellence in Synthetic Biology, Sydney, NSW 2019, Australia

## Abstract

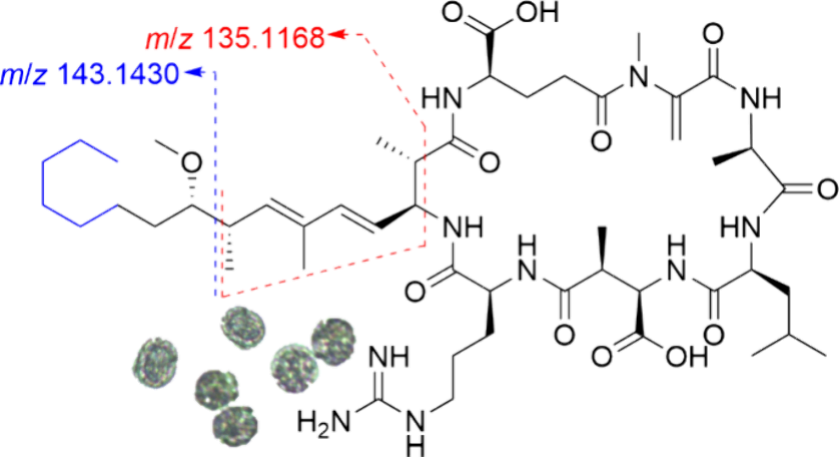

Microcystins are hepatotoxic cyclic heptapeptides produced by some cyanobacterial species and usually contain the unusual β-amino acid 3*S*-amino-9*S*-methoxy-2*S*,6,8*S*-trimethyl-10-phenyl-4*E*,6*E*-decadienoic acid (Adda) at position-5. The full microcystin gene cluster from *Microcystis aeruginosa* PCC 7806 has been expressed in *Escherichia coli*. In an earlier study, the engineered strain was shown to produce MC-LR and [d-Asp^3^]MC-LR, the main microcystins reported in cultures of *M. aeruginosa* PCC 7806. However, analysis of the engineered strain of *E. coli* using semitargeted liquid chromatography with high-resolution tandem mass spectrometry (LC–HRMS/MS) and thiol derivatization revealed the presence of 15 additional microcystin analogues, including four linear peptide variants and, in total, 12 variants with modifications to the Adda moiety. Four of the Adda-variants lacked the phenyl group at the Adda-terminus, a modification that has not previously been reported in cyanobacteria. Their HRMS/MS spectra contained the product-ion from Adda at *m*/*z* 135.1168, but the commonly observed product-ion at *m*/*z* 135.0804 from Adda-containing microcystins was almost completely absent. In contrast, three of the variants were missing a methyl group between C-2 and C-8 of the Adda moiety, and their LC–HRMS/MS spectra displayed the product-ion from Adda at *m*/*z* 135.0804. However, instead of the product-ion at *m*/*z* 135.1168, these three variants gave product-ions at *m*/*z* 121.1011. These observations, together with spectra from microcystin standards using in-source fragmentation, showed that the product-ion at *m*/*z* 135.1168 found in the HRMS/MS spectra of most microcystins originated from the C-2 to C-8 region of the Adda moiety. Identification of the fragmentation pathways for the Adda side chain will facilitate the detection of microcystins containing modifications in their Adda moieties that could otherwise easily be overlooked with standard LC–MS screening methods. Microcystin variants containing Abu at position-1 were also prominent components of the microcystin profile of the engineered bacterium. Microcystin variants with Abu^1^ or without the phenyl group on the Adda side chain were not detected in the original host cyanobacterium. This suggests not only that the microcystin synthase complex may be affected by substrate availability within its host organism but also that it possesses an unexpected degree of biosynthetic flexibility.

## Introduction

Cyanobacteria are a rich source of secondary metabolites^1^ that include toxic compounds with significant implications for human health.^2^ Microcystins are a group of cyclic heptapeptide hepatotoxins produced by several genera of cyanobacteria, including *Microcystis*, *Dolichospermum*, *Oscillatoria*, and *Nostoc*,^3−8^ and are the cyanotoxins most commonly associated with cyanobacterial blooms of freshwater bodies.^9^ Microcystins are inhibitors of protein phosphatase type 1 and 2A, binding to the catalytic site, and may subsequently become covalently bonded to the enzyme via reaction with the thiol of a cysteinyl residue that lies close to the catalytic center.^10,11^

**Figure 1:**
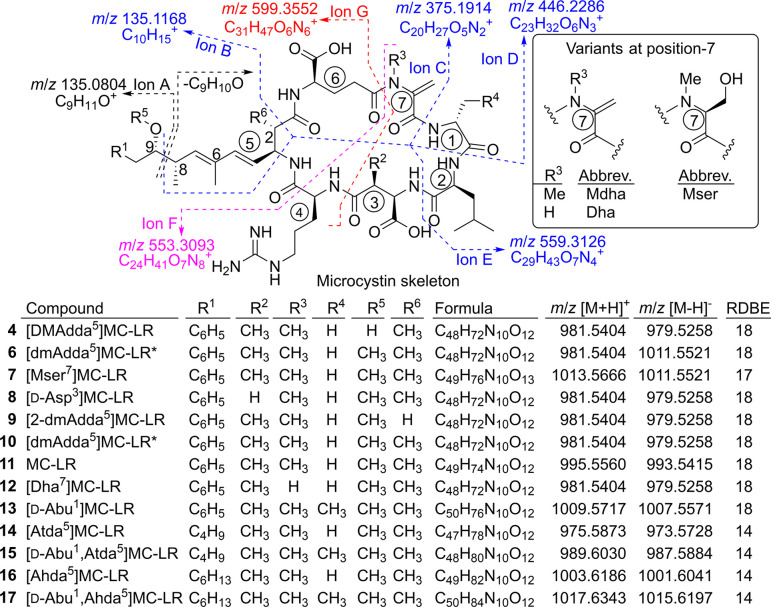
Structures, formulas, and exact masses of ions of MC-LR (**11**) and related congeners detected by LC–HRMS in the heterologous expression system, eight of which (**4**, **6**, **9**, **10**, and **14**–**17**) contained modified Adda^5^-residues. Formulas are for the neutral molecules, and RDBE is the number of rings plus double bonds calculated from the formula. The product-ion *m*/*z* values and the depicted neutral loss of C_9_H_10_O are for MC-LR (**11**) (i.e., R^1^ = C_6_H_5_, R^2^, R^3^, R^5^, R^6^ = Me, R^4^ = H), and observed product-ions for **4** and **6**–**17** vary with substitution at R^1^–R^6^ and amino-acid-7 (Table 1). Numbers in circles denote the amino acid residue numbers, and atom numbers are shown for the Adda^5^ residue. Compounds marked with an asterisk (**6** and **10**) are also missing a methyl group at C-6 or C-8 of Adda^5^. The structures of **1**–**3**, **5**, and MC-LA are shown in Figure 7. Compounds **4**, **8**, **11**, and **12** were also detected in the extract of *M. aeruginosa* PCC 7806.

More than 250 microcystin analogues have been reported, including microcystin-LR (MC-LR (**11**)).^1,9^ The structure of **11** contains d-alanine^1^, d-β-methylaspartic acid^3^, the unusual β-amino acid Adda^5^ (3*S*-amino-9*S*-methoxy-2*S*,6,8*S*-trimethyl-10-phenyl-4*E*,6*E*-decadienoic acid) group that is uniquely associated with microcystins and the closely related nodularins, d-glutamic acid^6^, and Mdha^7^ (*N*-methyldehydroalanine). In microcystins, the variable elements at position-2 and -4 are l-amino acids, which in the case of **11** are l-leucine (l) and l-arginine (R), respectively (Figure 1). Although the variability in position-2 and -4, along with position-3 and -7, are responsible for the vast majority of microcystin variants, amino-acid substitutions have been reported for all positions.^9^ Detection of microcystins by liquid chromatography–mass spectrometry (LC–MS) often relies on detection of a characteristic fragment at *m*/*z* 135.0804 arising from the Adda^5^ moiety. However, the thiol-reactivity of the Mdha or dehydroalanine (Dha) residues often found at position-7 of microcystins, which are responsible for their covalent binding to the cysteinyl residue of protein phosphatases, can also be exploited to enhance the detectability of microcystins by LC–MS.^12,13^

Microcystins are biosynthesized by one polyketide synthase (PKS), three nonribosomal peptide synthases (NRPSs), and two hybrid NRPS/PKSs. The microcystin biosynthetic gene cluster in *Microcystis aeruginosa* PCC 7806 encodes ten open reading frames that are transcribed in two operons, spanning 55 kbp and centrally transcribed by a bidirectional promoter.^14^ The proposed biosynthesis of microcystins starts with the assembly of the Adda group.^15^ Phenylpropanoids are activated and the C-1 carbon is truncated to yield the desired phenylacetate starter unit by McyG.^16^ Malonyl-CoA units are then successively incorporated by McyG, McyD, and McyE.^15^ The Adda chain is modified by McyJ,^17^ and the remaining amino acids are then incorporated by McyE, McyA, McyB, and McyC,^18^ with the final cyclization step catalyzed by the thioesterase domain of McyC.^19^

The full microcystin biosynthetic gene cluster from *M. aeruginosa* PCC 7806 has been successfully expressed in *Escherichia coli*.^20^ Replacement of the native central bidirectional promoter with a tetracycline-inducible promoter drove efficient transcription of the two operons. The resultant engineered *E. coli* strain was reported to predominantly produce MC-LR (**11**) and its demethylated variant [d-Asp^3^]MC-LR (**8**).^20^ That study also demonstrated control of analogue production by the addition of β-methylaspartic acid to the fermentation medium, resulting in the almost exclusive production of **11** (96%) relative to **8**.^17^

Here we report a detailed examination of the products of this heterologously expressed microcystin synthase using semitargeted LC–MS^2^ and LC–HRMS/MS methods in combination with mercaptoethanol derivatization. This combination of analytical chemistry and functional-group-specific chemical reactivity led to the identification of an array of unexpected microcystin congeners, including several that contain unprecedented modifications to their Adda moieties.

## Results and Discussion

The olefinic methylene group present in the Mdha and Dha groups, present in more than 80% of the known microcystins,^9^ reacts rapidly and efficiently with a range of thiols under weakly basic conditions.^9,13^ This reactivity, in combination with LC–MS analysis, has proved to be a powerful tool for identifying known and novel microcystins in challenging sample matrices^12,13,21−24^ as well as for differentiating Mdha^7^-containing microcystins from their much less reactive isomers containing dehydrobutyrine at position-7 (Dhb^7^).^21,25,26^ Recently, the entire functional microcystin biosynthetic gene cluster from *M. aeruginosa* PCC 7806 was constructed and expressed in *E. coli*.^20^ We applied mercaptoethanol derivatization (Figures S1 and S2) together with semitargeted LC–HRMS/MS analysis to dissect the microcystin profile of an extract from this transformed *E. coli* culture, to verify the identity of the microcystin congeners present and to investigate whether unanticipated analogues might also be present. An extract of a culture of PCC 7806 was analyzed similarly for comparison.

**Figure 2:**
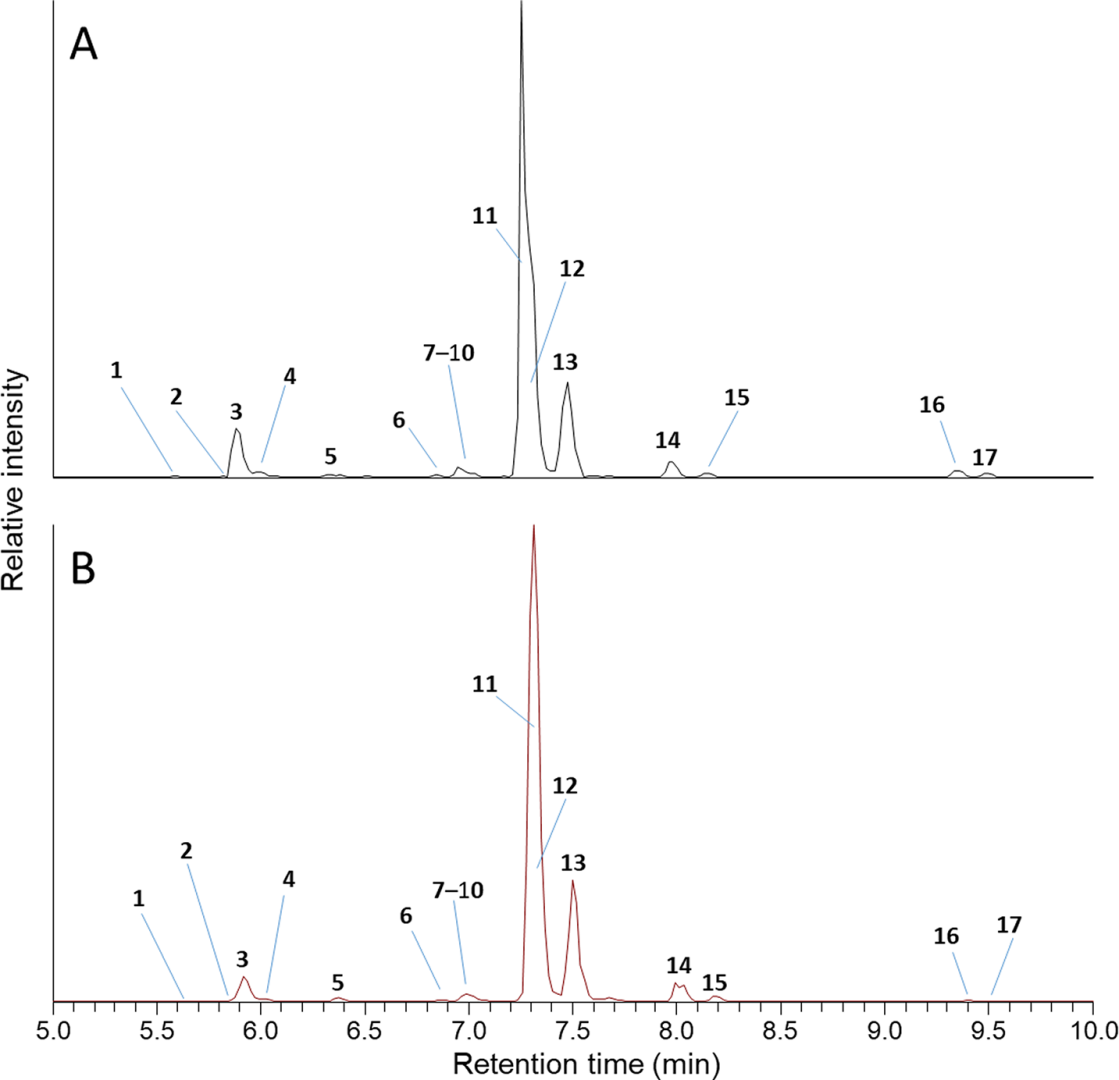
LC–HRMS (method B) full-scan chromatograms: (A) in positive ionization mode and (B) in negative ionization mode of the extract from the culture of the transformed *E. coli*, extracted (±5 ppm) for the exact masses of the [M + H]^+^ or [M – H]^−^ ions of **1**–**17** listed in Figures 1 and 7. Compound numbers, together with structures, elemental compositions, retention times, and relative abundances are shown in Figures 1 and 7 and in Table 1.

We also applied LC–MS^2^ using an ion trap mass spectrometer to supplement the analysis. Although LC–MS^2^ was at unit mass resolution and had a low-mass limit due to the inherent limitations of the ion trap, LC–MS^2^ spectra obtained in positive mode from this instrument were much richer in higher-mass fragments (*m*/*z* > 550) than those obtained with the LC–HRMS/MS instrument (Figures S3–S16). For example, the relative intensity of the ion resulting from neutral loss of the C_9_H_10_O unit at the terminus of the Adda side chain was often around an order of magnitude stronger in the LC–MS^2^ spectra than in the LC–HRMS/MS spectra of the same compound. Thus, the two sets of spectra complemented each other. Using this approach, we found more than 16 candidate peaks (Table 1, Figure 2) showing mass spectral fragmentations and thiol reactivities characteristic of microcystins in the transformed *E. coli* culture.

**Table 1:** Retention Times (*t*_R_ (min)), Accurate Masses and Their Mass Errors, Thiol-Reactivities, Relative Abundances, and Structurally-Informative Product-Ions (Positive Ionization Mode) Observed for Microcystin Congeners Identified by LC–HRMS/MS with Mercaptoethanol Derivatization in an Extract of *E. coli* Expressing the Microcystin Synthetase Gene Cluster^a^

		Accurate mass (*m*/*z*) and Δ_*m*_ (ppm)					Rel. Abund. (%)		Structurally informative product-ions (pos. mode)					
	*t*_R_	[M + H]^+^	Δ_*m*_	[M – H]^−^	Δ_*m*_	Thiol	[M + H]^+^	[M–H]^−^	Ion A	Ion B	Ion C	Ion D	Ion F	Ion G
**1**	5.63	771.4300	1.6	769.4156	1.9	Yes	0.23	0.12	135.08	135.11	375	446		
**2**	5.84	785.4455	1.5	783.4310	1.6	Yes	0.21	0.14	135.08	135.11	375	446		
**3**	5.92	714.4079	1.1	712.3935	1.1	Yes	7.77	3.80	135.08	135.11	375	446		
**4**	6.01	981.5415	1.1	979.5267	0.9	Yes	0.82	0.36	121.06^b^	135.11	375	446	553	585
**5**	6.37	728.4230	0.1	726.4095	1.6	Yes	0.89	0.67	135.08	135.11	375	460		
**6**	6.87	981.5426	2.3	979.5273	1.5	Yes	0.34	0.26	135.08	121.10	361	432	553	585
**7**	6.99	1013.5690	2.4	1011.5538	1.7	No	1.18	1.02	135.08	135.11	393	446^c^	571	599
**8**	7.02	981.5424	2.1	979.5272	1.4	Yes	0.60^d^	0.50	135.08	135.11	375	446	539	599
**9**	7.03					Yes			135.08	121.10	361	432	553	585
**10**	7.06					Yes			135.08	121.10	361	432	553	585
**11**	7.31	995.5546	0.4	993.5422	0.8	Yes	66.82	71.27	135.08	135.11	375	446	553	599
**12**	7.31	981.5410	0.6	979.5269	1.1	Yes	1.29	1.74	135.08	135.11	361	432	539	599
**13**	7.50	1009.5724	0.7	1007.5584	1.3	Yes	14.03	15.67	135.08	135.11	375	460	567	599
**14**	8.00	975.5892	1.9	973.5741	1.4	Yes	2.80	3.16	115.11	135.11	375	446	553	579
**15**	8.19	989.6054	2.5	987.5908	2.4	Yes	0.76	0.89	115.11	135.11	375	460	567	579
**16**	9.39	1003.6207	2.0	1001.6063	2.2	Yes	1.48	0.27	143.14	135.11	375	446	553	607
**17**	9.56	1017.6356	1.3	1015.6210	1.2	Yes	0.79	0.14	143.14	135.11	375	460	567	607

a Measured using LC–HRMS method B, thiol reactivity determined with *d*_0_/*d*_4_-mercaptoethanol. Masses of characteristic ions (see Figures 1 and 7) were measured to 4 decimal places but are displayed here with the fewest number of decimal places consistent with differentiating ions and were in all cases consistent (±5 ppm) with the elemental composition of proposed structures and product-ions. Additional data is presented in the Supporting Information file. For compound names, exact masses, and neutral formulas, see Figures 1 and 7.

b Weak peak.

c Includes neutral loss of H_2_O.

d Compounds **8**–**10** had similar retention times and accurate masses and could only be differentiated via their product-ion spectra, which suggested that **8**–**10** were present in a ratio of approximately 2:9:5.

In contrast, the extract of *M. aeruginosa* PCC 7806 contained a much simpler microcystin profile. MC-LR (**11**) and [d-Asp^3^]MC-LR (**8**) were the main components, accompanied by low levels of [DMAdda^5^]MC-LR (**4**) and [d-Asp^3^,DMAdda^5^]MC-LR, as reported previously.^17,20^ In addition, we detected the artifactual methyl esters of the main microcystins (**11** and **8**), [Glu(OMe)^6^]MC-LR and [d-Asp^3^,Glu(OMe)^6^]MC-LR, as well as trace levels of [Dha^7^]MC-LR (**12**). Two broad peaks that did not react with mercaptoethanol were tentatively identified as *seco*MC-LR and *seco*[d-Asp^3^]MC-LR (Figures S17–S20), based on the close parallels of their LC–HRMS/MS spectra with product-ion spectra reported previously^27^ for *seco*[d-Asp^3^]MC-RR. However, compounds **1**–**3**, **5**–**7**, **9**, **10**, and **13**–**17** that were detected in the transformed *E. coli* extract were not detected in the *M. aeruginosa* PCC 7806 extract.

### Identification of Microcystins

**Figure 3:**
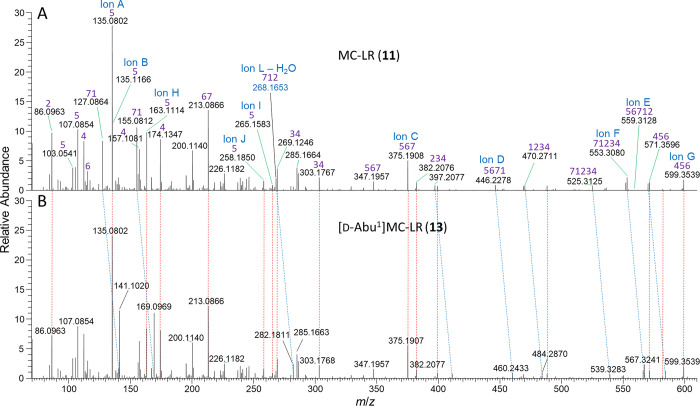
LC–HRMS/MS spectra of (A) MC-LR (**11**) and (B) [d-Abu^1^]MC-LR (**13**) in the extract of the transformed *E. coli* culture, in positive ionization mode from *m*/*z* 70–610. Spectra are scaled relative to the intensity of their residual precursor ions and in this region are essentially identical (selected peaks joined by red dashed lines) apart from the peaks attributable to the product-ions containing the amino acid at position-1 (selected peaks joined by blue dashed lines), which were all heavier by 14.0157 Da (consistent with CH_2_) in **13** than in **11**. Numbers in purple for MC-LR show the amino acid residue-numbers (Figure 1) from which the product-ions originate (Table S1), and cleavages for ions A–L are shown in Figures 1 and 7.

MC-LR (**11**) was the predominant microcystin produced by the engineered *E. coli* (Table 1), as previously reported,^17^ accounting for approximately two-thirds of the total microcystins detected (Table 1). Additionally, LC–MS^2^ and LC–HRMS/MS analysis of their [M + H]^+^ and [M – H]^−^ ions together with mercaptoethanol derivatization revealed a total of 16 other microcystin variants (Table 1), of which [d-Asp^3^]MC-LR (**8**) was only a very minor component. The second most abundant microcystin (**13**), eluted after **11**, reacted with one molecule of mercaptoethanol and, in full-scan LC–HRMS, displayed accurate masses consistent with those of MC-HilR (Table 1). However, examination of its positive ionization HRMS/MS spectrum showed that although the fragmentation pattern of **13** closely paralleled that of **11**, all product-ions attributable to fragments containing the amino acid from position-1 of **13** were heavier by 14.0157 Da (corresponding to CH_2_) than the equivalent product-ions from **11** (Figures 3, S6, S8, S21, and S22, and Tables 1 and S1). For example, the positive mode HRMS/MS spectrum of **13** contained prominent product-ions at *m*/*z* 375.1908 (C_20_H_27_N_2_O_5_^+^, Δ –1.7 ppm; from Adda^5^–d-Glu^6^–Mdha^7^ – C_9_H_10_O, ion C), 460.2433 (C_24_H_34_N_3_O_6_^+^, Δ –2.0 ppm; from Adda^5^–d-Glu^6^–Mdha^7^–d-Abu^1^ – C_9_H_10_O, ion D), and 141.1020 and 169.0969 (C_7_H_13_N_2_O^+^ and C_8_H_13_N_2_O_2_^+^, Δ –1.3 and −1.7 ppm; both from Mdha^7^–d-Abu^1^), whereas the corresponding peaks for **11** were at *m*/*z* 375.1904, 446.2274, 127.0865, and 155.0814, respectively. This is consistent with the presence of d-aminobutyric acid at position-1 of **13** (instead of d-alanine, as in **11**). Compound **13** was thus identified as [d-Abu^1^]MC-LR, and its HRMS/MS spectrum and retention time were essentially identical to those reported for the same compound as part of a study of dietary supplements.^12^

Four other later-eluting compounds (**14**–**17**) also reacted with one equivalent of mercaptoethanol and had *m*/*z* values for their [M + H]^+^ and [M – H]^−^ ions that indicated that they each contained 12 oxygen and 10 nitrogen atoms (Table 1, and Figures S1 and S2), suggesting that they might be analogues of the much more abundant **11**. However, no compounds with these elemental compositions appear on recent database listings of known microcystins,^1,9,28^ prompting a detailed examination of their HRMS/MS spectra to determine whether they were MCs and, if so, to attempt to determine their structures.

**Figure 4:**
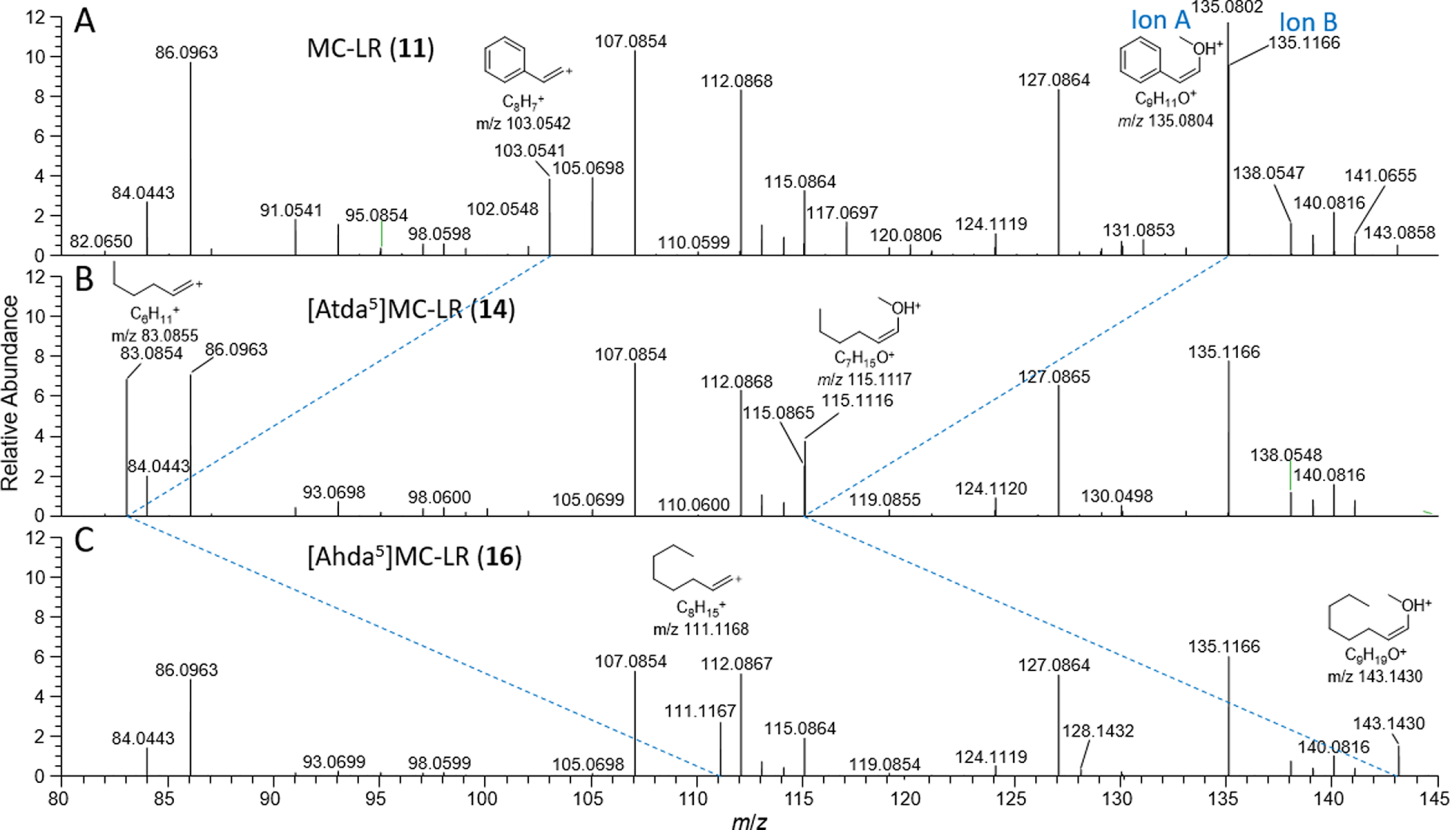
LC–HRMS/MS spectra (method B) of (A) MC-LR (**11**), (B) Adda-modified analogues **14**, and (C) **16**, in positive ionization mode from *m*/*z* 80–145. Spectra are scaled relative to the intensity of their residual precursor ions and in this region are essentially identical apart from the peaks corresponding to the product-ions from the phenyl terminus of the Adda moiety of MC-LR (**11**) at *m*/*z* 135.0804 (which is off-scale, see Figure 5) and 103.0542. The mass differences for the marked (blue dashed lines) product-ions differ by the differences in the exact masses of the intact precursor ions, suggesting that the difference between MC-LR (**11**) and novel congeners **14** and **16** (−19.9685 and +8.0629 Da, respectively) resides in their Adda-termini.

**Figure 5:**
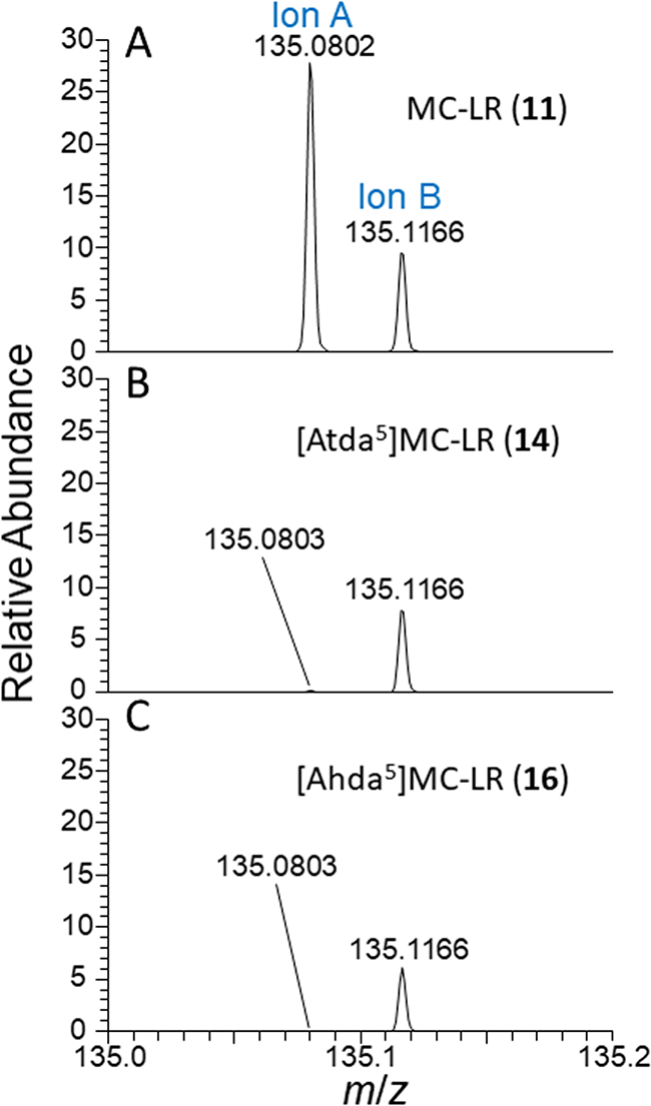
LC–HRMS/MS spectra of MC-LR (**11**) and its Adda-modified analogues **14** and **16** in positive ionization mode, showing an expansion (from Figure 4) of the region from *m*/*z* 135.0–135.2. Spectra are scaled relative to the intensity of their residual precursor ions and show the presence or the near-absence of the *m*/*z* 135.0804 product-ion in the congeners with modified Adda, while the product-ion at *m*/*z* 135.1168 retained a similar relative intensity for all three congeners.

**Figure 6:**
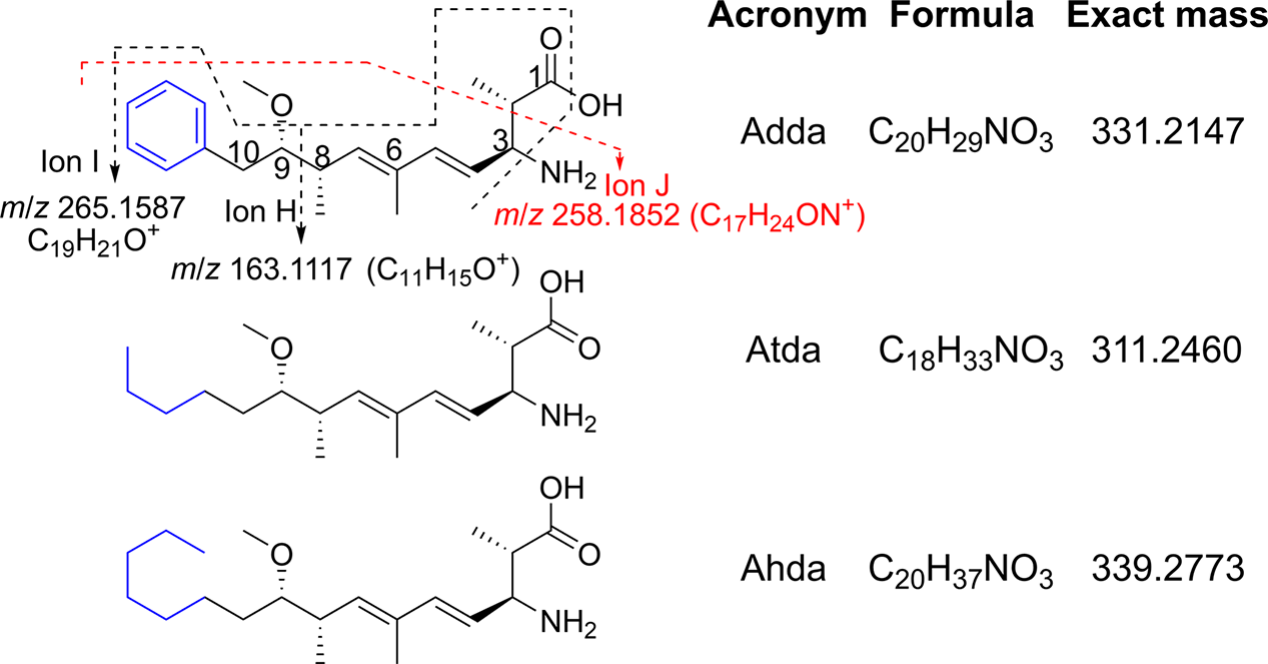
Structures of the Adda^5^ and modified-Adda^5^ side-chains present in microcystins identified in this study, with the variable Adda-terminus marked in blue. Characteristic mass spectral cleavages involving the Adda^5^-moiety of intact microcystins (**7**, **8**, and **11**–**13**) are shown and are additional to those shown in Figure 1. The corresponding product-ions were also observed for MCs containing DMAdda^5^ (**4**), 6-/8-dmAdda^5^ (**6** and **10**), 2-dmAdda^5^ (**9**), Atda^5^ (**14** and **15**), and Ahda^5^ (**16** and **17**). Table S2 presents a tabulation of the expected *m*/*z* values of useful product-ions involving cleavages of microcystins containing modified Adda moieties.

The most abundant of these (**14**) displayed *m*/*z* values for its [M + H]^+^ and [M – H]^−^ ions consistent with an elemental composition of C_47_H_78_N_10_O_12_, which is 2 carbon atoms and 4 ring-plus-double-bond equivalents (RDBE) less, and 4 hydrogen atoms more, than for **11** (overall exact mass difference of −19.9687 Da). Comparison of the positive ion MS^2^ and HRMS/MS spectra of **14** with those of **11** (Figures 4, S6, S9, S13, S14, S23–S26, and Tables S1 and S2) revealed many similarities and some surprising differences. Most unexpected among these was the almost total absence of a product-ion at *m*/*z* 135.0804 (ion A, Figure 1), even though the product-ion at *m*/*z* 135.1168 (ion B, Figure 1) was present (Figure 5). Comparison of the HRMS/MS spectra of **11** and **14** (Figures 4 and S23–S26) revealed that *m*/*z* for product-ions containing intact Adda^5^ units in **11** occurred at about 19.9687 lower *m*/*z* in **14** (e.g., ion G), whereas product-ions attributable to fragments not containing Adda^5^ had identical *m*/*z* values for **11** and **14** (Table S1). Furthermore, all product-ions involving the neutral loss of C_9_H_10_O (134.0732 Da) from **11** (the same cleavage that also gives rise to ion A) occurred at the same *m*/*z* values in both **11** and **14**. For example, both **11** and **14** had product-ions with *m*/*z* 375.1915 (C_20_H_27_N_2_O_5_^+^; from Adda^5^–d-Glu^6^–Mdha^7^ – C_9_H_10_O (in the case of **11**, ion C) and 861.4829 (C_40_H_65_N_10_O_11_^+^; [*cyclo*-[d-Ala^1^–Leu^2^–d-Masp^3^–Arg^4^–Adda^5^–d-Glu^6^–Mdha^7^] – C_9_H_10_O]^+^ in the case of **11**). This indicated that the difference between **11** and **14** lay in the terminal part of the Adda^5^ side-chain, beyond C-8. Consistent with this, a product-ion corresponding to ion A was observed at *m*/*z* 115.1116 (C_7_H_15_O^+^, Δ –1.1 ppm) in the HRMS/MS spectrum of **14** (Figure 4). The only way this can occur is if the terminus of the side chain of **14** contains an aliphatic 4-carbon (e.g., butyl) moiety instead of the phenyl group at C-10 of **11**. Thus, **14** contains 3*S*-amino-9*S*-methoxy-2*S*,6,8*S*-trimethyl-4*E*,6*E*-tetradecadienoic acid (Atda, Figure 6) rather than Adda at position-5, although the possibility of branching in the butyl terminus of the Atda group cannot be excluded based on HRMS/MS data alone.

Consequently, the product-ion at *m*/*z* 135.1168 (C_10_H_15_^+^) cannot originate from the terminus of the Adda moiety, as has been suggested,^29^ since this ion was present in the spectrum of **14**. This was confirmed by LC–HRMS/MS of the *m*/*z* 861.4829 ion produced by in-source neutral loss of 134.0732 Da (C_9_H_10_O) from the Adda^5^-terminus of [M + H]^+^ of **11**. The product-ion at *m*/*z* 135.1168 was a major fragment in this spectrum (Figures S27–S29), and, given the elemental composition of this ion, it must originate from the remainder of the Adda side chain, from C-2 to C-8 (ion B, Figure 1 and Table S2). This fragment is therefore related (via neutral loss of CO) to the *m*/*z* 163.1117 (C_11_H_15_O^+^, ion H) proposed by Liu et al. in the product-ion spectrum of [d-Asp^3^,DMAdda^5^]MC-LR.^17^ We obtained similar results from LC–HRMS/MS of [M + H – C_9_H_10_O]^+^ ions obtained after in-source fragmentation of standards of MC-LA and MC-RR (Figure S30), showing that these findings are general to microcystins containing an Adda^5^ residue, regardless of their arginine content. A weak peak was nonetheless present at *m*/*z* 135.0804 (C_9_H_11_O^+^) in all of the in-source fragmentation experiments, even though the C-9–C-16 terminus was eliminated by neutral loss prior to the ions reaching the collision cell, suggesting that a small proportion of the *m*/*z* 135.0804 may be produced from the residual portion of the Adda side-chain.

Although the product-ion at *m*/*z* 135.0804 was very minor in **14**, new low-mass fragments were present at *m*/*z* 115.1117 (ion A of **14**) and 83.0855, 19.9687 Da less than the product-ions present at *m*/*z* 135.0804 (ion A of **11**) and 103.0542 in the HRMS/MS spectra of **11** (but not of the in-source-fragmented **11**). Apart from this, the *m*/*z* 80–145 region of the HRMS/MS spectra of **11** and **14** were nearly identical, showing that the ions at *m*/*z* 83.0855 and 115.1117 (Atda) and at 103.0542 and 135.0804 (Adda) derive from cleavage of Atda^5^ and Adda^5^ between C-8 and C-9, as shown in Figure 4. Furthermore, ions H, I, and J (Figure 6) of **14** appeared at *m*/*z* 163.1116 (C_11_H_15_O^+^, Δ –0.9 ppm), 245.1896 (C_17_H_25_O^+^, Δ –1.6 ppm), and 238.2164 (C_15_H_28_ON^+^, Δ –0.8 ppm) compared to *m*/*z* 163.1117 (C_11_H_15_O^+^), 265.1587 (C_19_H_21_O^+^), and 258.1852 (C_17_H_24_ON^+^) in **11** (Figures S25 and S26, and Table S2), confirming that the site of modification in analogue **14** was the Adda-terminus.

Another minor later-eluting peak (**16**) had [M + H]^+^ and [M – H]^−^ ions consistent with an elemental composition of C_49_H_82_N_10_O_12_, which is 8 hydrogen atoms more and 4 RDBEs less than for **11** (with an overall exact mass difference of +8.0629 Da), suggesting addition of C_2_H_4_ relative to **14**. The positive ion MS^2^ and HRMS/MS spectra of **16** (Figures 1, 4, 5, S11, S13, S14, and S23–S26, and Table S2) contained a product-ion at *m*/*z* 861.4817 (C_40_H_65_N_10_O_11_^+^, Δ –1.4 ppm) due to neutral loss of the terminal part of the Adda-like side chain, as well as product-ions B–D, F, and H at *m*/*z* 135.1166 (Δ –1.5 ppm), 375.1908 (Δ –1.5 ppm), 446.2286 (Δ –1.3 ppm), 553.3091 (C_24_H_41_O_7_N_8_^+^, Δ –0.3 ppm), and 163.1115 (Δ –1.5 ppm), the same as in **11** and **14**. However, product-ions A, G, and I of **16** were observed at *m*/*z* 143.1431 (C_9_H_19_O^+^, Δ 0.4 ppm), 607.4175 (C_31_H_55_N_6_O_6_^+^, Δ –0.4 ppm), and 273.2210 (C_19_H_29_O^+^, Δ –1.0 ppm), respectively, rather than at *m*/*z* 135.0804 (C_9_H_11_O^+^), 599.3552 (C_31_H_47_N_6_O_6_^+^), and 265.1587 (C_19_H_21_O^+^) for **11** (Figures 4, 5, and S23–S26). These results establish **16** as a variant of **11** in which the phenyl group at C-10 has been replaced with a hexyl group. Compound **16** therefore contains 3*S*-amino-9*S*-methoxy-2*S*,6,8*S*-trimethyl-4*E*,6*E*-hexadecadienoic acid (Ahda, Figure 6) rather than Adda at position-5, although branching in the hexyl portion cannot be determined by MS/MS analysis.

**Figure 7:**
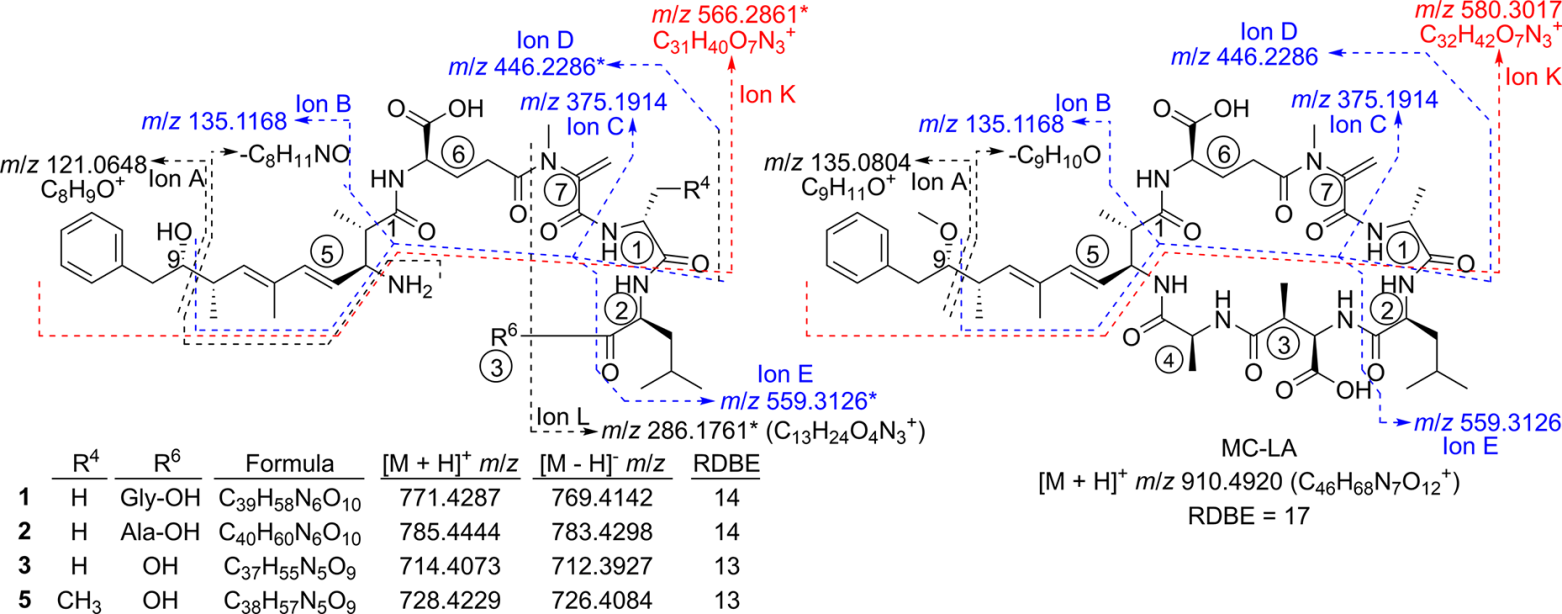
Proposed structures of analogues **1**–**3** and **5** (left) and of MC-LA (right), showing some of their characteristic fragmentation pathways (positive mode product-ion *m*/*z* values on the left-hand structure are for **3**). Note that ions marked with asterisks (ions D, E, K, and L) are increased by 14.0157 Da in **5** due to R^4^, while *m*/*z* for ion K also varies between MC-LA and **1**–**3** and **5** due to the substituents at C-9 of the Adda^5^ moiety. Ion K was also present in the Arg^4^-containing microcystins in Figure 1, but it was much less prominent. Elemental compositions for ions A–E are shown in Figure 1.

LC–HRMS with mercaptoethanol derivatization and LC–HRMS/MS with full-scan/data-independent analysis (FS/DIA) suggested the presence of four early eluting candidate microcystin-like compounds (**1**–**3** and **5**) with elemental compositions containing 5 or 6 nitrogen atoms, 9 or 10 oxygen atoms, and 13 or 14 RDBE (Figures 2, 7, and S1, and Table 1). Because the core structure of intact microcystins contains a minimum of seven nitrogen and 12 oxygen atoms and 17 RDBE (Figures 1 and 7), these compounds cannot be fully formed microcystins. Nevertheless, their HRMS/MS spectra showed many product-ions indicative of microcystins, including ions B–D, with virtually the same accurate masses as for **11** and for MC-LA (except for **5**, for which ion D was consistent with a d-Abu^1^-containing analogue) (Figures 1, 7, and S31–S34). No obvious product-ions attributable to ion A were observed, nor were there any ions attributable to the conventional neutral loss of C_9_H_10_O or C_8_H_8_O from an Adda or DMAdda side chain. However, prominent neutral losses of 137.0841 Da, corresponding to C_8_H_11_NO, were present in the HRMS/MS spectra of all four compounds (Figure S32). This is consistent with loss of C_8_H_8_O from a DMAdda^5^ side-chain together with loss of NH_3_, and it is indicative of the presence of an open-ring microcystin analogue in which DMAdda–NH_2_ is linked via its carboxylic acid to other amino acids. Analogous prominent neutral losses of C_9_H_10_O + NH_3_ have been reported in MS/MS spectra for a range of open-ring microcystin analogues containing Adda–NH_2_ at the amino terminus of a peptide chain.^30−32^ Many of the product-ions of **1**–**3** were identical to those of MC-LA, including ions at 559.3126 (C_29_H_43_N_4_O_7_^+^, with Δ –1.1 to +0.3 ppm; ion E) from DMAdda^5^–d-Glu^6^–Mdha^7^–d-Ala^1^–Leu^2^ – C_8_H_8_O – NH_3_ (Figures 7 and S32), with a corresponding product-ion at *m*/*z* 573.3281 (C_30_H_45_N_4_O_7_^+^, Δ –0.4 ppm) in the spectrum of **5** indicating the presence of d-Abu^1^. The equivalent product-ions from DMAdda^5^/Adda^5^–d-Glu^6^–Mdha^7^–d-Ala^1^/d-Abu^1^–Leu^2^ – NH_3_ (i.e., without neutral loss of C_8_H_8_O) were also present at *m*/*z* 679.3701 and 693.3858 in **1**–**3**, **5**, and MC-LA (Figure S32). Thus, **3** is H_2_N-DMAdda^5^–d-Glu^6^–Mdha^7^–d-Ala^1^–Leu^2^-OH, and **5** is the corresponding d-Abu^1^-containing analogue of **3** (Figure 7), with the *m*/*z* of the [M + H]^+^ and [M – H]^−^ being within 1.1 ppm of the proposed exact masses. The accurate masses of **1** and **2** correspond to the addition of C_2_H_3_NO and C_3_H_5_NO, respectively, to **3** (Table 1, Figure 7), indicative of the addition of Gly and Ala to the carboxy terminus of **3** (Figure 7). This was confirmed by the presence of a product-ion at *m*/*z* 286.1756 (C_13_H_24_N_3_O_4_^+^, Δ –1.8 ppm; from Mdha^7^–d-Ala^1^–Leu^2^, ion L in Figure 7) in **3**, but which was replaced by product-ions at *m*/*z* 343.1975 (C_15_H_27_N_4_O_5_^+^, Δ –0.3 ppm; from Mdha^7^–d-Ala^1^–Leu^2^–Gly^3^) and 357.2132 (C_16_H_29_N_4_O_5_^+^, Δ –0.2 ppm; from Mdha^7^–d-Ala^1^–Leu^2^–d-Ala^3^) in the HRMS/MS spectra of **1** and **2**, and by *m*/*z* 300.1916 (C_14_H_26_N_3_O_4_^+^, Δ –0.7 ppm; from Mdha^7^–d-Abu^1^–Leu^2^) in the spectrum of **5** (Figures S33 and S34).

In addition to **5** and **13** (above), two of the remaining detected analogues in Figure 1 and Table 1 (**15** and **17**) exhibited elemental compositions, MS^2^ (Figures S10, S12, S15, and S16) and HRMS/MS spectra (Figures S35–S42) indicative of substitution of the d-Ala at position-1 of **14** and **16**, respectively, with d-Abu (Table 1, Figure 1). For example, LC–HRMS/MS of **15** (Figures S35–S38) showed neutral loss of 114.1051 Da (C_7_H_14_O, Δ –5.7 ppm) (Figures S10 and S36) and product-ions at *m*/*z* 115.1117 (C_7_H_15_O^+^, Δ –0.4 ppm; ion A), 135.1167 (C_10_H_15_^+^, Δ –1.1 ppm; ion B), 163.1116 (C_11_H_15_O^+^, Δ –0.9 ppm; ion H), 245.1897 (C_17_H_25_O^+^, Δ –1.1 ppm; ion I), 375.1910 (C_20_H_27_N_2_O_5_^+^, Δ –1.2 ppm; ion C), and 579.3862 (C_29_H_51_N_6_O_6_^+^, Δ –0.4 ppm; ion G), consistent with an Atda^5^-containing microcystin (Table S2). However, product-ions at *m*/*z* 141.1022 and 169.0971 (C_7_H_13_N_2_O^+^ and C_8_H_13_N_2_O_2_^+^, Δ –0.6 and −0.3 ppm; both from Mdha^7^–d-Abu^1^), 460.2437 (C_24_H_34_N_3_O_6_^+^, Δ –2.4 ppm; ion D), 567.3242 (C_25_H_43_N_8_O_7_^+^, Δ –1.3 ppm; ion F), and 739.3722 ([M + H – C_17_H_30_O]^+^, Δ –1.5 ppm, compared to 725.3565 ([M + H – C_17_H_30_O]^+^, Δ –1.1 ppm for **14**) revealed an extra CH_2_ unit in the amino acid at position-1, consistent with [d-Abu^1^,Atda^5^]MC-LR (**15**), and similar observations showed that **17** was [d-Abu^1^,Ahda^5^]MC-LR.

Four peaks were observed in the full-scan LC–HRMS chromatograms with accurate masses consistent with those of monodemethylated analogues of **11** (*m*/*z* 981.5404). However, when the positive mode LC–HRMS/MS chromatogram of the precursor ion at *m*/*z* 981.5 was extracted for pairs of methylated/demethylated product-ions at *m*/*z* 135.0804/121.0647 (ion A), 135.1168/121.1011 (ion B), 375.1914/361.1758 (ion C), 599.3552/585.3395 (ion G), 258.1852/244.1696 (ion J), and 265.1587/251.1430 (ion I) (Table S2), it revealed the presence of at least six congeners (**4**, **6**, **8**–**10**, and **12**) (Figures S43 and S44). The HRMS/MS spectra of these (Figures S44–S48) were analyzed in an attempt to identify them.

Compound **12** had the same retention time as, and an identical product-ion spectrum to, a standard of [Dha^7^]MC-LR. Compound **4** was identified based on its early elution relative to **11** and from its HRMS/MS spectrum (Figures S43 and S45–S48). In particular, **4** showed neutral loss of 120.0574 Da (C_8_H_8_O, Δ –1.2 ppm) rather than 134.0732 as in **11**, ions G and J were at *m*/*z* 585.3389 (C_30_H_45_N_6_O_6_^+^, Δ –1.0 ppm; from Arg^4^–DMAdda^5^– d-Glu^6^) and 244.1691 (C_16_H_22_NO^+^, Δ –1.8 ppm) rather than at *m*/*z* 599.3552 (C_31_H_47_N_6_O_6_^+^) and 258.1850 (C_17_H_24_NO^+^, Δ –1.0 ppm) as in **11**, while ions B–F, H, and I were at the same *m*/*z* as in **11**. A very low-intensity product-ion was present at *m*/*z* 135.0803, as was also observed during the in-source fragmentation of **11** discussed above, and a very weak product-ion was present at *m*/*z* 121.0647 (C_8_H_9_O^+^, Δ –5.1 ppm) attributable to ion A of **4**. The LC–HRMS/MS spectrum of **4** was essentially identical to that of a purified specimen of [DMAdda^5^]MC-LR (C. O. Miles and A. J. Foss, unpublished data) obtained from *Nostoc* sp. 152^5^ in a recent study.^33^ [d-Asp^3^]MC-LR (**8**) has previously been identified in extracts from this heterologous expression system.^20^ The peak in the sample at ca. 7.03 min eluted at the same retention time as the standard of **8** and exhibited weak product-ions at *m*/*z* 375.1911 (C_20_H_27_N_2_O_5_^+^, Δ –1.0 ppm; ion C), 539.2936 (C_23_H_39_N_8_O_7_^+^, Δ 0.0 ppm; ion F), and 599.3555 (C_31_H_47_N_6_O_6_^+^, Δ –0.7 ppm; ion G) (Figures S43–48), consistent with the presence of low levels of **8** in the sample.

The presence of three other demethylated congeners of MC-LR (**6**, **9**, and **10**), in addition to the more commonly observed DMAdda^5^-, d-Asp^3^-, and Dha^7^-variants (**4**, **8**, and **12**) identified above, was indicated by the presence of peaks for product-ion J at *m*/*z* 244.1694 at 6.88 and 7.07 min and at *m*/*z* 258.1850 at 7.01 min. While a peak at *m*/*z* 258.1852 would be expected at this retention time from **8** (Table S2), the observed peak was much larger than would be consistent with the low levels of **8** and, furthermore, occurred at the same retention time as a correspondingly sized peak at *m*/*z* 251.1430 (ion I) indicative of demethylation on the Adda side chain (Figure S44 and Table S2). Thus, the peak at 7.03 min can be attributed to two overlapping analogues of **11** that are demethylated in the Adda side chain between C-2 and C-8, while the peak at 6.88 min contains a third such analogue.

The peak for monodemethylated MC-LR at 6.88 min (**6**) showed product-ions at *m*/*z* 135.0803 (ion A), 121.1011 (C_9_H_13_^+^, Δ –0.9 ppm; ion B), 251.1426 (C_18_H_19_O^+^, Δ –1.7 ppm; ion I), and *m*/*z* 244.1690 (C_16_H_22_NO^+^, Δ –2.3 ppm; ion J), as well as ions C–H, that were consistent with demethylation of Adda at either C-6 or C-8 (Figures S43–S48). These two sites of demethylation cannot be differentiated from the available mass spectral data without the identification of additional diagnostic fragmentation pathways within the Adda side chain. The peak at 7.07 min (**10**) contained the same product-ions as **6** and is also therefore either 6- or 8-demethylated MC-LR. In contrast, however, the peak at 7.01 min (**9**) contained the same diagnostic product-ions as **6**, except that the product-ion attributable to ion J occurred at *m*/*z* 258.1848 (C_17_H_24_NO^+^, Δ –1.6 ppm) rather than at 244.1690 (C_16_H_22_NO^+^, Δ –2.5 ppm) in the overlapping peak for **10** (Figure S44 and Table S2). Thus, this peak is attributed to an analogue of MC-LR in which the methyl group at C-2 of the Adda moiety is absent, i.e., [2-dmAdda^5^]MC-LR (**9**), while **6** and **10** are attributed to either [6-dmAdda^5^]MC-LR or [8-dmAdda^5^]MC-LR. These are not to be confused with [DMAdda^5^]MC-LR (**4**), which could be considered [9-dmAdda^5^]MC-LR (the 9-hydroxy variant of Adda) now that multiple demethylated Adda variants have been definitively identified.

One microcystin analogue (**7**) did not react with mercaptoethanol, indicating the absence of the thiol-reactive double bond present in position-7 of most microcystins.^9^ The elemental composition of this compound corresponded to the addition of H_2_O to MC-LR (**10**), which, taken together with its lack of thiol reactivity and shorter retention time, suggested this to be the *N*-methylserine-containing congener [Mser^7^]MC-LR (**7**). Consistent with this, the HRMS/MS spectrum of **7** (Figures S49–S52) contained product-ions A, B, D, E, G, and H at the same *m*/*z* as for **11**, while ions C and F were at *m*/*z* 393.2015 (C_20_H_29_N_2_O_6_^+^, Δ –1.4; from Adda^5^–d-Glu^6^–Mser^7^ – C_9_H_10_O) and 571.3197 (C_24_H_43_N_8_O_8_^+^, Δ –0.3 ppm; from Mser^7^–d-Ala^1^–Leu^2^–d-Masp^3^–Arg^4^). Partial water losses from ions C and F (to give product-ions at *m*/*z* 375.1913 and 553.3088) were also visible in the spectrum of **7**. These results confirmed its identity as [Mser^7^]MC-LR (**7**).

### Biosynthetic and Mass Spectrometric Considerations

#### Adda Modifications

Biosynthetic studies with isotopically labeled precursors reported by Moore et al.^34^ have shown that, in *M. aeruginosa*, the phenyl ring and terminus of the Adda side chain (C-9–C-16) were produced by incorporation of C-2–C-9 of l-phenylalanine and that the C-3–C-9 backbone of Adda was formed from acetate. And while it was found that C-1–C-2 and the 2-methyl group could be assembled from acetate and the methyl group of methionine, their results suggested that this substructure was preferentially formed from another precursor, possibly propionate, when exogenous acetate was limited. The 6-, 8-, and 9-*O*-methyl groups of Adda were found to originate from the methyl of l-methionine.

Here, we have identified five unusual modifications of the Adda side chain, detected in seven of the minor analogues (**6**, **9**, **10**, and **14**–**17**), produced by the heterologous host. Four of these (**14**–**17**) did not contain the characteristic phenyl ring (Figure 1) and are the first microcystin analogues identified that lack a phenyl group at C-10 of the Adda side chain. These four analogues constituted approximately 6% of the total microcystins identified, so they are minor but readily detectable products of biosynthesis in this system.

The two modified Adda backbone types contain an unsaturated butyl or hexyl chain attached to C-10. Although it is not possible from HRMS/MS data alone to determine whether these appended alkanes are branched or not, they may be formed by an extension of the C-1–C-10 side chain via addition of acetate units, and if so, they would likely be straight chain alkanes as depicted in Figure 6. Alternatively, low levels of endogenous phenylalanine could potentially result in the incorporation of nonaromatic amino acids by McyG. The Val227 residue of McyG is thought to be critical for selecting hydrophobic substrates,^35^ and ATP–PPi exchange assays have shown the capacity of McyG to incorporate a variety of substrates at varying efficiencies including substrates lacking α-amino groups.^16^ It remains unclear to what extent McyG can incorporate nonphenylated compounds. The unusual Adda side chains seen in this study could arise from relaxation of the substrate specificity of McyG and, in turn, greater variation within the Adda side chain. However, we have never detected any Atda^5^- or Ahda^5^-containing microcystins in any of the bloom or culture samples analyzed in our laboratories, not even in trace amounts, despite using sensitive untargeted LC–HRMS/MS detection methods. This suggests that the incorporation of phenylalanine is the dominant pathway in microcystin biosynthesis when this amino acid is available.

Three additional MC-LR variants containing C-demethylations on the Adda^5^ side-chain (**6**, **9**, and **10**) were identified in this study. Due to the more detailed understanding of the origin of the fragmentations involving the Adda^5^ side-chain, obtained by analysis of the product-ion spectra of **14**–**17**, the positions of these demethylations can be ascribed with high certainty to the C-2, C-6, or C-8 positions on the side chain. However, the LC–HRMS/MS data were unable to differentiate two of the potential sites of demethylation (C-6 and C-8) because no mass spectral cleavages were identified in this part of the structure. Nevertheless, a peak attributable to the 2-demethylAdda-congener of MC-LR (**9**) was identified through mass spectral cleavages leading to changes in *m*/*z* for ions B and H–J. These [dmAdda^5^]MC-LR analogues appear to correspond directly to peaks of [Leu^1^,dmAdda^5^]MC-LR isomers identified recently in a bloom from Poplar Lake, USA,^23^ and of [dmAdda^5^]MC-LR and [dmAdda^5^]MC-(H4)YR isomers detected in a dietary supplement.^12^ For example, retrospective analysis of LC–HRMS/MS data from Miller et al.^12^ for demethylated MC-LR (Figure S53) revealed product-ions identified in the present study indicative of the presence of **6** and **9** (compounds **37** and **38** in Figure 1 of Miller et al.^12^) along with traces of **10**. This suggests the same three Adda-demethylations (i.e., 2-, 6-, and 8-dmAdda^5^-congeners) identified in the present study are likely to be present at low levels in many cyanobacterial blooms, in addition to the more commonly reported demethylations via the d-Asp^3^-, DMAdda^5^-, and Dha^7^-congeners. Most likely the dmAdda^5^- and DMAdda^5^-congeners are the result of partial failure of C- and O-methylation in the C-2–C-9 region of the Adda^5^ moiety during biosynthesis, possibly due to enzymatic inefficiency or to limited availability of the methyl group donor *S*-adenosylmethionine (SAM).

[DMAdda^5^]MC-LR (**4**) is an analogue that lacks the normal 9-*O*-methylation of the Adda side chain (Figure 1) and was first detected in a cyanobacterial bloom from Homer Lake, IL, USA.^36^ McyJ is responsible for the methylation of the Adda side chain, and the presence of [DMAdda^5^]MC-LR suggests the possible partial inactivation of *mcyJ*. Previous research has shown that a disruption of *mcyJ* resulted in a shift in production to [d-Asp^3^,DMAdda^5^]MC-RR from [d-Asp^3^]MC-RR.^17,37^ However, when sufficiently sensitive analytical methods are used, culture and bloom material typically contains the DMAdda^5^ variant at approximately 0.5–1% of the corresponding nondemethylated congener, presumably due to incomplete *O*-methylation.^9^

#### Other Microcystin Variants

Two analogues with modification of the Mdha at position-7 were detected. [Mser^7^]MC-LR (**7**) has *N*-methylserine in place of Mdha, while [Dha^7^]MC-LR (**12**) has dehydroalanine in place of Mdha at that position. The incorporation of Dha at position-7 instead of Mdha is common in *Microcystis* and *Planktothrix* strains, and a study by Fewer and co-workers showed that naturally occurring in-frame deletions of the *N*-methyltransferase (NMT) domain in McyA resulted in the production of Dha and no Mdha in some *Anabaena* species.^38^ It is hypothesized that the presence of both Mdha and Dha could be the result of inaction of the NMT in these strains or, potentially, the limitation of SAM. Their study also showed that some species had low levels (up to 3%) of *N*-methylserine (Mser) in strains producing Mdha,^38^ as was also seen for the engineered *E. coli* strain in this study.

In total, [d-Abu^1^]-containing variants accounted for about 17% of the detected microcystins in the extract of the transformed *E. coli* culture, with **13** being the second most abundant analogue (ca. 15%). α-Aminobutyric acid (or homoalanine) is a nonproteinogenic amino acid produced through the transamination of 2-oxobutyrate^39^ or the reduction of the α-keto acids.^40^ Wild-type *E. coli* is capable of converting administered 2-oxobutyrate into l-homoalanine at low levels, and overexpression of the candidate aminotransferase IlvE resulted in a 6.5-fold increase in l-homoalanine.^41^ The epimerization of l-alanine to the d-form in microcystin biosynthesis is conducted by the C-terminal epimerase domain of McyA^42^ and is predicted to carry the same function on substituted amino acids.

Analogues **1**–**3** and **5** appear to be partially formed, uncyclized microcystins consisting of (DMAdda-NH_2_)^5^–d-Glu^6^–Mdha^7^–d-Ala^1^–Leu^2^-OH (**3**) (or with −d-Abu^1^– in the case of **5**), or in the cases of **1** and **2**, peptide-**3** appears to be terminated with Gly^3^-OH and Ala^3^-OH, respectively. Although compounds **3** and **5** were outside the scan range used for the thiol derivatization experiment, and so were not initially detected as potential microcystin candidates, retrospective examination of chromatograms of the derivatized sample showed the presence of peaks with the correct *m*/*z* and isotope patterns for the mercaptoethanol/*d*_4_-mercaptoethanol derivatives at appropriate retention times. While the possibility that **1**–**3** and **5** result from degradation reactions of **11** and **13** inside the *E. coli* cells cannot be excluded, it should be noted that microcystins are generally regarded as being relatively resistant to enzymatic cleavage due to their high content of d-amino acids, the presence of the β-amino acid Adda, and their cyclic structure. Furthermore, **1**–**3** and **5** contained DMAdda^5^ rather than Adda^5^, with no Adda-containing analogues of these compounds being detected in the sample. This, together with the production of Gly^3^- and Ala^3^-terminated peptides **1** and **2**, is difficult to explain through cleavages of the Adda^5^- and Masp^3^-containing cyclic peptides **11** and **13** and is consistent with an origin through interrupted biosynthesis.

#### Mass Spectrometry

Mass spectral analysis of the compounds in this study led to an improved understanding of fragmentation pathways involving the Adda side chain of microcystins (Table S2). In particular, although the *m*/*z* 135.0804 product-ion (ion A) present in the positive mode MS/MS spectra of most microcystins is derived primarily from the C-9–C-14 (terminal) part of the Adda moiety, a small proportion can also be produced via fragmentations in the C-1–C-8 part, as demonstrated by the in-source fragmentation experiments. Similarly, it is clear that the *m*/*z* 135.1168 (ion B) and 163.1117 (ion H) product-ions arise from the C-2–C-8 and C-1–C-8 portions, respectively, of the Adda moiety. This differentiation facilitates the localization of modifications introduced into the Adda side chain during biosynthesis, metabolism, or degradation. For example, a number of microcystins containing demethylations (e.g., 2-, 6-, or 8-demethylations) or oxidations in their Adda moieties have recently been identified by LC–HRMS/MS, and examination of the spectra from those studies is in accord with the fragmentation pathways identified here. An ion with *m*/*z* 265.1 in the positive ionization MS/MS spectra of ADMAdda^5^-containing microcystins (ADMAdda is 9-*O*-acetylDMAdda) has been ascribed to [ADMAdda + H – HOAc – NH_3_]^+^,^43^ and it was used by Kleinteich et al.^44^ together with *m*/*z* 135 (ions A and B), in a precursor-ion approach for screening for microcystins by LC–MS/MS. The exact mass for this product-ion is *m*/*z* 265.1587 (C_19_H_21_O^+^; ion I, Figure 6 and Table S2), and we observed it in the HRMS/MS spectra of a wide variety of the Adda- and DMAdda-containing microcystins in this study, as well as previous studies,^12,21,45,46^ including in ADMAdda^5^-containing microcystins from extracts of *Nostoc* 152 (C. O. Miles and A. J. Foss, unpublished observations). Wherever modifications to the Adda moiety occurred at positions other than the 9-*O*-R group, corresponding changes were also seen in *m*/*z* for ion I. Similarly, [M + H]^+^ also showed a neutral loss of C_19_H_26_O (the majority of the Adda-type side-chain at position-5) to give a product-ion at *m*/*z* 725.3577 (C_30_H_49_N_10_O_11_^+^) for **4**, **6**, **9**–**11**, **14**, and **16**, with corresponding ions detected for the intact microcystins modified on amino acid-1, -3, or -7 (**7**, **8**, **12**, **13**, **15**, and **17**) (see Supporting Information). A fragmentation pathway that included cleavage between C-2 and C-3 of Adda^5^ and between the 2-NH of Adda^5^ and the neighboring CO of Arg^4^ at *m*/*z* 258.1852 in **11** (C_17_H_24_NO^+^; ion J, Figure 6) proved useful in identifying the positions of some modifications to the Adda side chain. These mass spectral characteristics, summarized in Table S2, are a useful aid during structural determinations or screening for microcystins by using LC–HRMS/MS.

## Conclusions

These results highlight that substitutions of amino acids can potentially comprise a significant proportion of heterologously produced microcystins, even when an intact and otherwise fully functional synthase is present in the host organism. The incorporation of nonproteinogenic amino acids can arise from enzymatic promiscuity, amino acid availability, and changes in fermentation conditions.^47,48^ Characterization of the microcystin biosynthesis proteins and optimization of fermentation conditions can also lead to the directed production of analogues. Indeed, studies with cyanobacteria containing their native microcystin synthase complexes have already shown considerable potential for modification of the microcystins produced via manipulation of the amino acids supplied to the organisms via the growth medium.^49^ Production in “unnatural” fermentation systems, such as that described here, can increase not only the yield but also the repertoire of structural variants with, as yet unknown, bioactivities.

## Materials and Methods

### Bacterial Strain

The *E. coli* strain GB05–MtaA,^20^ with the promiscuous phosphopantetheinyl transferase MtaA chromosomally integrated, was used as a host for the heterologous expression of microcystin synthetase. The strain was transformed (using the Gene Pulser Xcell Electroporation Systems, BioRad, Hercules, USA) with fosmid pFos-biTet-*mcy*, which contains the microcystin biosynthetic gene cluster driven by the bidirectional *tet*_*o*_ promoter.^50^ Suitable colonies were selected on lysogeny broth (LB) plates supplemented with ampicillin (25 μg mL^–1^), apramycin (20 μg mL^–1^), and chloramphenicol (15 μg mL^–1^).

### Fermentation

The recombinant *E. coli* strains were cultured overnight in 10 mL of LB supplemented with 15 μg mL^–1^ chloramphenicol in 50 mL Falcon tubes (Fisher Scientific) at 30 °C with shaking (200 rpm). These cultures were used to inoculate a seed culture in 10 mL M9 minimal medium supplemented with 50 μg mL^–1^l-leucine (*E. coli* GB05 is an auxotroph strain that lacks the ability to produce l-leucine *de novo*) and 15 μg mL^–1^ chloramphenicol, which was cultured at 30 °C with 200 rpm shaking overnight. Inoculating the seed culture into M9 minimal medium limits nutrient carry over into the final culture. The seed culture was used to inoculate 500 mL of M9 minimal medium (in 2 L Erlenmeyer flasks) supplemented with 50 μg mL^–1^l-leucine, 15 μg mL^–1^ chloramphenicol, and 250 μg mL^–1^dl-*threo*-β-methylaspartic acid (Sigma–Aldrich). After inoculation, hundred-fold dilutions of the cultures were incubated at 30 °C with 200 rpm shaking. When OD_600_ reached 0.4, the cultures were incubated at 18 °C with 200 rpm shaking. Once the OD_600_ reached 0.5, the recombinant cells were induced with 0.5 μg mL^–1^ tetracycline for the expression of *mcy* genes. The *E. coli* cells were incubated for another 4 d before harvesting by centrifugation at 4000*g* for 30 min. The supernatant was transferred to a clean flask, and Amberlite XAD-7 polymeric resin (Sigma–Aldrich) was added (2% w/v) 24 h prior to harvest to absorb extracellular metabolites with 200 rpm shaking at 18 °C. The resin was harvested by centrifugation at 4000*g* for 30 min, separated from the supernatant, and stored at −20 °C prior to toxin extraction.

### Extraction of Microcystins

Extraction of microcystins from cell pellets and resin was conducted using 80% aqueous MeOH. First, 6 mL of Milli-Q water was added to either cell pellets or resin (thawed on ice prior to extraction) harvested from 500 mL of culture, followed by vortex-mixing for resuspension. After 24 mL of MeOH was added, the mixture was vortex-mixed for 2 min followed by shaking (200 rpm) for 1 h at ambient temperature. Centrifugation (4000*g*) was subsequently applied to remove cellular debris and resin. The supernatants were filtered (Whatman No. 1, 185 mm; Merck) into Syncore dryer sample glass tubes (Buchi–Syncore, Flawil, Switzerland). Extracts were evaporated to about 3 mL with a Syncore dryer (35 °C, 200 rpm), transferred to 250 mL evaporation flasks (about 60 mL of concentrated extracts were harvested from 20 replicate 500 mL cultures), and evaporated to dryness. MeOH (20 mL) was added to the evaporation flask to dissolve the residue, and the mixture was transferred to scintillation vials and evaporated to dryness using a rotary evaporator.

### LC−MS Analyses

Certified reference materials (CRMs) of MC-LR (**11**), [Dha^7^]MC-LR (**12**), and MC-RR were obtained from the National Research Council (Halifax, NS, Canada), and standards of [d-Asp^3^]MC-LR (**8**) and MC-LA were obtained from Enzo Life Sciences (Farmingdale, NY, USA). A nonquantitative mixture of CRMs and in-house reference materials containing **8**, **11**, **12**, [Leu^1^]MC-LY, MC-LA, MC-WR, MC-YR, and MC-RY was also used. Acetonitrile and formic acid (∼98%) were LC–MS grade from Fisher Scientific (Ottawa, ON, Canada). Distilled water was ultrapurified to 18.2 MΩ·cm using a Milli-Q water purification system (Millipore–Sigma). Ammonium carbonate, mercaptoethanol, and *d*_4_-mercaptoethanol were from Sigma–Aldrich, (St. Louis, MO, USA). An extract of *M. aeruginosa* PCC 7806 was available from previous studies.^20^

### LC–MS^2^ (Method A)

Analyses were performed with a Symmetry Shield RP18 column (100 × 2.1 mm, 3.5 μm; Waters, Milford, MA, USA) held at 40 °C with mobile phases A and B of H_2_O and CH_3_CN, respectively, each of which contained formic acid (0.1% v/v). Linear gradient elution (0.3 mL min^–1^) was from 20 to 90% B over 18 min, then to 100% B over 0.1 min with a hold at 100% B (2.9 min), and then returned to 20% B over 0.1 min with a hold at 20% B (3.9 min) to equilibrate the column with eluent diverted to waste after 20 min (total run time 25 min). Injection volume was 2.5 μL (standards) or 5 μL (sample). The column was connected to an Agilent 1260 series HPLC system (Agilent Technologies, Palo Alto CA, USA) binary pump. The detector was a Thermo LTQ_XL_ ion trap mass spectrometer (Thermo Fisher Scientific, Mississauga ON, Canada) operated in positive ion ESI mode. ESI parameters were a source voltage of 3 kV, a capillary temperature of 250 °C, a sheath gas rate of 35 units N_2_ (ca. 350 mL/min), and an auxiliary gas rate of 10 units N_2_ (ca. 100 mL/min). Either alternating FS and MS^2^-scan modes or pure MS^2^-scan mode was used. In FS/MS^2^ mode, the FS was *m*/*z* 850–1200, AGC target 3 × 10^4^, and the maximum ion injection time (maxIT) was set to 10 ms with a total of five microscans, with MS^2^ on the selected precursor [M + H]^+^ ion using isolation width 1.0, collision energy (CE) 50 eV, Q 0.25, AGC target 1 × 10^4^, with the maxIT set to 100 ms with one microscan. Pure MS^2^ spectra were acquired as described above, but without the FS, and only one precursor ion was targeted per injection.

### LC–HRMS/MS (Method B)

Liquid chromatography with high-resolution MS (LC–HRMS) used a Q Exactive HF Orbitrap mass spectrometer equipped with a HESI-II heated electrospray ionization interface (ThermoFisher Scientific, Waltham, MA, USA) connected to an Agilent 1200 LC system including a binary pump, autosampler, and column oven (Agilent, Santa Clara, CA, USA). The column, mobile phases, and gradient were as described above for LC–MS^2^ (method A), and the injection volume was 1–5 μL, depending on the sample.

In positive ion mode the mass spectrometer was calibrated from *m*/*z* 74–1622, the spray voltage was 3.7 kV, the capillary temperature was 350 °C, and the sheath and auxiliary gas flow rates were 25 and 8 units, respectively, with MS data acquired from 2–20 min. Mass spectral data was collected using a combined FS/DIA method. FS data was collected from *m*/*z* 500–1400 using the 60,000 resolution setting, an AGC target of 1 × 10^6^, and a maxIT of 100 ms. DIA data was collected using the 15,000 resolution setting, an AGC target of 2 × 10^5^, maxIT set to “auto”, and a stepped CE of 30, 60, and 80 eV. Precursor isolation windows were 62 *m*/*z* wide and centered at *m*/*z* 530, 590, 650, 710, 770, 830, 890, 950, 1010, 1070, 1130, 1190, 1250, 1310, and 1370. DIA chromatograms were extracted for product-ions at *m*/*z* 121.1011, 121.0647, 135.0804, 135.1168, 375.1915, 389.2072, 361.1758, 213.0870, 426.2096, 440.2252, 454.2409, 412.1939, 393.2020, 379.1864, 585.3395, 599.3552, and 613.3709.

The [M + H]^+^ ions of putative MCs detected using the FS/DIA methods and mercaptoethanol derivatization were further probed in a targeted manner using the PRM scan mode in positive ionization mode with a 0.7 *m*/*z* precursor isolation window, typically using the 60,000 resolution setting, an AGC target of 5 × 10^5^, and a maxIT of 1000 ms. CEs were: stepped CE at 30, 60, and 80 eV for intact MCs **4** and **6**–**17**, and stepped CE at 20, 30, and 35 eV for linear MC analogues **1**–**3** and **5**. Full-scan chromatograms were obtained in MS-SIM mode as for FS/DIA but with a resolution setting of 120,000 and maxIT 300 ms.

Pseudo-MS^3^ spectra in positive ionization mode were obtained for [M + H – C_9_H_10_O]^+^ ions of: MC-LR (**11**) at *m*/*z* 995.5 → 861.4 (CE 65 eV) and at 995.5 → 375.2 (CE 26 eV); MC-RR at *m*/*z* 1038.5 → 904.5 (CE 80 eV), and; MC-LA at 910.5 → 776.5 (CE 45 eV), by in-source fragmentation of the precursor [M + H]^+^ ion followed by PRM spectra. The spectra were obtained by infusion of microcystin standards in MeOH at 3 μL/min into a stream of eluents A and B (1:1) at 0.3 mL/min. The mass spectrometer settings were as for PRM scan mode but with the in-source fragmentation energy set to 100 eV and the CE specified above for PRM of the selected in-source product-ion.

In negative mode, the mass spectrometer was calibrated from *m*/*z* 69–1780 and the spray voltage was −3.7 kV, while the capillary temperature, sheath, and auxiliary gas flow rates were the same as for positive mode. Mass spectral data was collected in FS/DIA scan mode as above using a scan range of *m*/*z* 650–1300, a resolution setting of 60,000, AGC target of 1 × 10^6^, and maxIT of 100 ms. DIA data was collected using a resolution setting of 15,000, AGC target of 2 × 10^5^, maxIT set to “auto”, and stepped CE 65 and 100 eV. Isolation windows were 45 *m*/*z* wide and centered at *m*/*z* 686, 729, 772, 815, 858, 902, 945, 988, 1032, 1075, 1118, 1162, 1205, 1248, and 1294. DIA chromatograms were extracted for product-ions at *m*/*z* 128.0353. Full-scan chromatograms were obtained in MS-SIM mode as above but with a resolution setting of 120,000, a maxIT of 300 ms, and scan range *m*/*z* 750–1400.

### Thiol Derivatization

Thiol derivatization of the extract was performed by addition of (NH_4_)_2_CO_3_ (0.1 M, 200 μL) to the filtered extract (200 μL), with 200 μL transferred to two LC−MS vials. To one vial was added 1 μL of a 1:1 mixture of mercaptoethanol and *d*_4_-mercaptoethanol, while 1 μL of water was added the other vial as a control.^12^

## Supporting Information Available

LC-HRMS chromatograms, mass spectra, product-ion spectra, fragmentation charts, and tabulated product-ions (PDF)
Table S1, tabulated product-ions (XLSX)

## Dedication

*We dedicate this manuscript to the family and memory of our dear colleague and wonderful scientist Tianzhe Liu.*
