# Supplementary material for: Microcystins with Modified Adda5-Residues from a Heterologous Microcystin Expression System
Source: ACS Omega. 2024 Jun 12;9(25):27618–31. doi: 10.1021/acsomega.4c03332 (PMC11209926; doi:10.1021/acsomega.4c03332)

Microcystins with Modified Adda^5-Residues from a heterologous microcystin expression system

Christopher O. Miles,[1,2,*] Pearse McCarron,[1] Krista Thomas,[1] Bakir Al-Sinawi, [3,5] Tianzhe Liu,[3,4,†] and Brett A. Neilan[5,6]

[1]Biotoxin Metrology, National Research Council Canada, Halifax, Nova Scotia, Canada B3H 3Z1

[2]Norwegian Veterinary Institute, Postboks 64, 1431 Ås, Norway

[3]Diagnostic Technology Pty. Ltd., NSW 2085, Sydney, Australia

[4]Department of Chemistry and Food Chemistry, Technical University of Dresden, 01069, Dresden, Germany

[5]School of Environmental and Life Sciences, The University of Newcastle, NSW 2308, Callaghan, Australia

[6]ARC Centre of Excellence in Synthetic Biology, Sydney, New South Wales, NSW 2019, Australia

[†]Deceased

Dedication

We dedicate this manuscript to the family and memory of our dear colleague and wonderful scientist Tianzhe Liu

Table of Contents

**Figure S1**	LC–HRMS chromatograms after derivatization with mercaptoethanol	S4
**Figure S2**	LC–HRMS spectra of **14**–**17** after derivatization with mercaptoethanol	S5
**Figure S3**	LC–HRMS/MS and LC–MS^2 spectra of [DMAdda^5]MC-LR (**4**)	S6
**Figure S4**	LC–HRMS/MS and LC–MS^2 spectra of [dmAdda^5]MC-LR (**6**)	S7
**Figure S5**	LC–HRMS/MS and LC–MS^2 spectra of [dmAdda^5]MC-LR (**9** + **10**)	S8
**Figure S6**	LC–HRMS/MS and LC–MS^2 spectra of MC-LR (**11**)	S9
**Figure S7**	LC–HRMS/MS and LC–MS^2 spectra of [Dha^7]MC-LR (**12**)	S10
**Figure S8**	LC–HRMS/MS and LC–MS^2 spectra of [D-Abu^1]MC-LR (**13**)	S11
**Figure S9**	LC–HRMS/MS and LC–MS^2 spectra of [Atda^5]MC-LR (**14**)	S12
**Figure S10**	LC–HRMS/MS and LC–MS^2 spectra of [D-Abu^1,Atda^5]MC-LR (**15**)	S13
**Figure S11**	LC–HRMS/MS and LC–MS^2 spectra of [Ahda^5]MC-LR (**16**)	S14
**Figure S12**	LC–HRMS/MS and LC–MS^2 spectra of [D-Abu^1,Ahda^5]MC-LR (**17**)	S15
**Figure S13**	LC–MS^2 spectra of MC-LR (**11**), and Atda/Ahda^5-Microcystins **14** and **16**	S16
**Figure S14**	LC–MS^2 spectra of MC-LR (**11**), and Atda/Ahda^5-Microcystins **14** and **16**	S17
**Figure S15**	LC–MS^2 spectra of [D-Abu^1]MC-LR (**13**) and Abu^1-MCs **15** and **17**	S18
**Figure S16**	LC–MS^2 spectra of [D-Abu^1,MC-LR (**13**) and Abu^1-MCs **15** and **17**	S19
**Figure S17**	LC–HRMS/MS spectra of *seco*MC-LR and *seco*[D-Asp^3]MC-LR	S20
**Figure S18**	LC–HRMS/MS spectra of *seco*MC-LR and *seco*[D-Asp^3]MC-LR	S21
**Figure S19**	LC–HRMS/MS spectra of *seco*MC-LR and *seco*[D-Asp^3]MC-LR	S22
**Figure S20**	LC–HRMS/MS spectra of *seco*MC-LR and *seco*[D-Asp^3]MC-LR	S23
**Figure S21**	LC–HRMS/MS spectra of MC-LR (**11**) and [D-Abu^1]MC-LR (**13**)	S24
**Figure S22**	LC–HRMS/MS spectra of MC-LR (**11**) and [D-Abu^1]MC-LR (**13**)	S25
**Figure S23**	LC–HRMS/MS spectra of MC-LR (**11**), and Atda/Ahda^5-MCs **14** and **16**	S26
**Figure S24**	LC–HRMS/MS spectra of MC-LR (**11**), and Atda/Ahda^5-MCs **14** and **16**	S27
**Figure S25**	LC–HRMS/MS spectra of MC-LR (**11**), and Atda/Ahda^5-MCs **14** and **16**	S28
**Figure S26**	LC–HRMS/MS spectra of MC-LR (**11**), and Atda/Ahda^5-MCs **14** and **16**	S29
**Figure S27**	LC–HRMS/MS/MS spectra of MC-LR (**11**) via in-source fragmentation	S30
**Figure S28**	LC–HRMS/MS/MS spectra of MC-LR (**11**) via in-source fragmentation	S31
**Figure S29**	LC–HRMS/MS/MS spectra of MC-LR (**11**) via in-source fragmentation	S32
**Figure S30**	LC–HRMS/MS/MS spectra of MC-LR (**11**), MC-RR, and MC-LA via in-source fragmentation	S33
**Figure S31**	LC–HRMS/MS spectra of microcystin analogues **1**–**3**, **5**, and MC-LA	S34
**Figure S32**	LC–HRMS/MS spectra of microcystin analogues **1**–**3**, **5**, and MC-LA	S35
**Figure S33**	LC–HRMS/MS spectra of microcystin analogues **1**–**3**, **5**, and MC-LA	S36
**Figure S34**	LC–HRMS/MS spectra of microcystin analogues **1**–**3**, **5**, and MC-LA	S37
**Figure S35**	LC–HRMS/MS spectra of Atda^5-microcystins **14** and **15**	S38
**Figure S36**	LC–HRMS/MS spectra of Atda^5-microcystins **14** and **15**	S39
**Figure S37**	LC–HRMS/MS spectra of Atda^5-microcystins **14** and **15**	S40
**Figure S38**	LC–HRMS/MS spectra of Atda^5-microcystins **14** and **15**	S41
**Figure S39**	LC–HRMS/MS spectra of Ahda^5-microcystins **16** and **17**	S42
**Figure S40**	LC–HRMS/MS spectra of Ahda^5-microcystins **16** and **17**	S43
**Figure S41**	LC–HRMS/MS spectra of Ahda^5-microcystins **16** and **17**	S44

**Figure S42**	LC–HRMS/MS spectra of Ahda^5-microcystins **16** and **17**	S45
**Figure S43**	LC–HRMS/MS chromatograms of demethylated MC-LR analogues	S46
**Figure S44**	LC–HRMS/MS spectra and chromatograms of microcystins **6**, and **8**–**10**	S47
**Figure S45**	LC–HRMS/MS spectra of demethylated MC-LR analogues **4**, **6**, **8**–**9** and **12**	S48
**Figure S46**	LC–HRMS/MS spectra of demethylated MC-LR analogues **4**, **6**, **8**–**9** and **12**	S49
**Figure S47**	LC–HRMS/MS spectra of demethylated MC-LR analogues **4**, **6**, **8**–**9** and **12**	S50
**Figure S48**	LC–HRMS/MS spectra of demethylated MC-LR analogues **4**, **6**, **8**–**9** and **12**	S51
**Figure S49**	LC–HRMS/MS spectra of [Mser^7]MC-LR (**7**) and MC-LR (**11**)	S52
**Figure S50**	LC–HRMS/MS spectra of [Mser^7]MC-LR (**7**) and MC-LR (**11**)	S53
**Figure S51**	LC–HRMS/MS spectra of [Mser^7]MC-LR (**7**) and MC-LR (**11**)	S54
**Figure S52**	LC–HRMS/MS spectra of [Mser^7]MC-LR (**7**) and MC-LR (**11**)	S55
**Figure S53**	Retrospective analysis of LC–HRMS/MS spectra and chromatograms of microcystins **6**, and **8**–**10** in an extract of a dietary supplement	S56
**Table S1**	Tabulation of assigned product ions for **4**, **6**–**8**, and **11**–**17**	Separate Excel file
**Table S2**	Characteristic Adda-related product ions in microcystins	S57

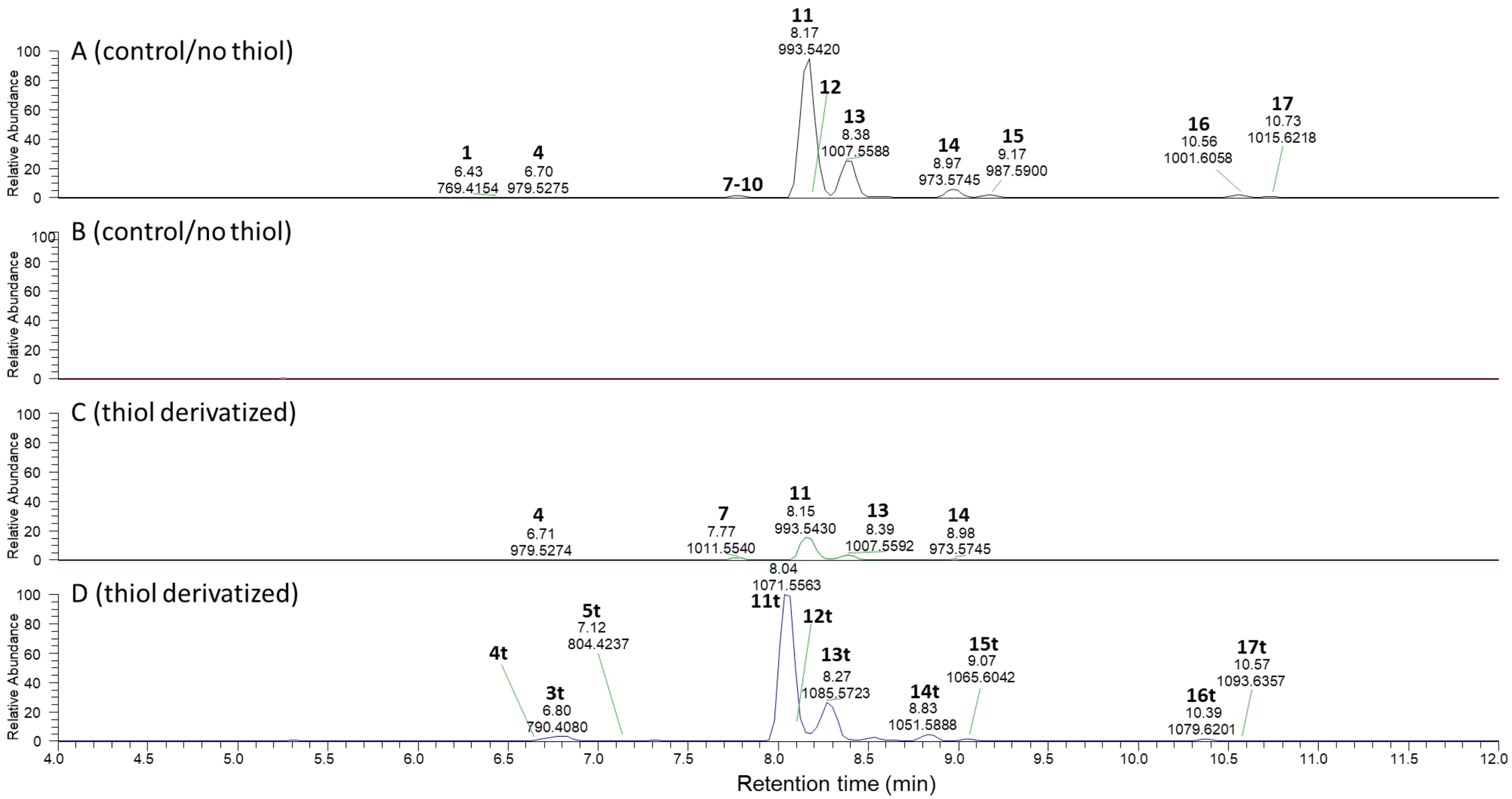

Figure S1. LC–HRMS chromatograms (method B) in negative ionization mode, extracted for exact masses for *m*/*z* of $[M - H]^-$ of **1**–**17** (Figure 1): A, before (only underivatized MCs present), and; C, after derivatization (only trace underivatized MCs remaining) with d_0-/d_4-mercaptoethanol, and; for their corresponding mercaptoethanol (sum of d_0 + d_4-derivatives) derivatives (**1t**–**17t**): B, before (no thiol derivatives present), and; D, after derivatization with d_0-/d_4-mercaptoethanol (mainly mercaptoethanol derivatives). Visible peaks are labelled with compound numbers (appended with a "t" for thiol conjugates), retention times (min), and observed *m*/*z* for $[M - H]^-$. All chromatograms are plotted with the same absolute intensity scale.

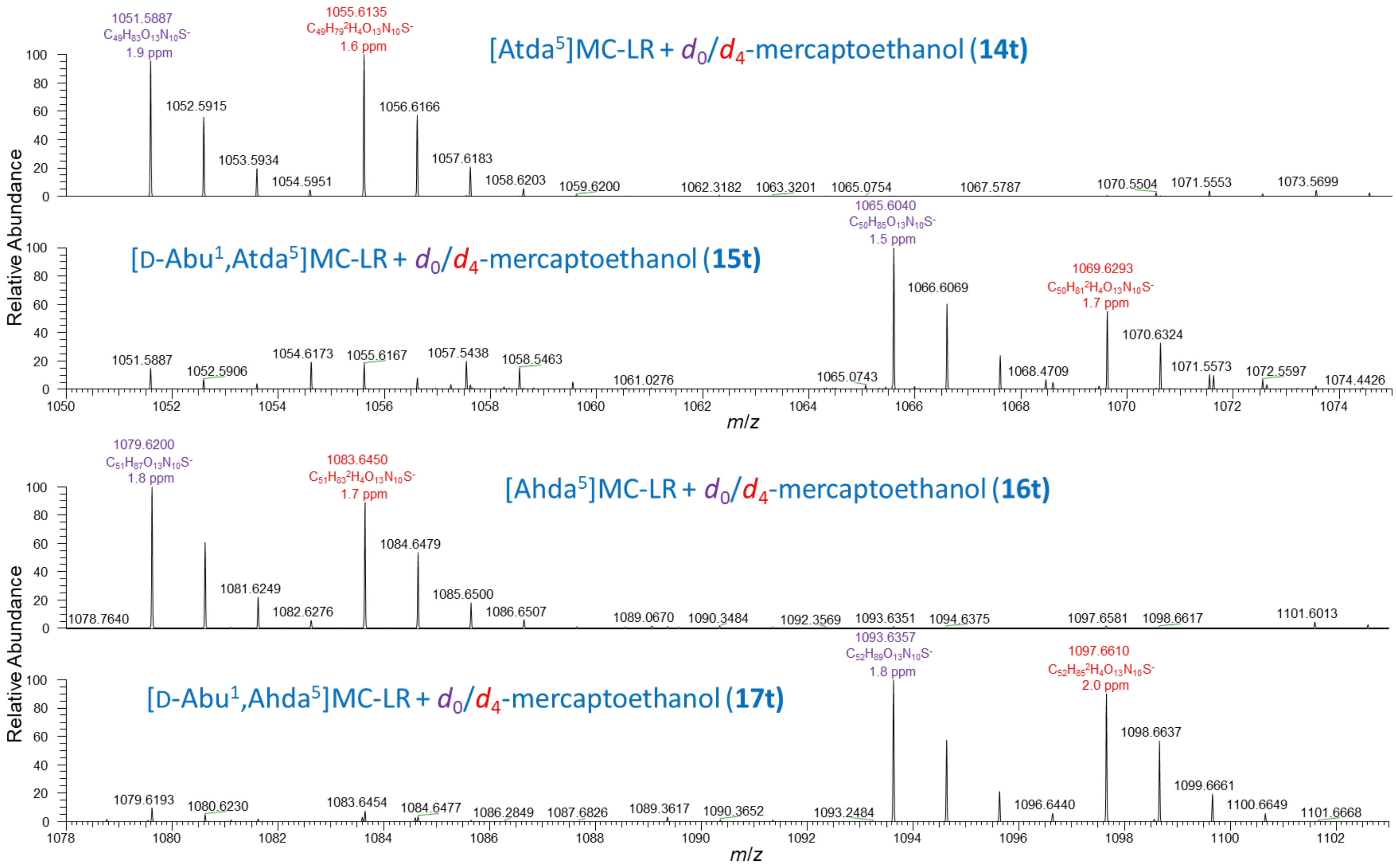

Figure S2. LC–HRMS (method B) spectra of **14t**–**17t** extracted from the chromatogram shown in Figure S1D, and produced by derivatization of **14**–**17** with 1:1 d_0-/d_4-mercaptoethanol. The accurate masses (m/z of $[M - H]^-$), assigned elemental compositions, and associated mass errors are shown for each peak (unlabeled in purple, deuterium-labeled in red).

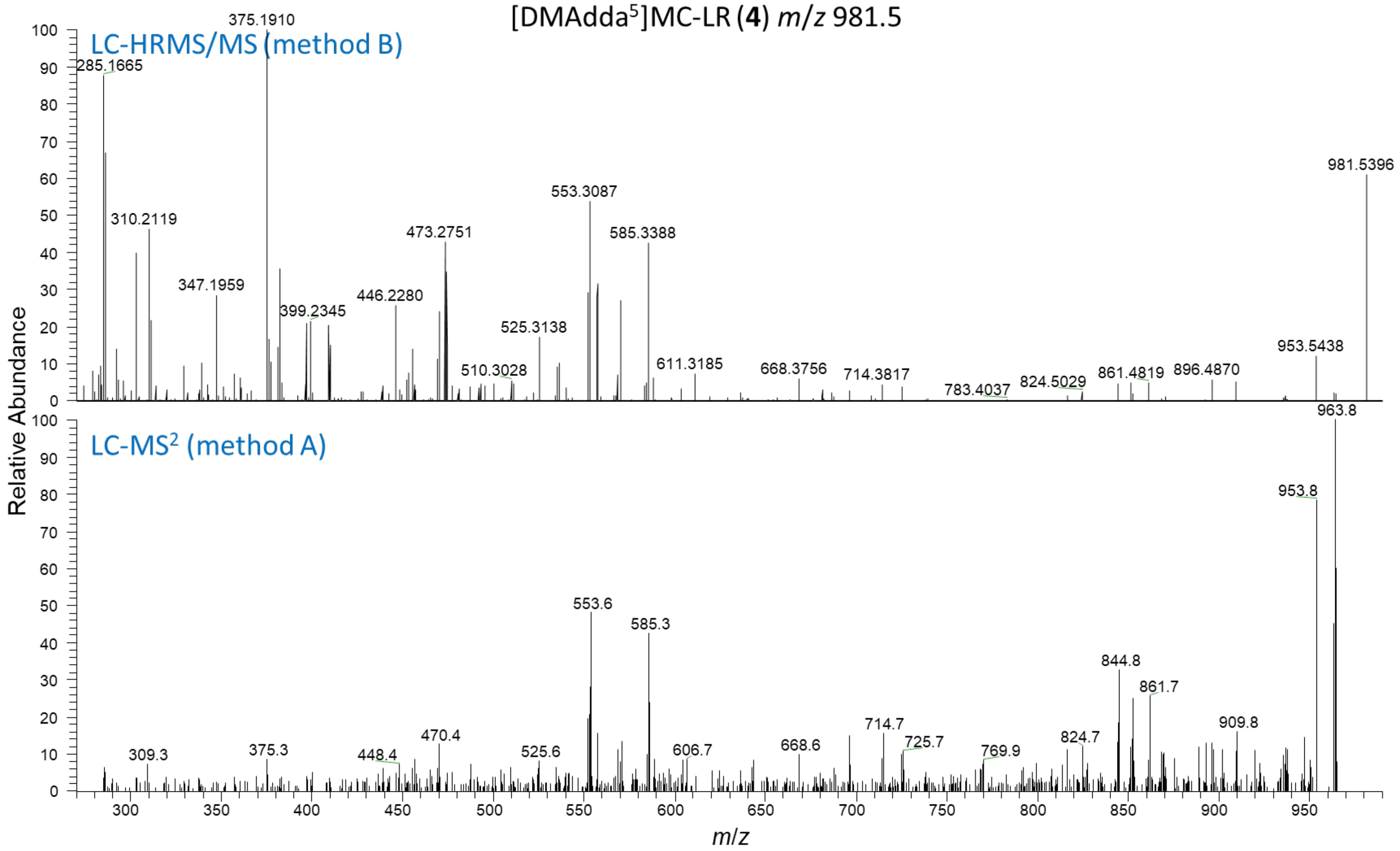

Figure S3. LC–HRMS/MS (method B) and LC–MS^2 (method A) spectra of $[M + H]^+$ of [DMAdda^5]MC-LR (**4**) in positive ionization mode.

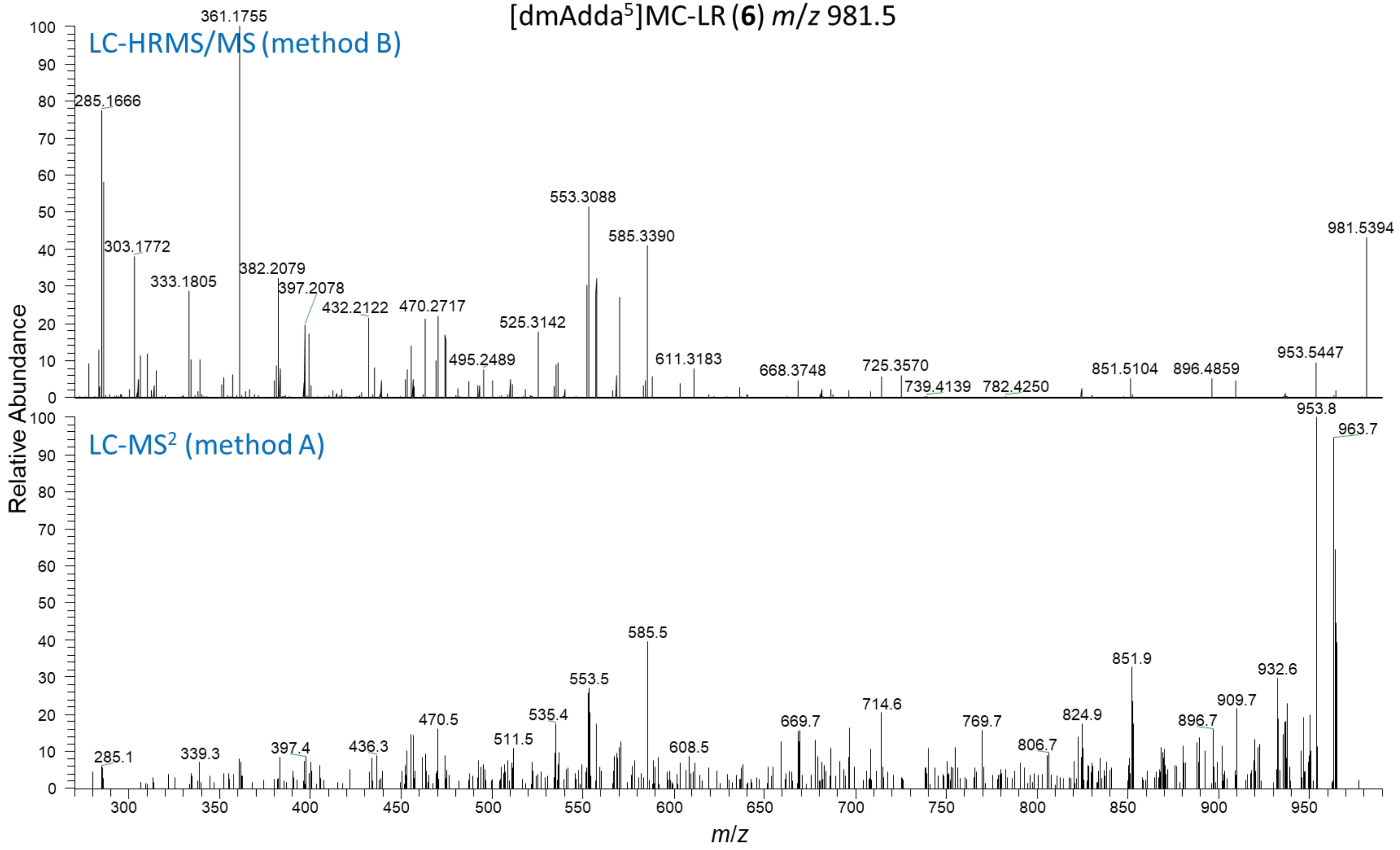

Figure S4. LC–HRMS/MS (method B) and LC–MS^2 (method A) spectra of $[M + H]^+$ of [dmAdda^5]MC-LR (**6**) in positive ionization mode.

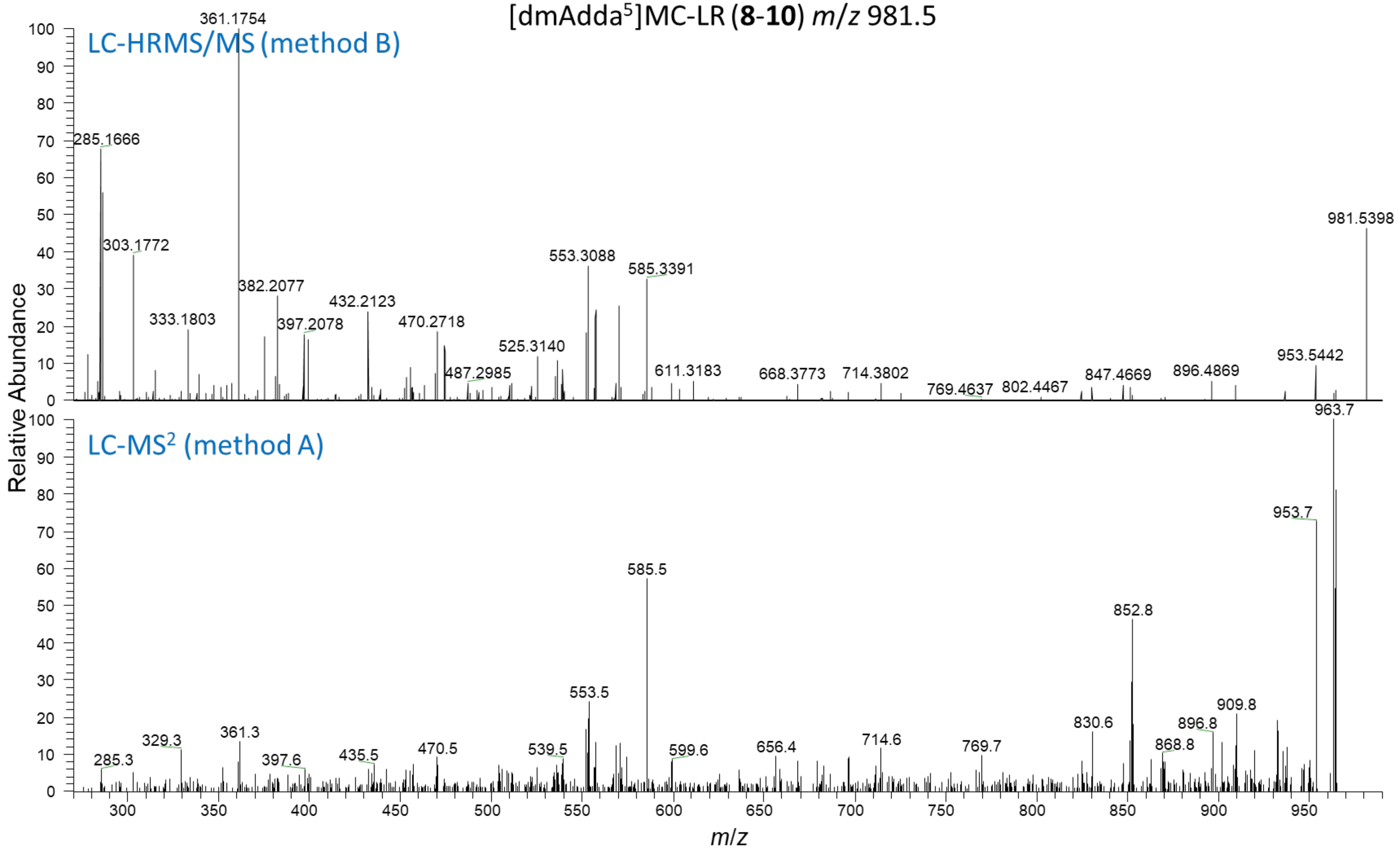

Figure S5. LC–HRMS/MS (method B) and LC–MS^2 (method A) spectra of $[M + H]^+$ of [dmAdda^5]MC-LR (**8**–**10**) in positive ionization mode.

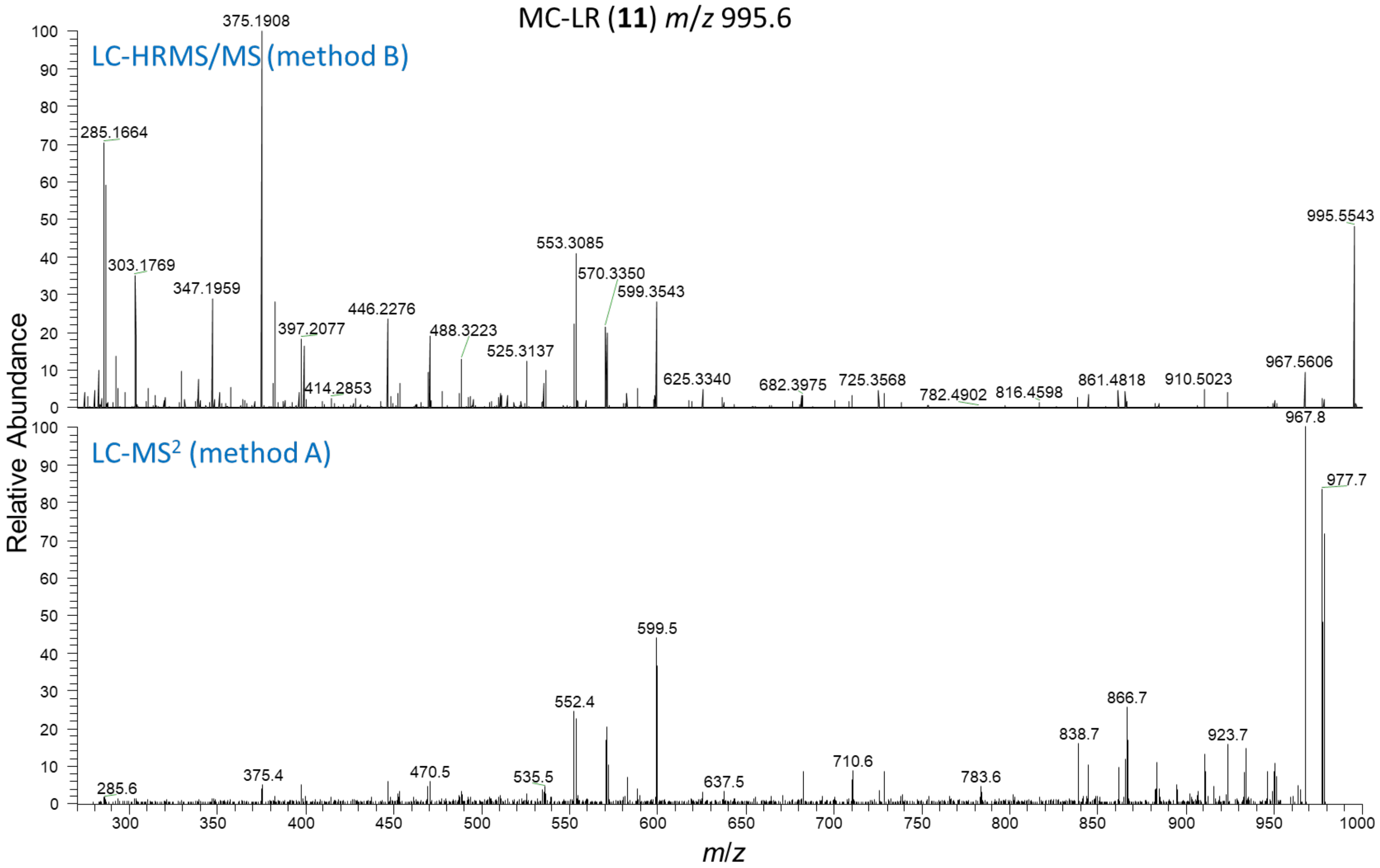

Figure S6. LC–HRMS/MS (method B) and LC–MS^2 (method A) spectra of $[M + H]^+$ of MC-LR (**11**) in positive ionization mode.

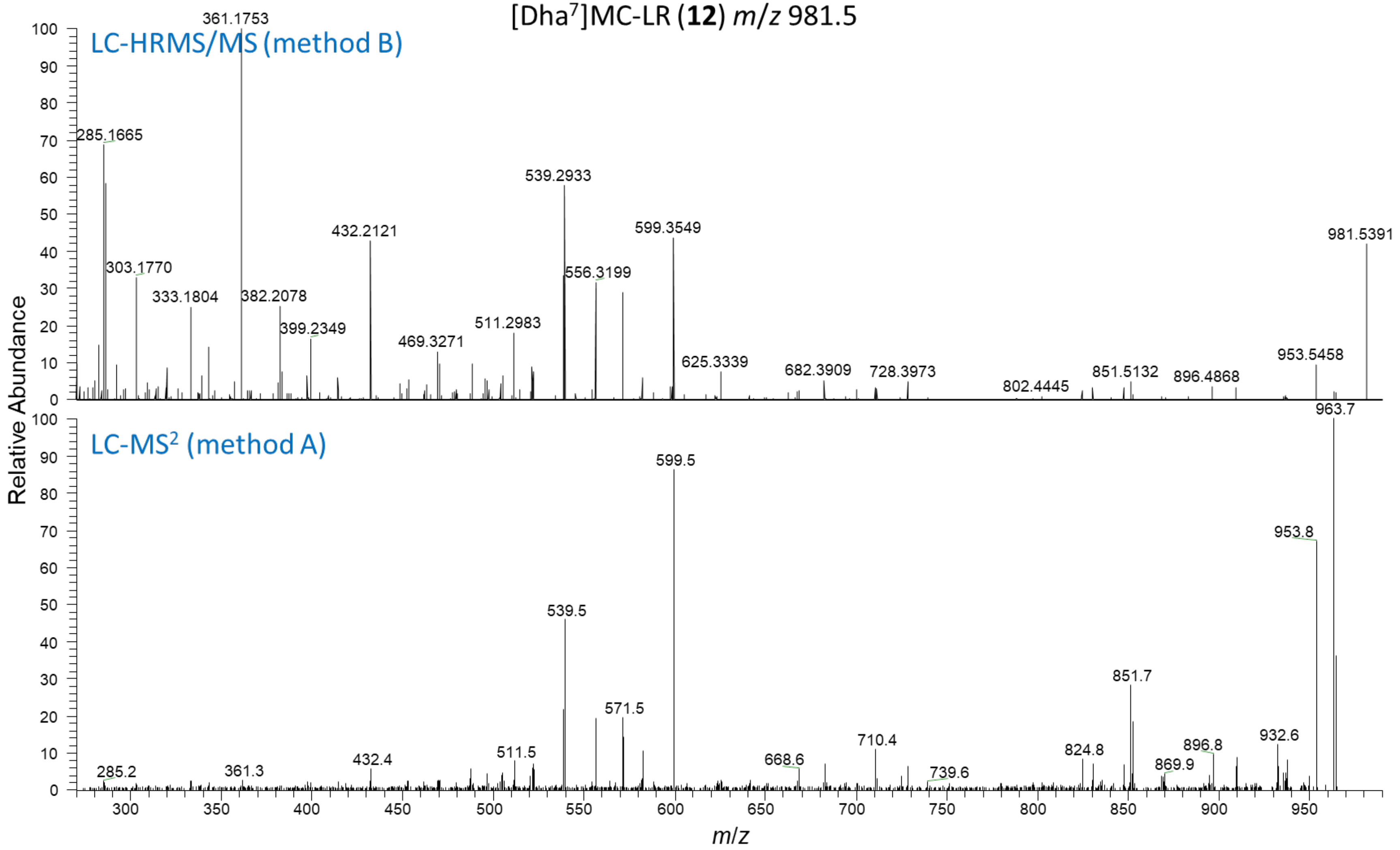

Figure S7. LC–HRMS/MS (method B) and LC–MS^2 (method A) spectra of $[M + H]^+$ of [Dha^7]MC-LR (**12**) in positive ionization mode.

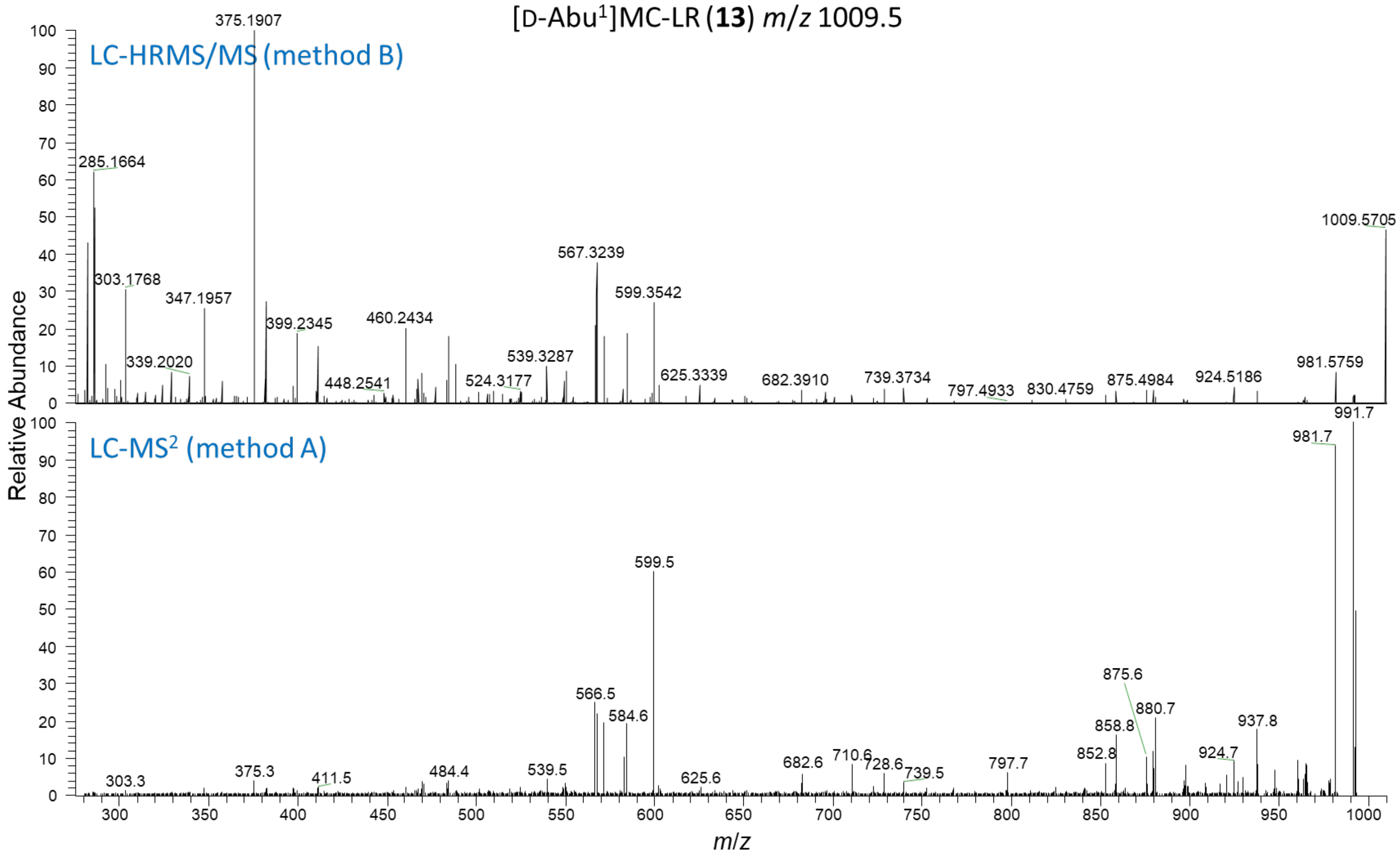

Figure S8. LC–HRMS/MS (method B) and LC–MS^2 (method A) spectra of $[M + H]^+$ of [D-Abu[1]]MC-LR (**13**) in positive ionization mode.

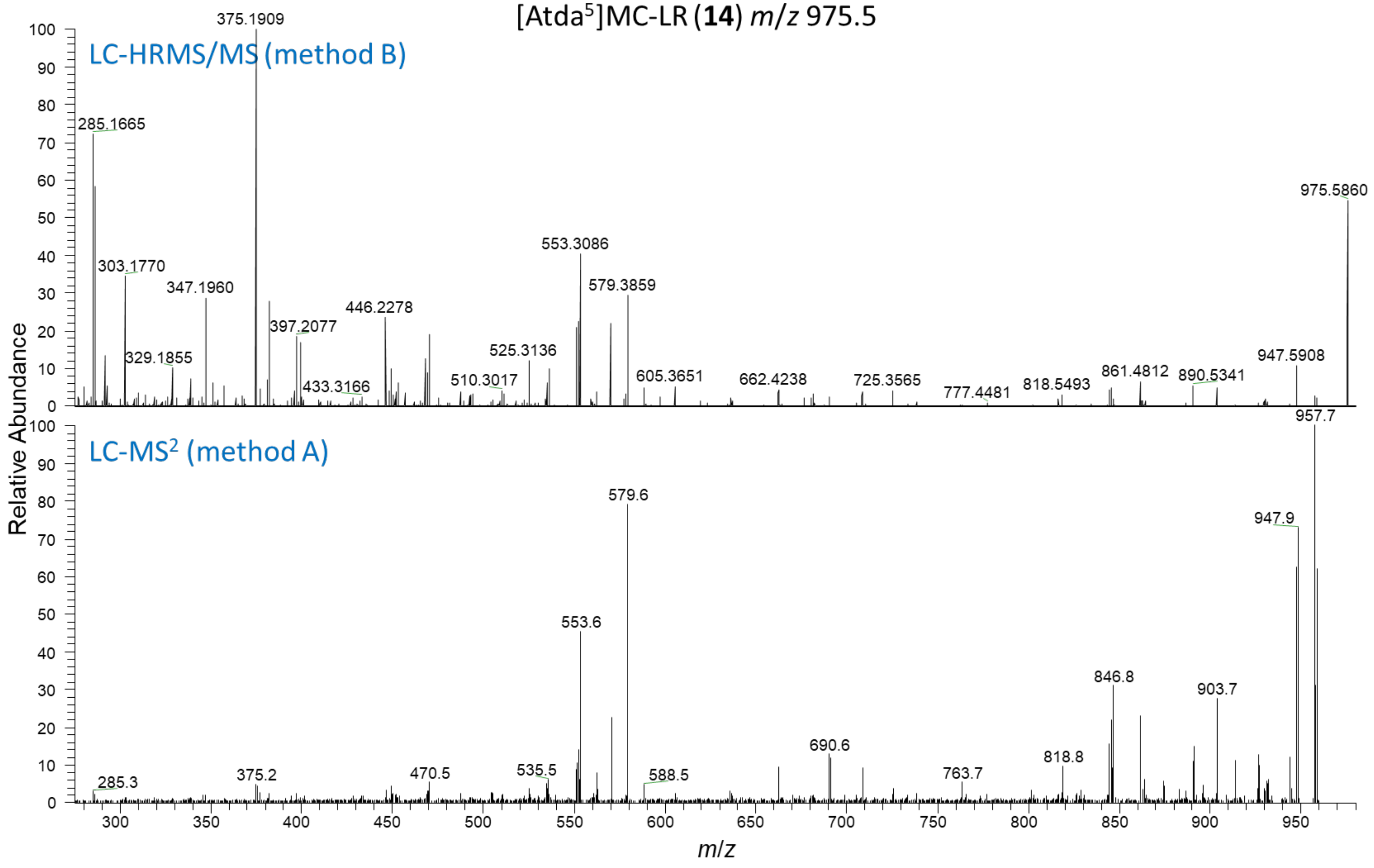

Figure S9. LC–HRMS/MS (method B) and LC–MS^2 (method A) spectra of $[M + H]^+$ of [Atda^5]MC-LR (**14**) in positive ionization mode.

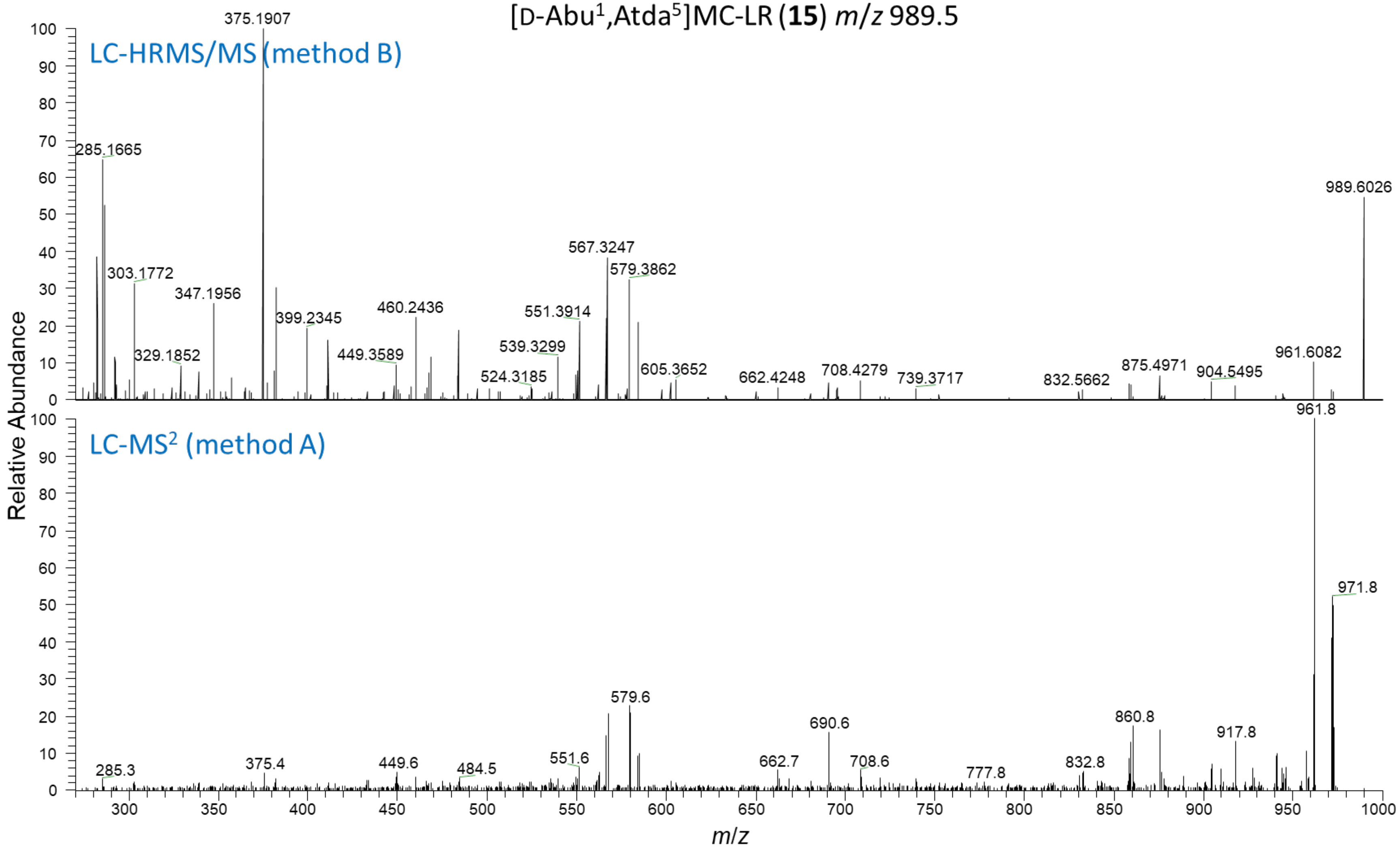

Figure S10. LC–HRMS/MS (method B) and LC–MS^2 (method A) spectra of $[M + H]^+$ of [D-Abu^1,Atda^5]MC-LR (**15**) in positive ionization mode.

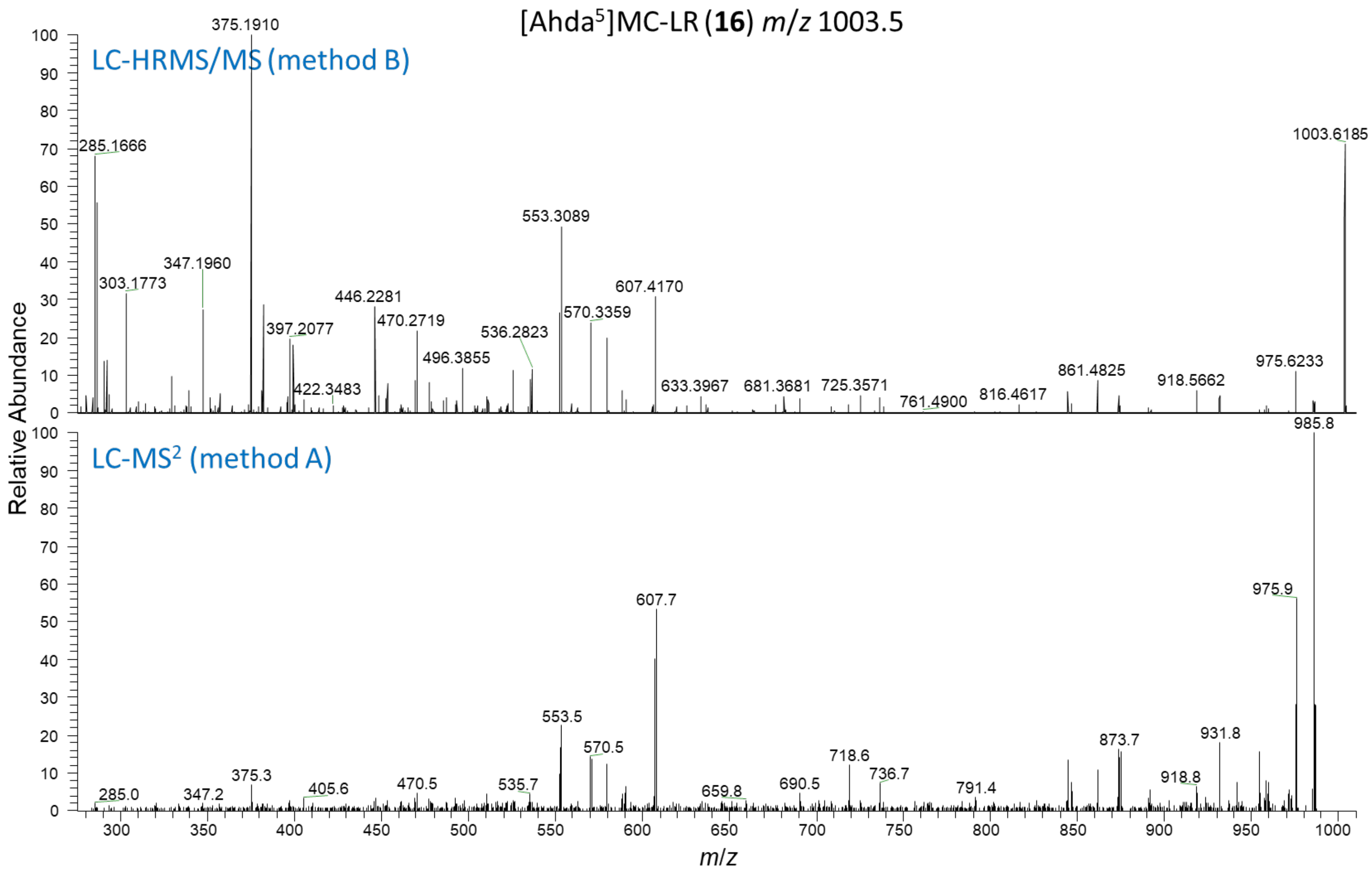

Figure S11. LC–HRMS/MS (method B) and LC–MS^2 (method A) spectra of $[M + H]^+$ of [Ahda^5]MC-LR (**16**) in positive ionization mode.

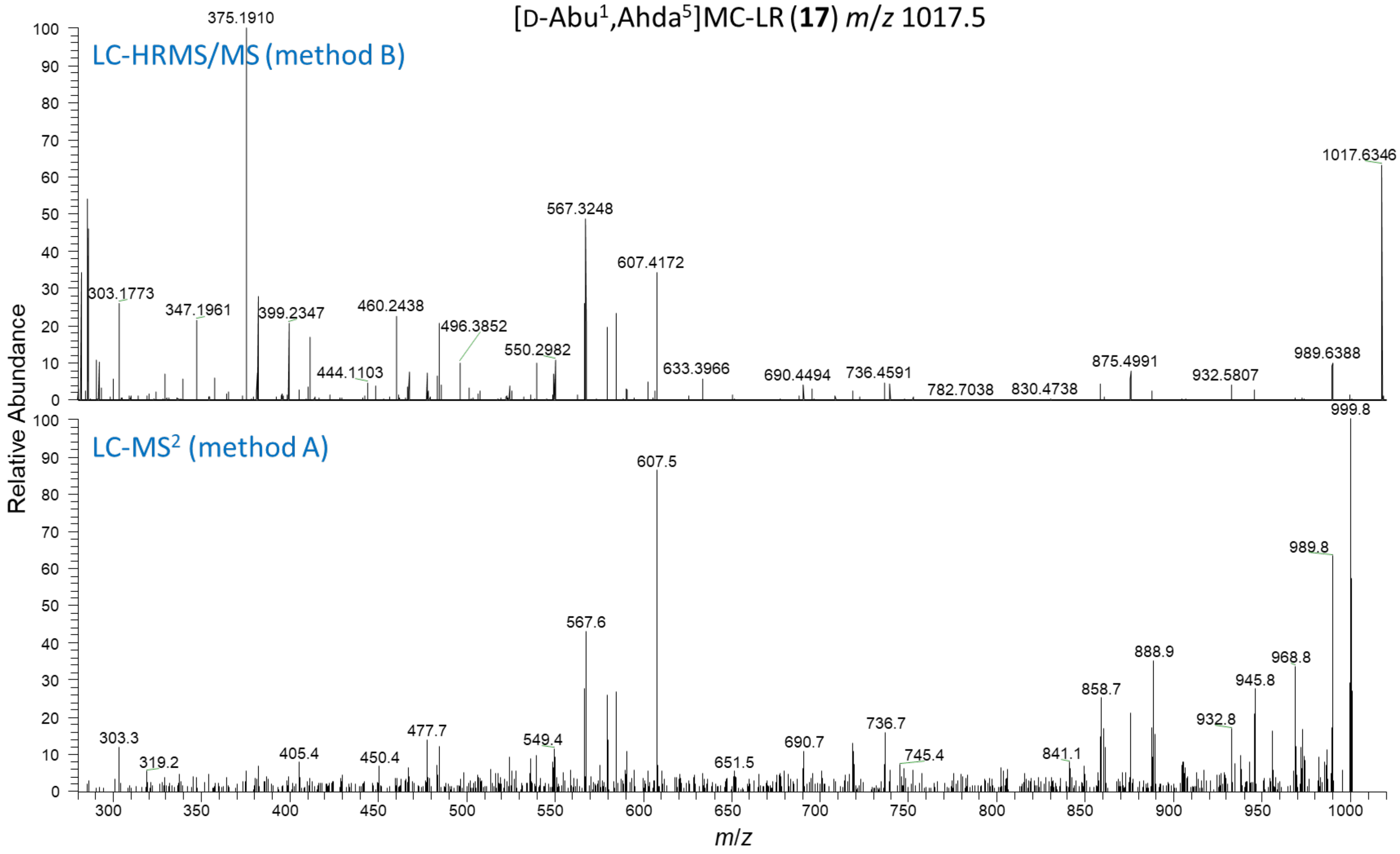

Figure S12. LC–HRMS/MS (method B) and LC–MS^2 (method A) spectra of $[M + H]^+$ of [D-Abu[1],Ahda[5]]MC-LR (**17**) in positive ionization mode.

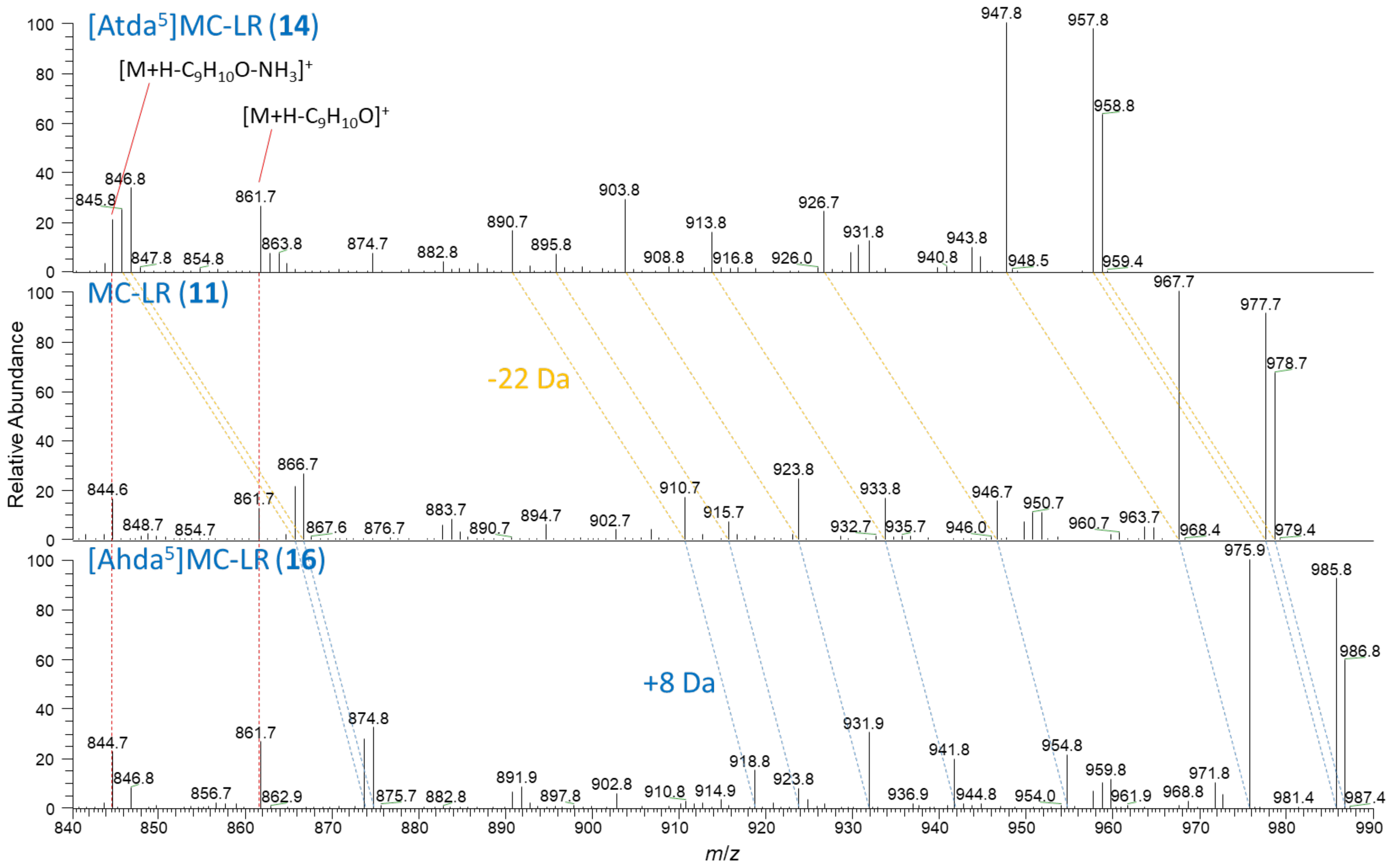

Figure S13. LC–MS^2 (method A) spectra of $[M + H]^+$ of: top, [Atda^5]MC-LR (**14**); middle, MC-LR (**11**), and; bottom, [Ahda^5]MC-LR (**16**), in positive ionization mode.

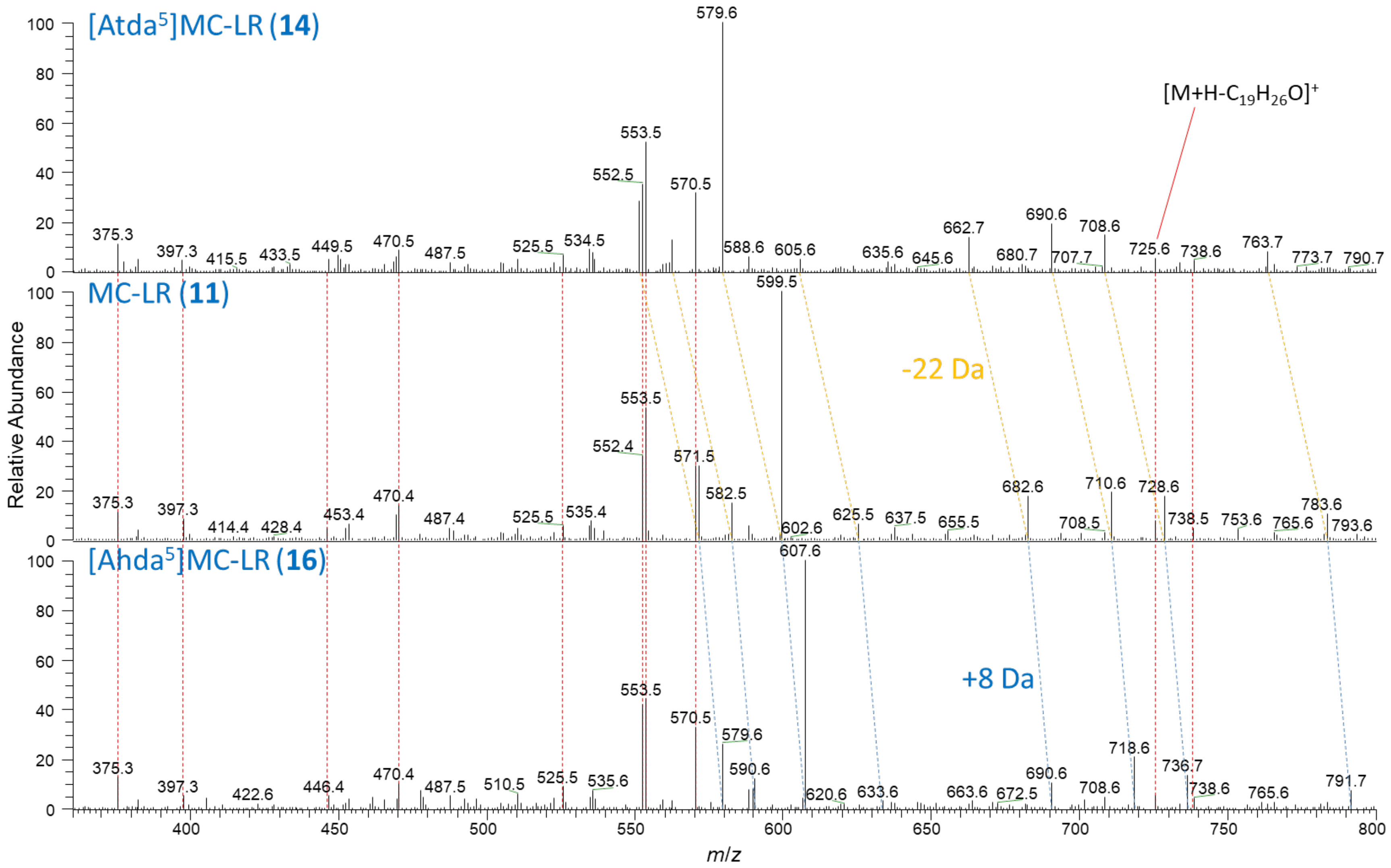

Figure S14. LC–MS^2 (method A) spectra of $[M + H]^+$ of: top, [Atda^5]MC-LR (**14**); middle, MC-LR (**11**), and; bottom, [Ahda^5]MC-LR (**16**), in positive ionization mode.

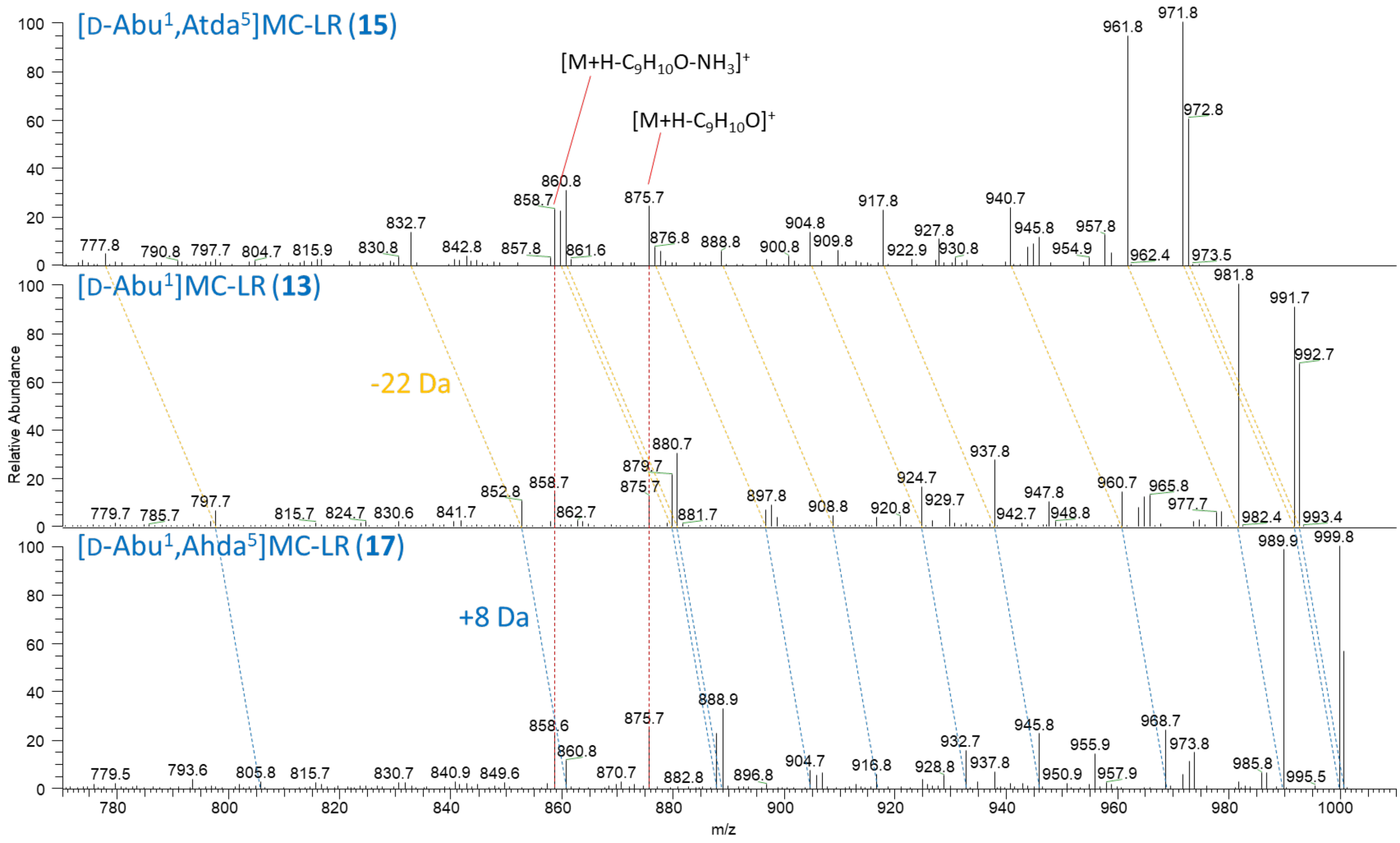

Figure S15. LC–MS^2 (method A) spectra of $[M + H]^+$ of: top, [D-Abu[1],Atda[5]]MC-LR (**15**); middle, [D-Abu[1]]MC-LR (**13**), and; bottom, [D-Abu[1],Ahda[5]]MC-LR (**17**), in positive ionization mode.

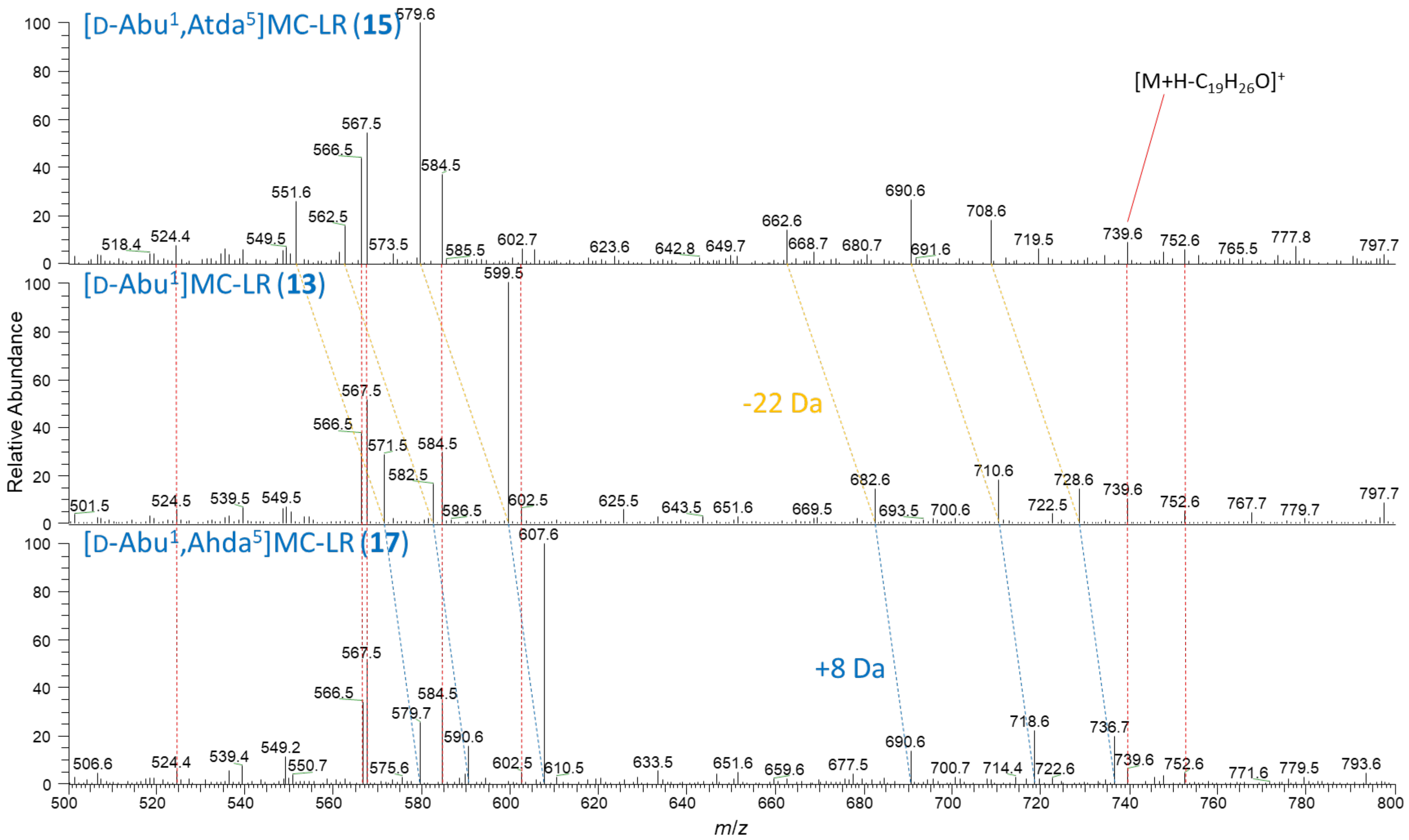

Figure S16. LC–MS[2] (method A) spectra of $[M + H]^+$ of: top, [D-Abu[1],Atda[5]]MC-LR (**15**); middle, [D-Abu[1]]MC-LR (**13**), and; bottom, [D-Abu[1],Ahda[5]]MC-LR (**17**), in positive ionization mode.

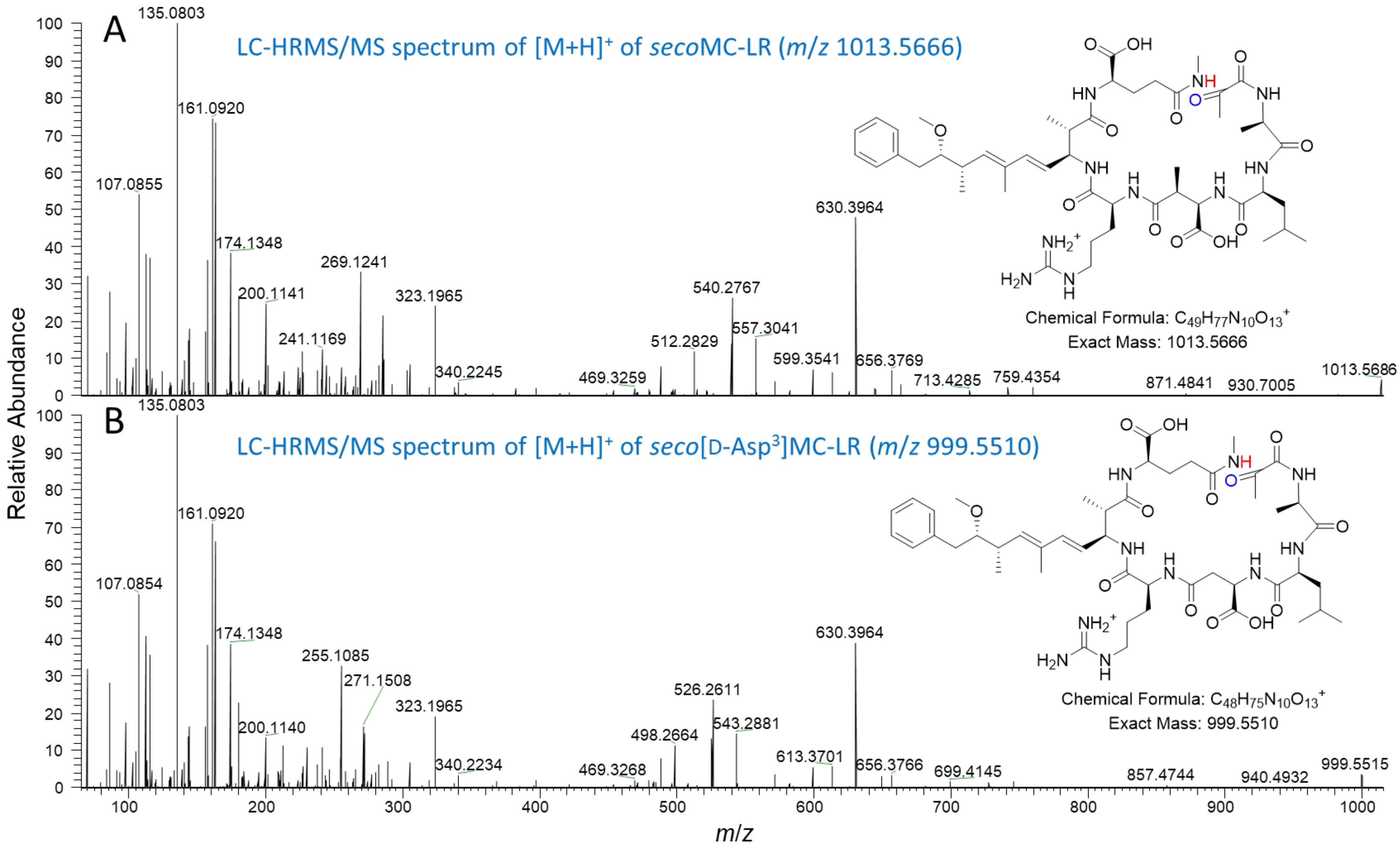

Figure S17. LC–HRMS/MS spectra of *seco*MC-LR and *seco*[D-Asp^3]MC-LR tentatively identified in the extract of *M. aeruginosa* PCC 7806 by comparison with the fragmentation pattern reported by Grach-Pogrebinsky et al. (*J. Nat. Prod*. **2004**, *67*, 337–342) for *seco*[D-Asp^3]MC-RR. In particular, characteristic product ions would be expected to include *m/z* 135, 143, 161, 163, 255, 323, 370/384, 526/540, 543/557, 630, and 745/759 for the compounds, based on the product ions reported for *seco*[D-Asp^3]MC-RR.

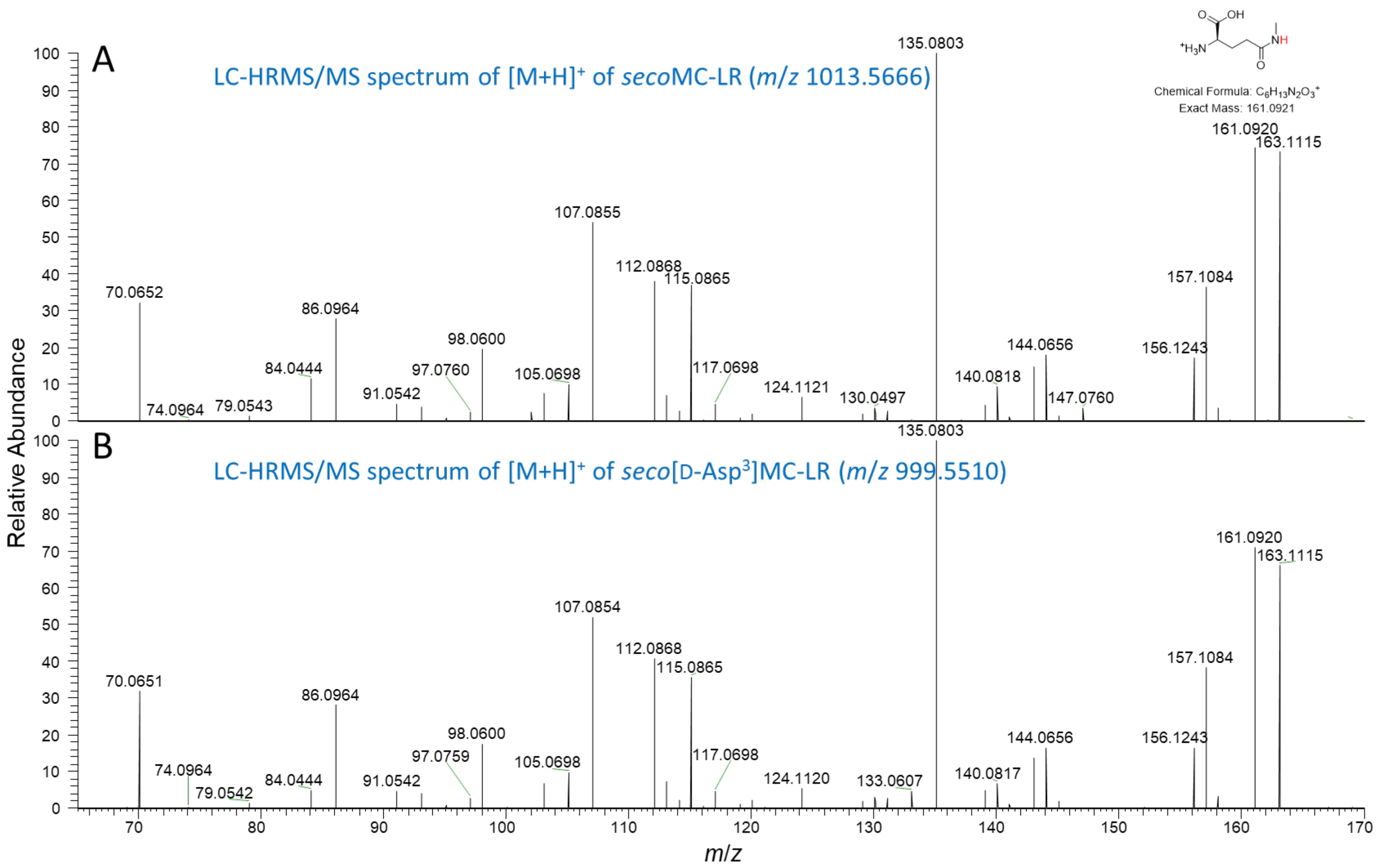

Figure S18. Expansion (*m*/*z* 65–170) of the LC–HRMS/MS spectra of: A, *seco*MC-LR, and; B, *seco*[D-Asp^3]MC-LR, from Figure S17. Note the prominent product ion at *m*/*z* 161.0920 (possible structure shown) and the absence of a product ion at *m*/*z* 155.0815 (cf. the spectrum of **11**, Figures S8 and S27–S30) arising from Mdha–D-Ala.

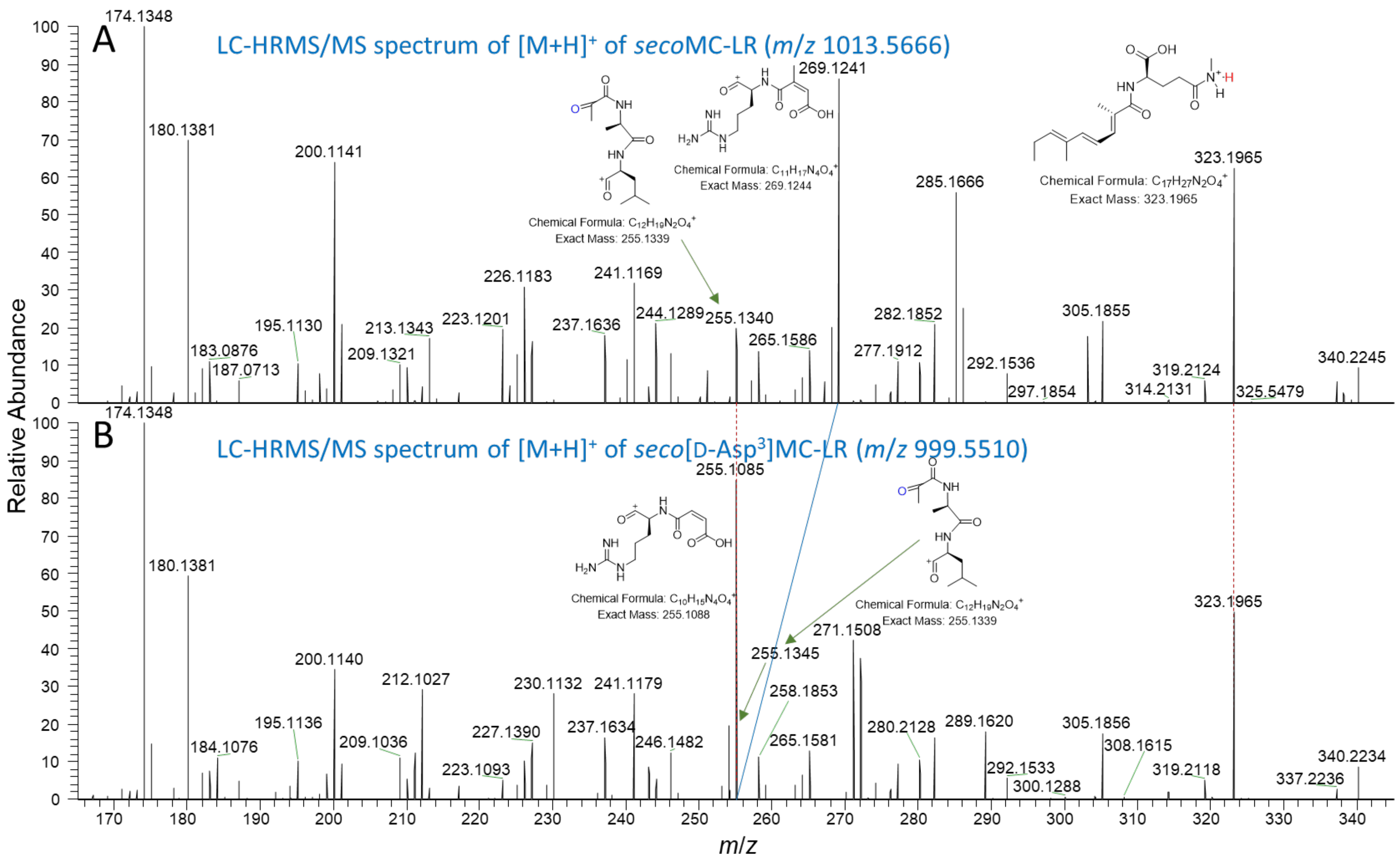

Figure S19. Expansion (*m*/*z* 165–345) of the LC–HRMS/MS spectra of: A, *seco*MC-LR, and; B, *seco*[D-Asp³]MC-LR, from Figure S17. Note the prominent product ion at *m*/*z* 323.1965 (possible structure shown, based on Grach-Pogrebinsky *et al*., 2004) and the absence of a product ion at *m*/*z* 213.0870 (cf. the spectrum of **11**, Figures S8 and S27–S30) arising from D-Glu–Mdha. The product ion at *m*/*z* 375.1914 arising from Adda–D-Glu–Mdha – $C_9H_{10}O$ was also absent, having been replaced by *m*/*z* 323.1965.

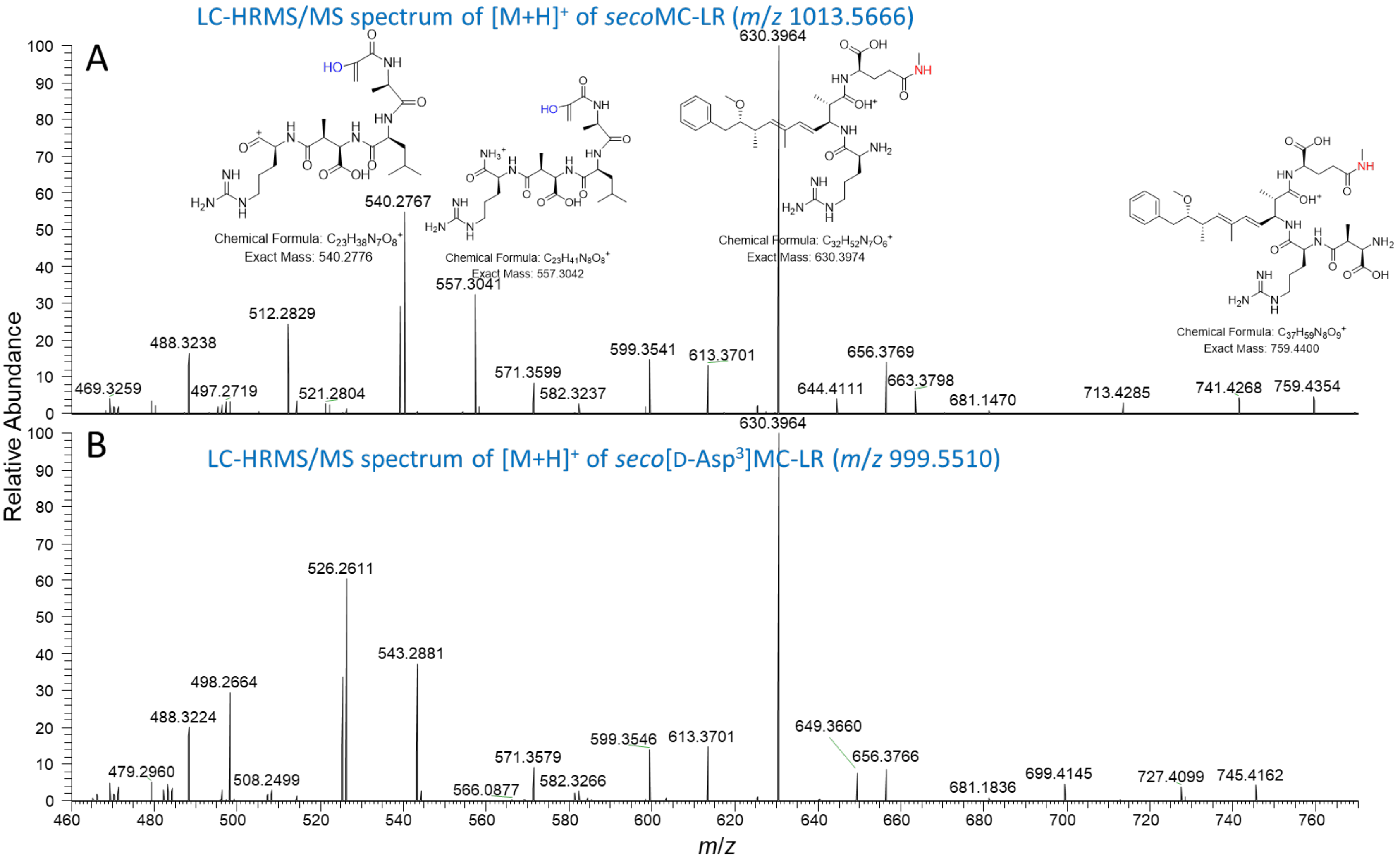

Figure S20. Expansion (*m*/*z* 460–770) of the LC–HRMS/MS spectra of: A, *seco*MC-LR, and; B, *seco*[D-Asp[3]]MC-LR, from Figure S17. Note the prominent product ions at *m*/*z* 540.2767, 557.3041, and 630.3074 (possible structures shown, based on Grach-Pogrebinsky *et al*., 2004) in the HRMS/MS of *seco*MC-LR (cf. the spectrum of **11**, Figures S8 and S27–S30).

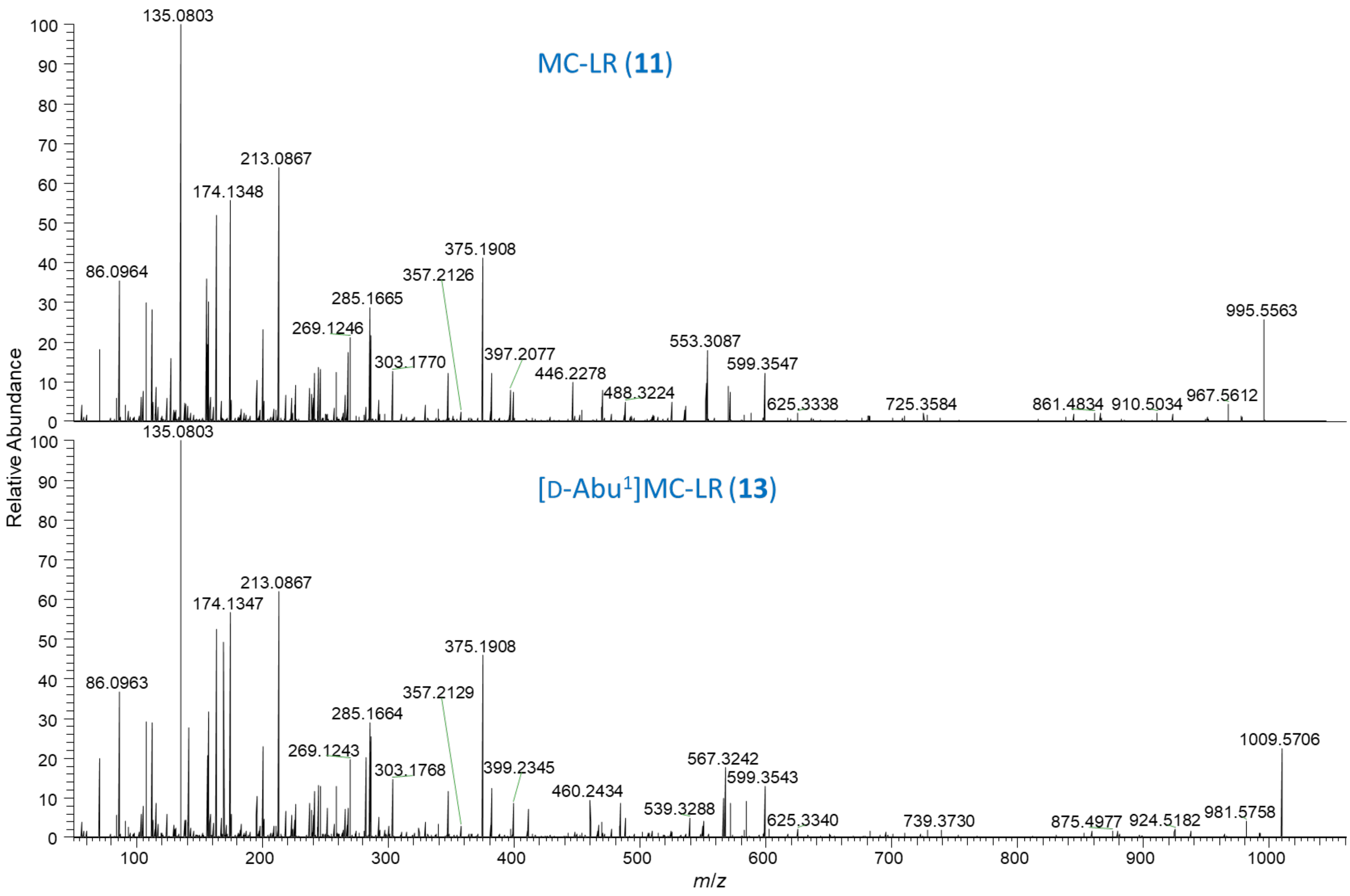

Figure S21. LC–HRMS/MS (method B) spectra of $[M + H]^+$ of MC-LR (**11**) and [D-Abu¹]MC-LR (**13**) in positive ionization mode. For expansions, see Figures 3 and S18.

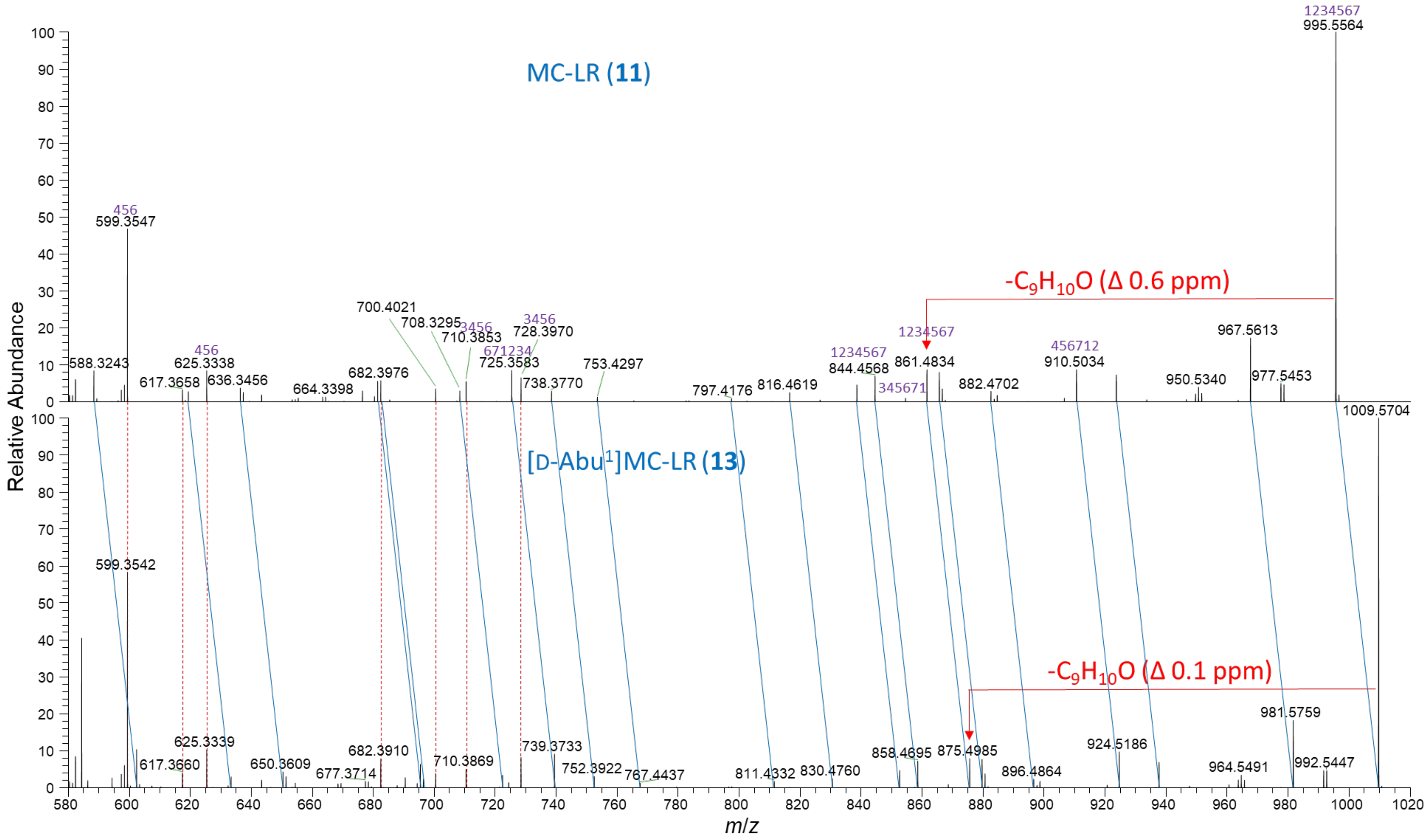

Figure S22. Expansion (*m*/*z* 580–1020) of LC–HRMS/MS (method B) spectra of $[M + H]^+$ of MC-LR (**11**) and [D-Abu[1]]MC-LR (**13**) from Figure S17 (for spectra from *m*/*z* 70–610, see Figure 3). Purple numbers indicate the amino acids attributable to the ion. Blue lines connect selected ions differing by 14.0157 Da (CH_2).

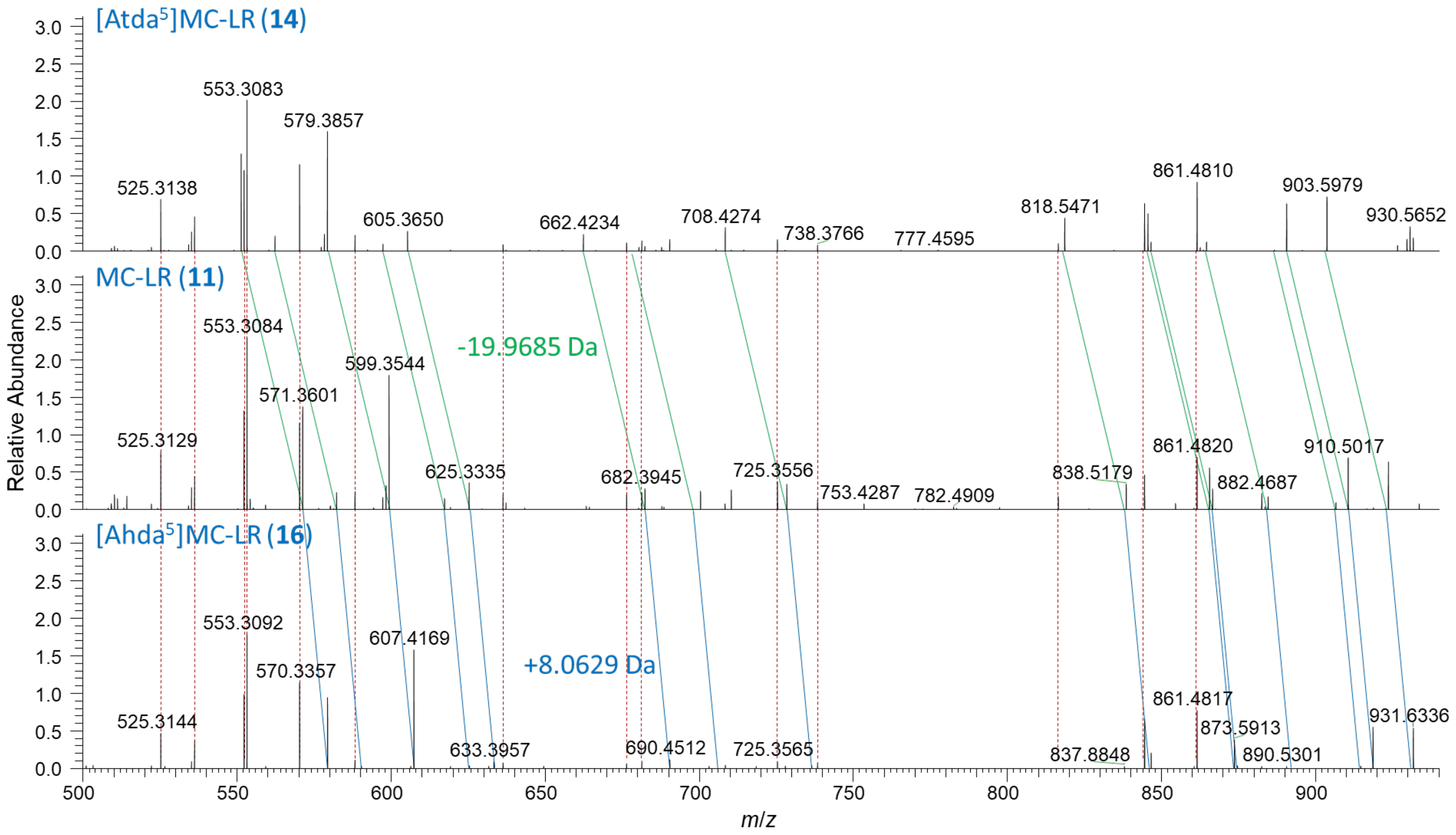

Figure S23. LC–HRMS/MS (method B) spectra of $[M + H]^+$ of: top, [Atda⁵]MC-LR (**14**); middle, MC-LR (**11**), and; bottom, [Ahda⁵]MC-LR (**16**), in positive ionization mode. Blue and green lines connect selected ions differing by 8.0629 and 19.9685 Da, respectively, from those of MC-LR (**11**).

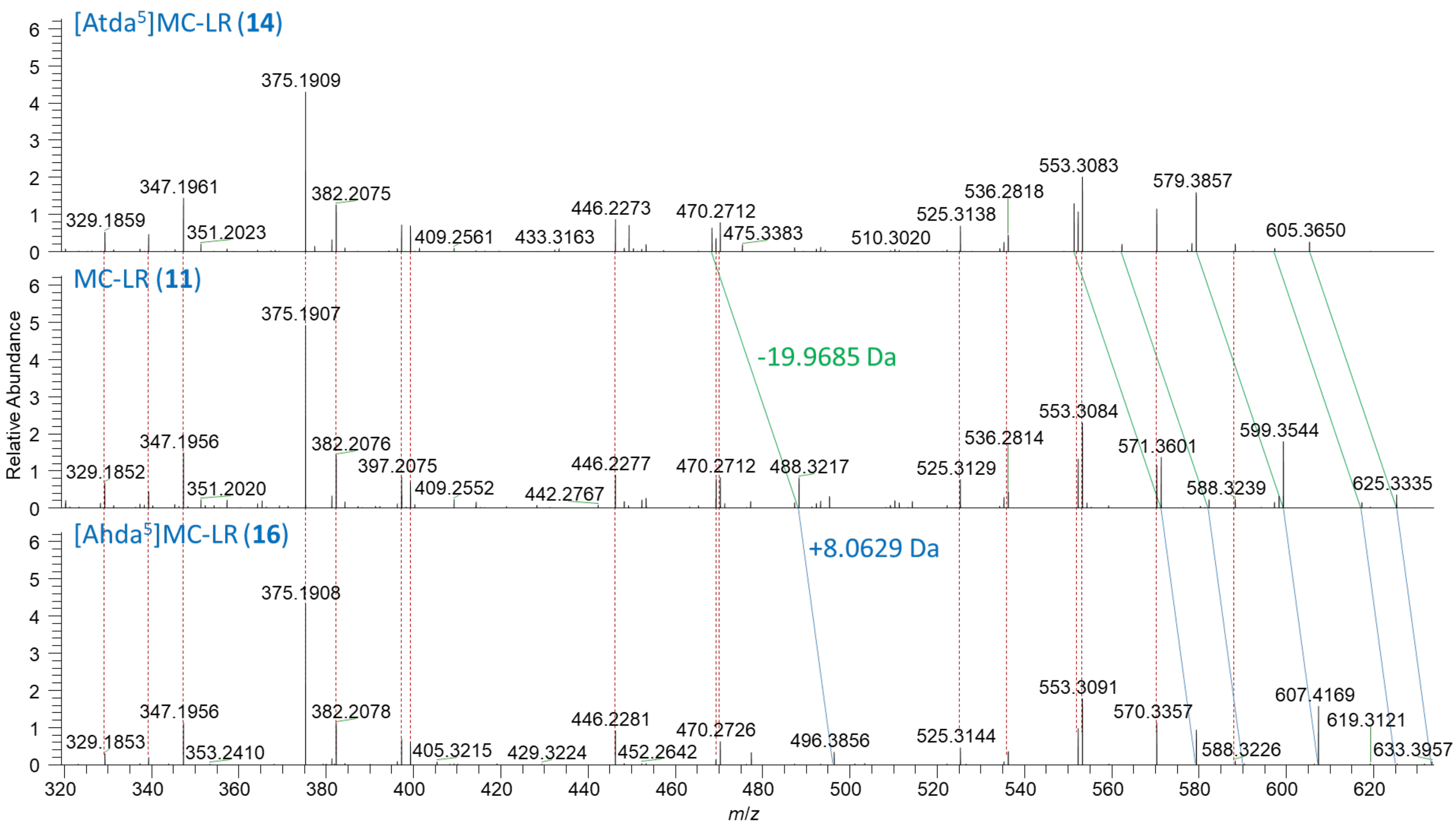

Figure S24. LC–HRMS/MS (method B) spectra of $[M + H]^+$ of: top, [Atda^5]MC-LR (**14**); middle, MC-LR (**11**), and; bottom, [Ahda^5]MC-LR (**16**), in positive ionization mode. Blue and green lines connect selected ions differing by 8.0629 and 19.9685 Da, respectively, from those of MC-LR (**11**).

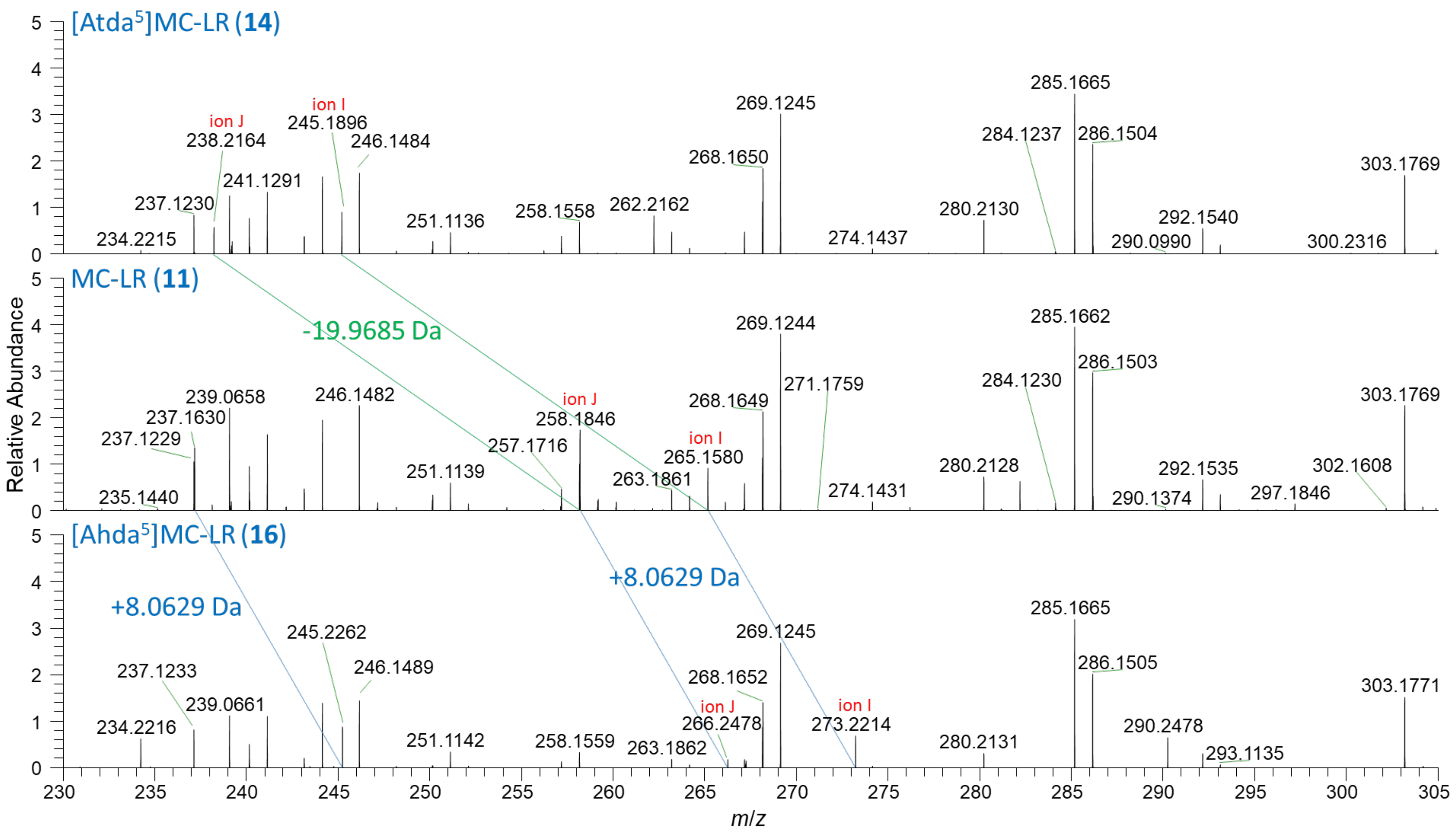

Figure S25. LC–HRMS/MS (method B) spectra of $[M + H]^+$ of: top, [Atda^5]MC-LR (**14**); middle, MC-LR (**11**), and; bottom, [Ahda^5]MC-LR (**16**), in positive ionization mode. Blue and green lines connect selected ions differing by 8.0629 and 19.9685 Da, respectively, from those of MC-LR (**11**).

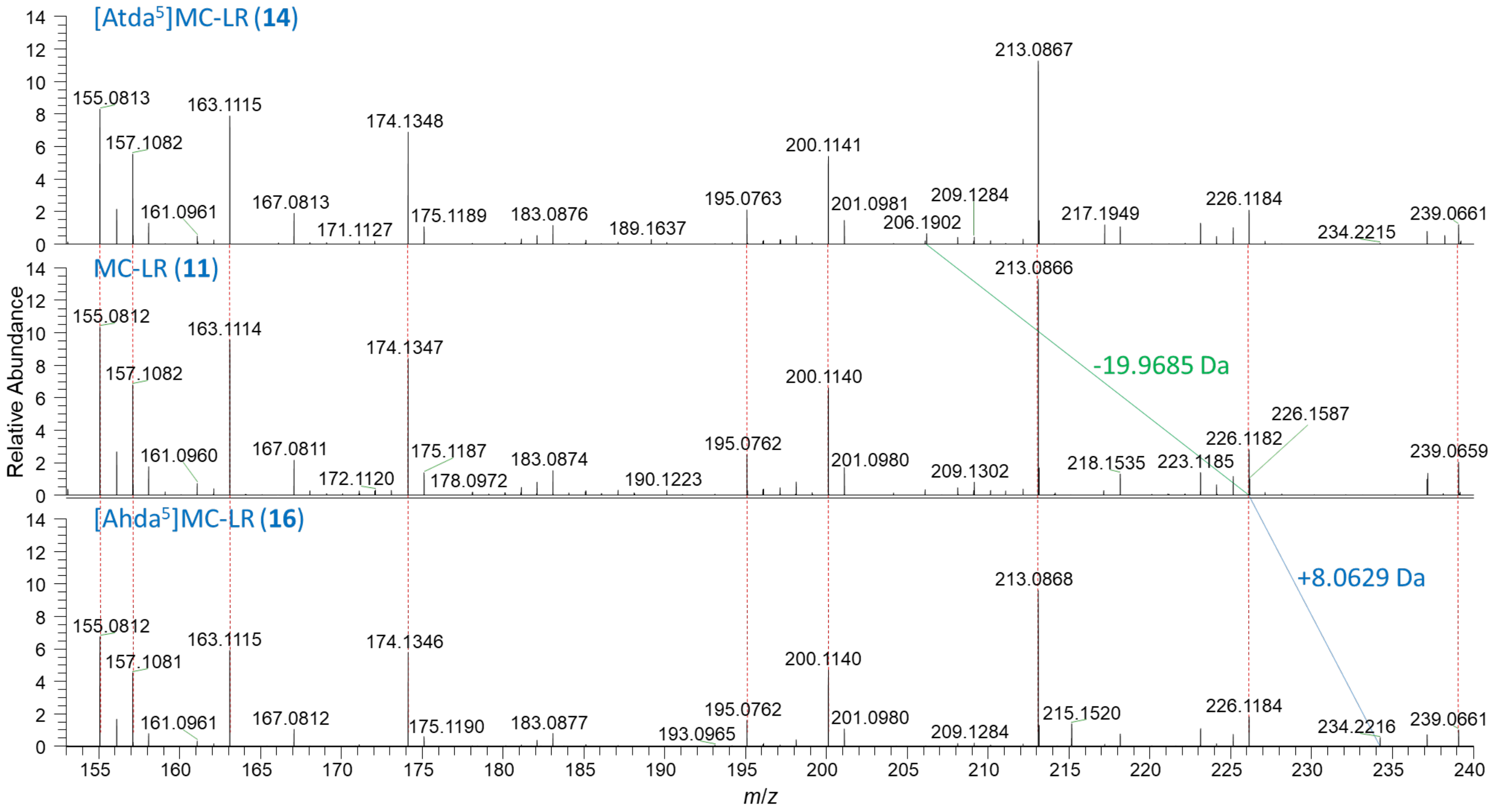

Figure S26. LC–HRMS/MS (method B) spectra of $[M + H]^+$ of: top, [Atda^5]MC-LR (**14**); middle, MC-LR (**11**), and; bottom, [Ahda^5]MC-LR (**16**), in positive ionization mode. Blue and green lines connect selected ions differing by 8.0629 and 19.9685 Da, respectively, from those of MC-LR (**11**).

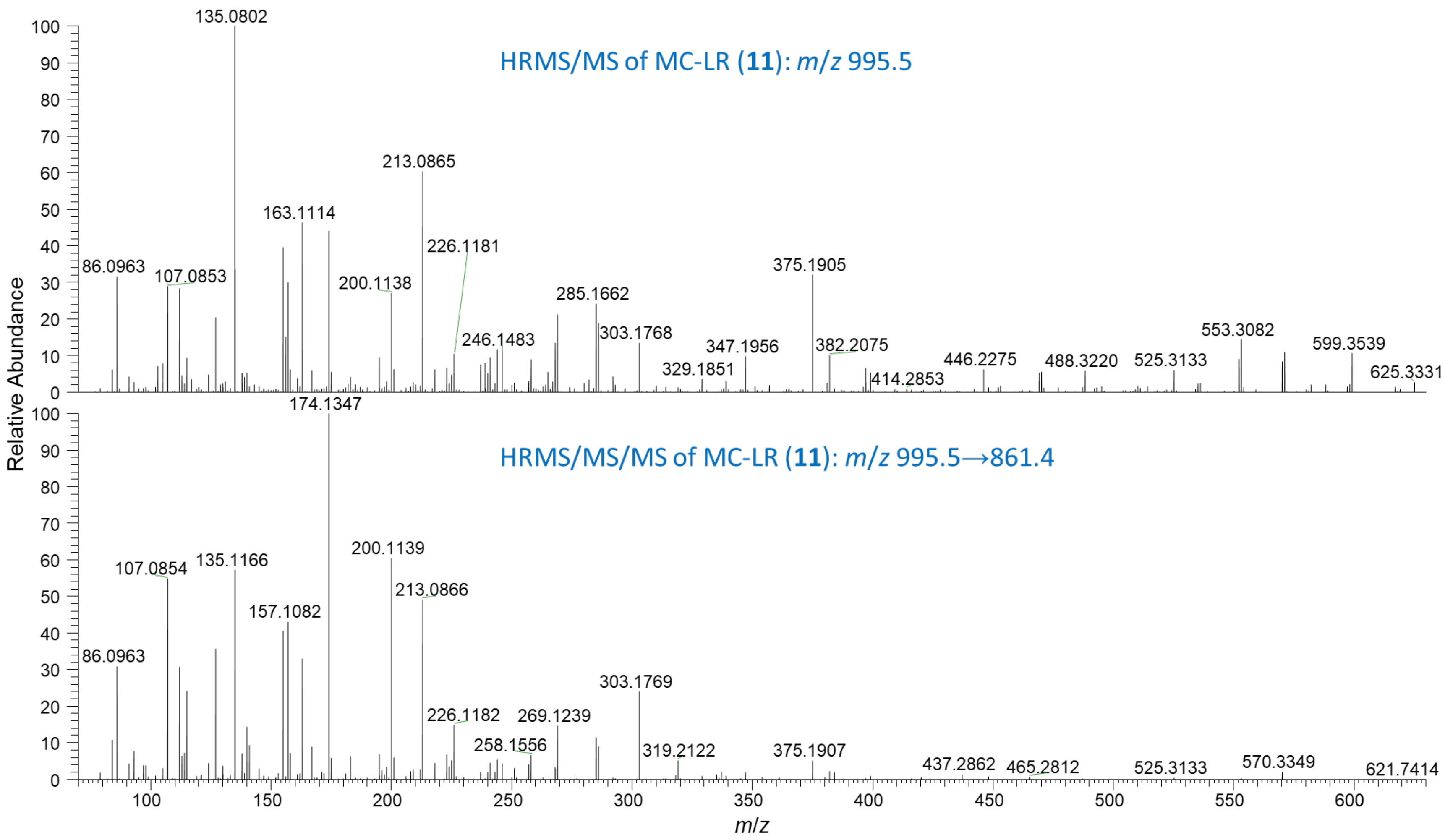

Figure S27. LC–HRMS/MS/MS (method B) spectra in positive ionization mode of: top, $[M + H]^+$ of MC-LR (**11**), and; bottom, $[M + H – C_9H_{10}O]^+$ at *m*/*z* 861.4 produced from **11** by in-source fragmentation.

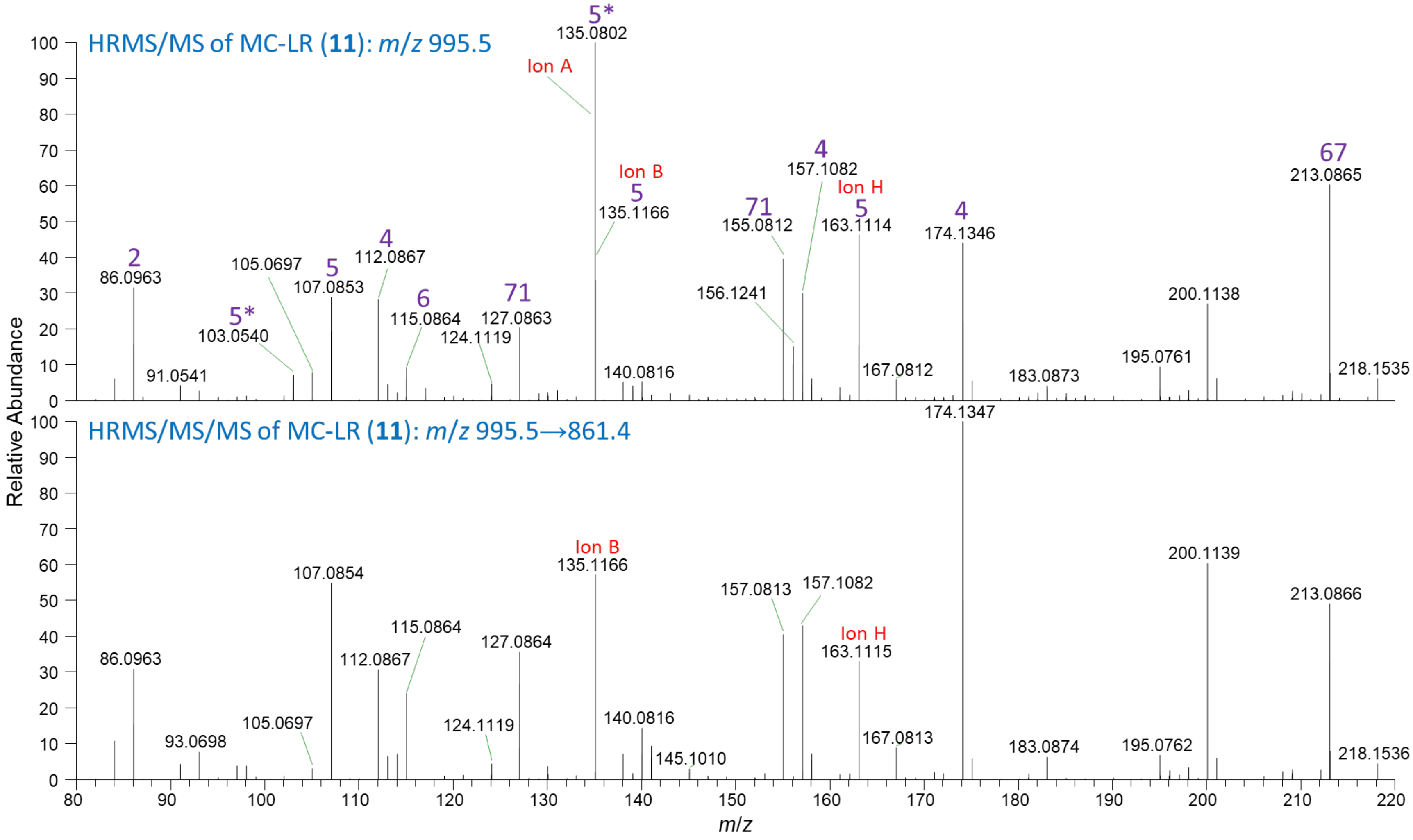

Figure S28. LC–HRMS/MS/MS (method B) spectra of: top, $[M + H]^+$ of MC-LR (**11**), and; bottom, $[M + H - C_9H_{10}O]^+$ at *m*/*z* 861.4 produced from **11** by in-source fragmentation, showing an expansion of the *m*/*z* 80–220 region of the spectra in Figure S23. Purple numbers indicate the amino acids from which selected product ions are derived, for ions A, B, and H see Figures 1 and 6.

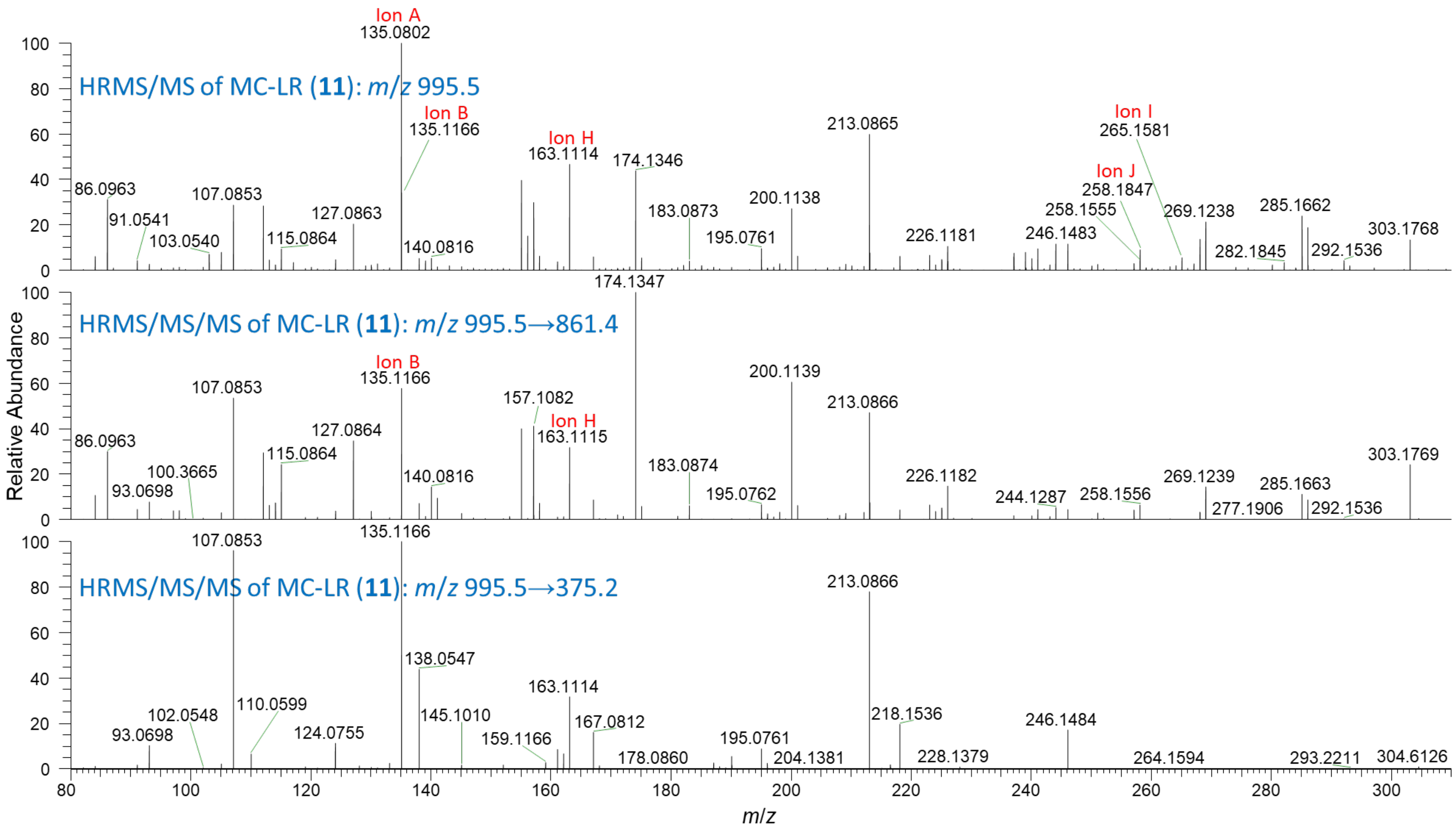

Figure S29. LC–HRMS/MS/MS (method B) spectra (*m/z* 80–320) of: top, $[M + H]^+$ of MC-LR (**11**); middle, $[M + H – C_9H_{10}O]^+$ at *m/z* 861.4 produced from **11** by in-source fragmentation, and; bottom, *m/z* 375.2 (ion C, see Figure 1) produced from **11** by in-source fragmentation. Note the absence of ions A, I, and J (*m/z* 135.0804, 265.1587, and 258.1852, respectively) after the in-source fragmentation (middle and bottom spectra).

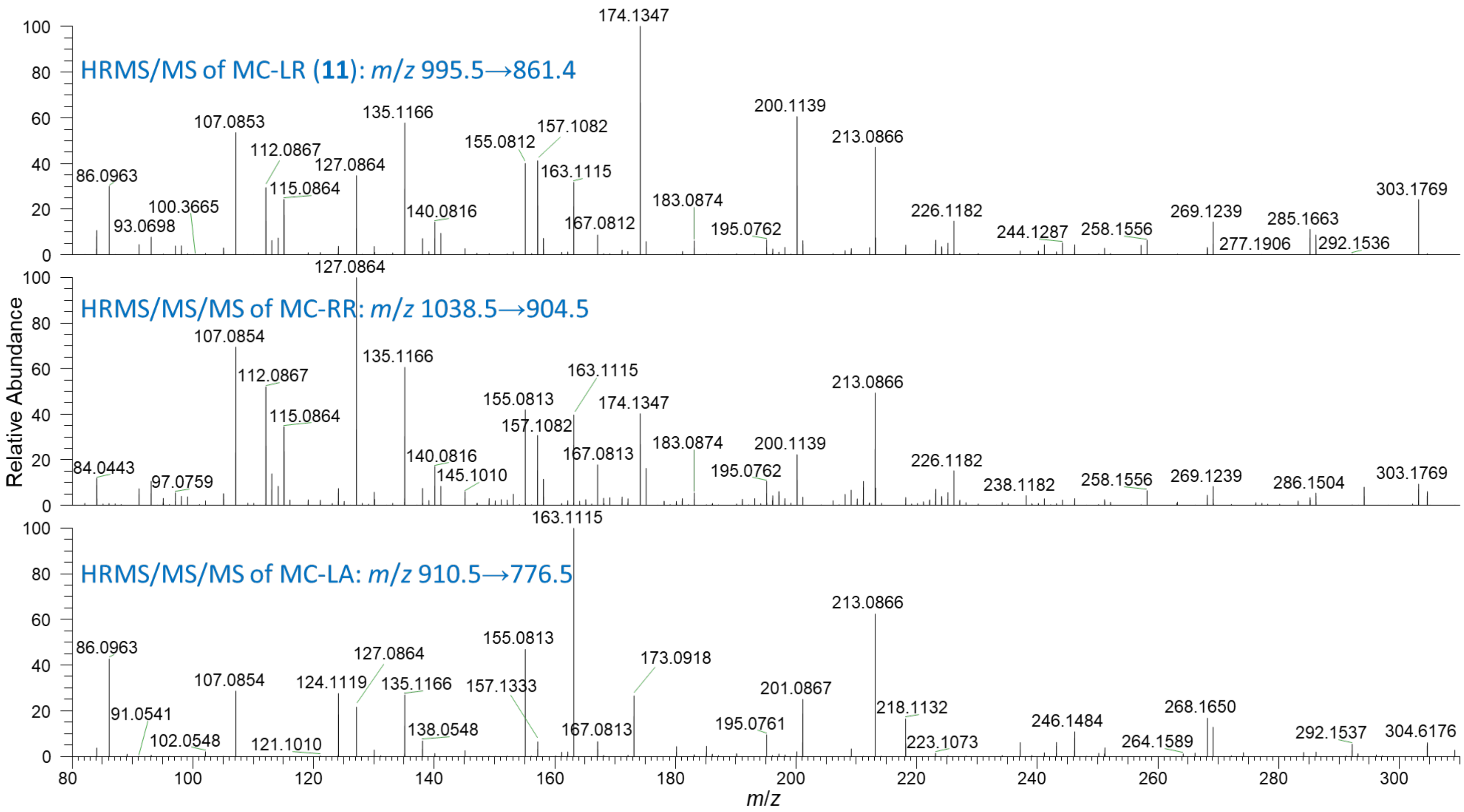

Figure S30. LC–HRMS/MS (method B) spectra of $[M + H - C_9H_{10}O]^+$ produced by in-source fragmentation of: top, MC-LR (**11**), at *m*/*z* 861.4; middle, MC-RR, at *m*/*z* 904.5, and; bottom, MC-LA, at *m*/*z* 776.5. Note the absence of ions A, I, and J (*m*/*z* 135.0804, 265.1587, and 258.1852, respectively) in all spectra after in-source fragmentation.

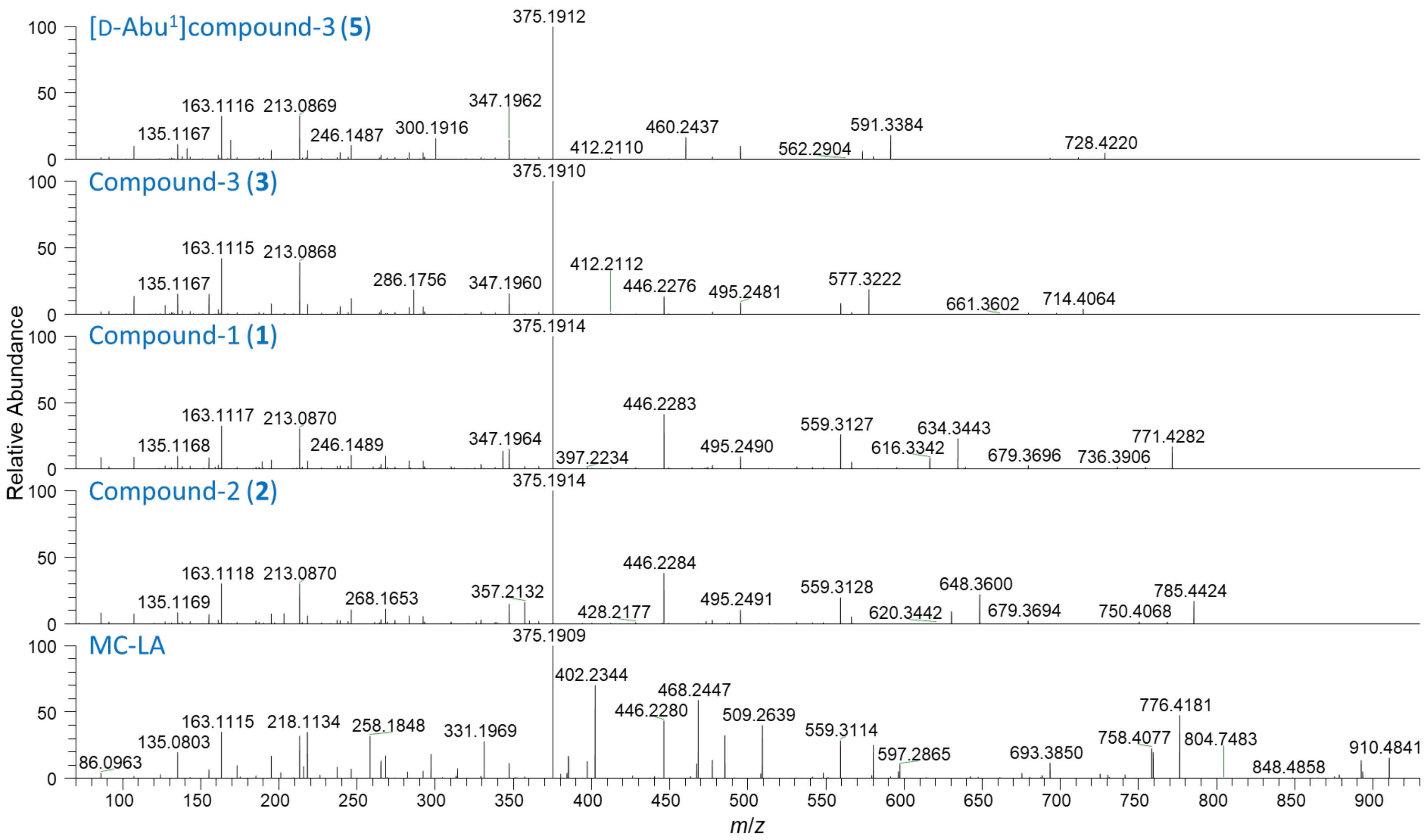

Figure S31. LC–HRMS/MS (method B) spectra of $[M + H]^+$ of the truncated DMAdda^5-microcystin analogues **1**–**3**, **5**, and MC-LA, (*m*/*z* 70–930).

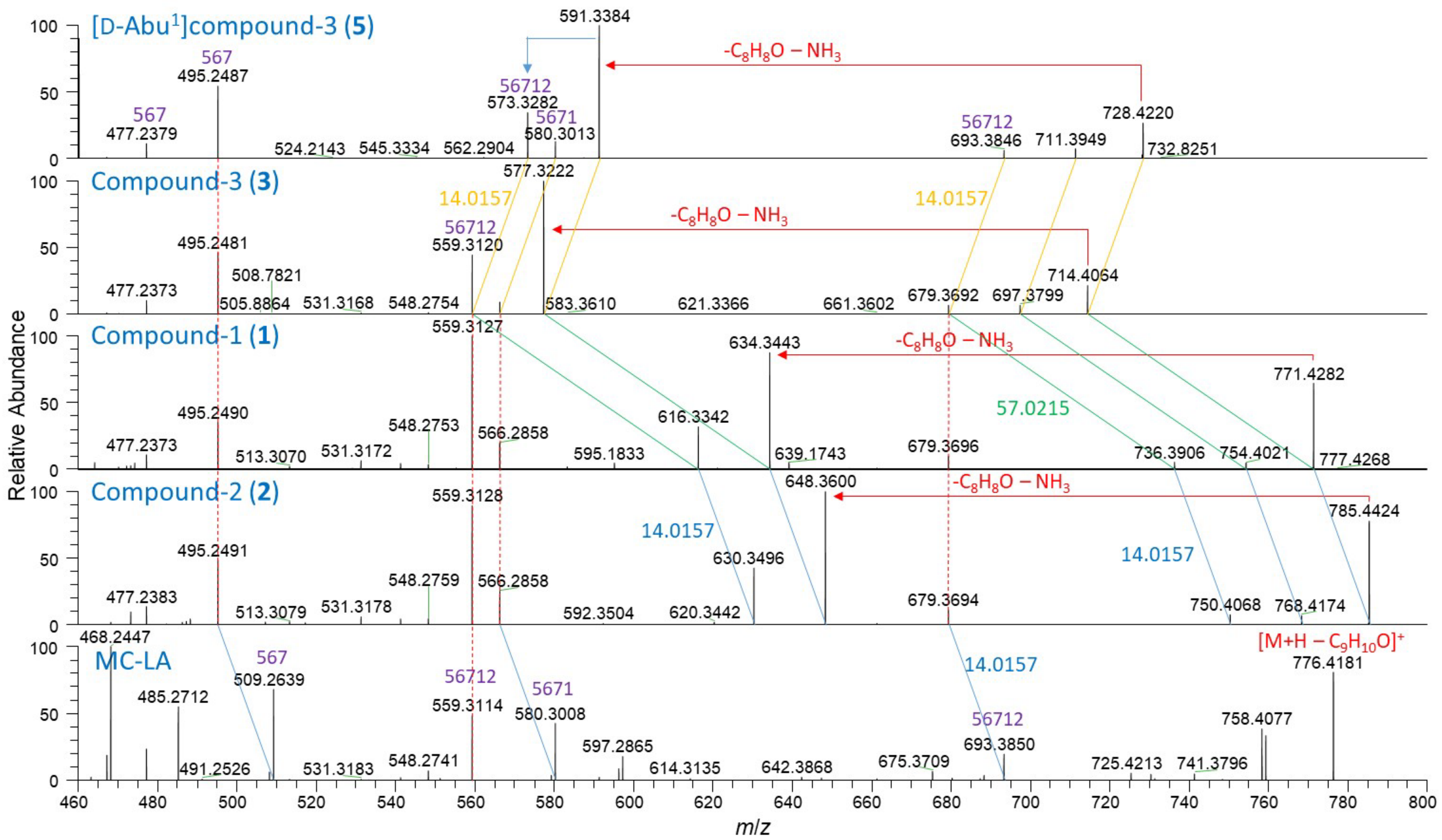

Figure S32. LC–HRMS/MS (method B) spectra of $[M + H]^+$ of the truncated DMAdda^5-microcystin analogues **1**–**3**, **5**, and MC-LA (*m*/*z* 500–800 from Figure S27). Note the neutral loss of C_8H_8O + NH_3 (137.0835 Da) from the DMAdda^5 moieties of the precursor ions of **1**–**3** and **5**, and the presence of many shared product ions arising from the Adda^5–D-Glu^6–Mdha^7–D-Ala^1–Leu^2 portion of MC-LA. Amino acid residues attributable to selected product ions are numbered in purple, and ions linked by blue and orange lines differ by 14.0157 Da (CH_2) while those linked by green lines differ by 57.0215 Da (C_2H_3ON; Gly).

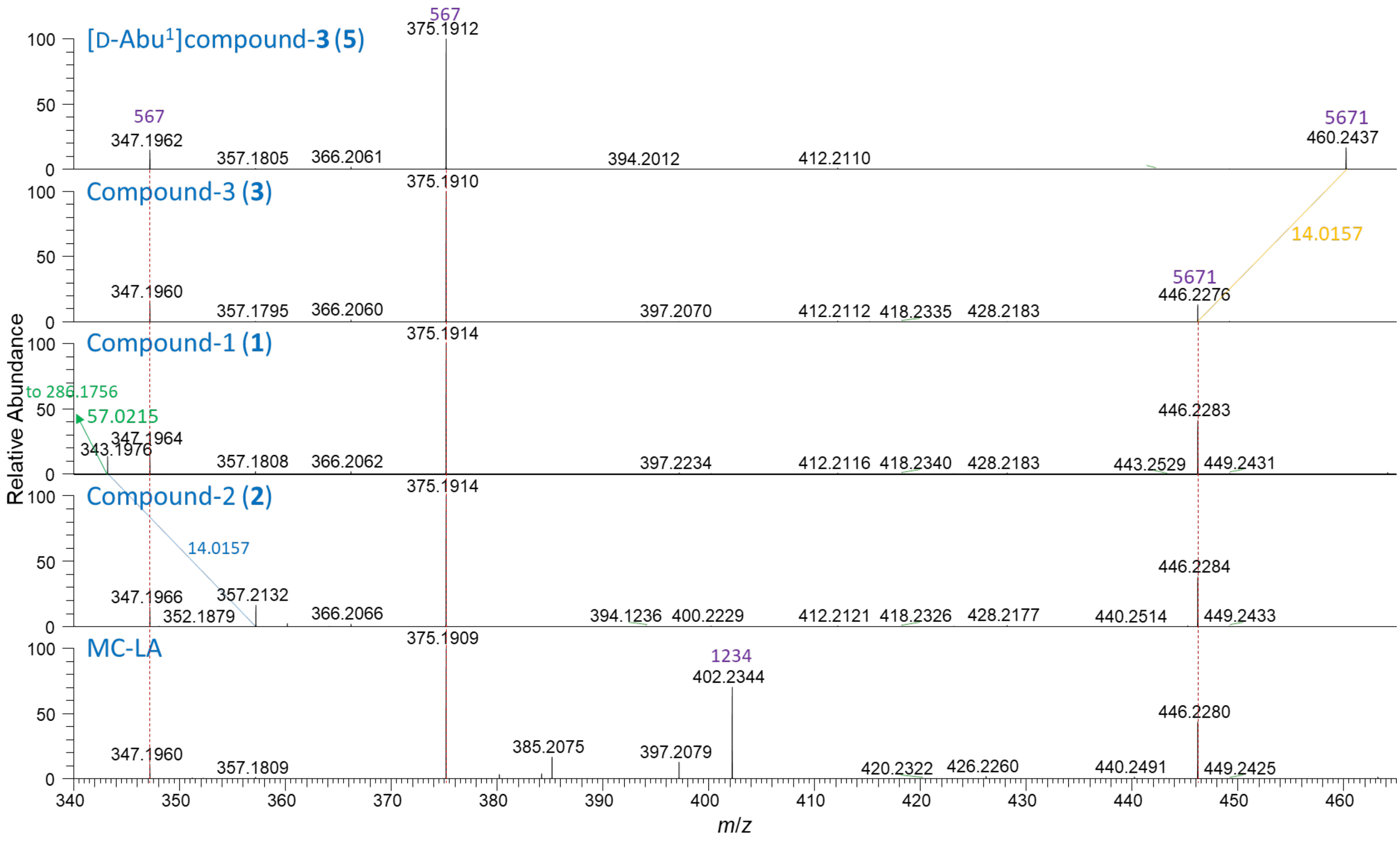

Figure S33. LC–HRMS/MS (method B) spectra of $[M + H]^+$ of the truncated DMAdda[5]-microcystin analogues **1**–**3**, **5**, and MC-LA (*m*/*z* 340–465 from Figure S27). Note the presence of many shared product ions arising from the Adda[5]–D-Glu[6]–Mdha[7]–D-Ala[1]–Leu[2] portion of MC-LA. Amino acid residues attributable to selected product ions are numbered in purple, and ions linked by blue and orange lines differ by 14.0157 Da (CH_2) while those linked by green lines differ by 57.0215 Da (C_2H_3ON; Gly).

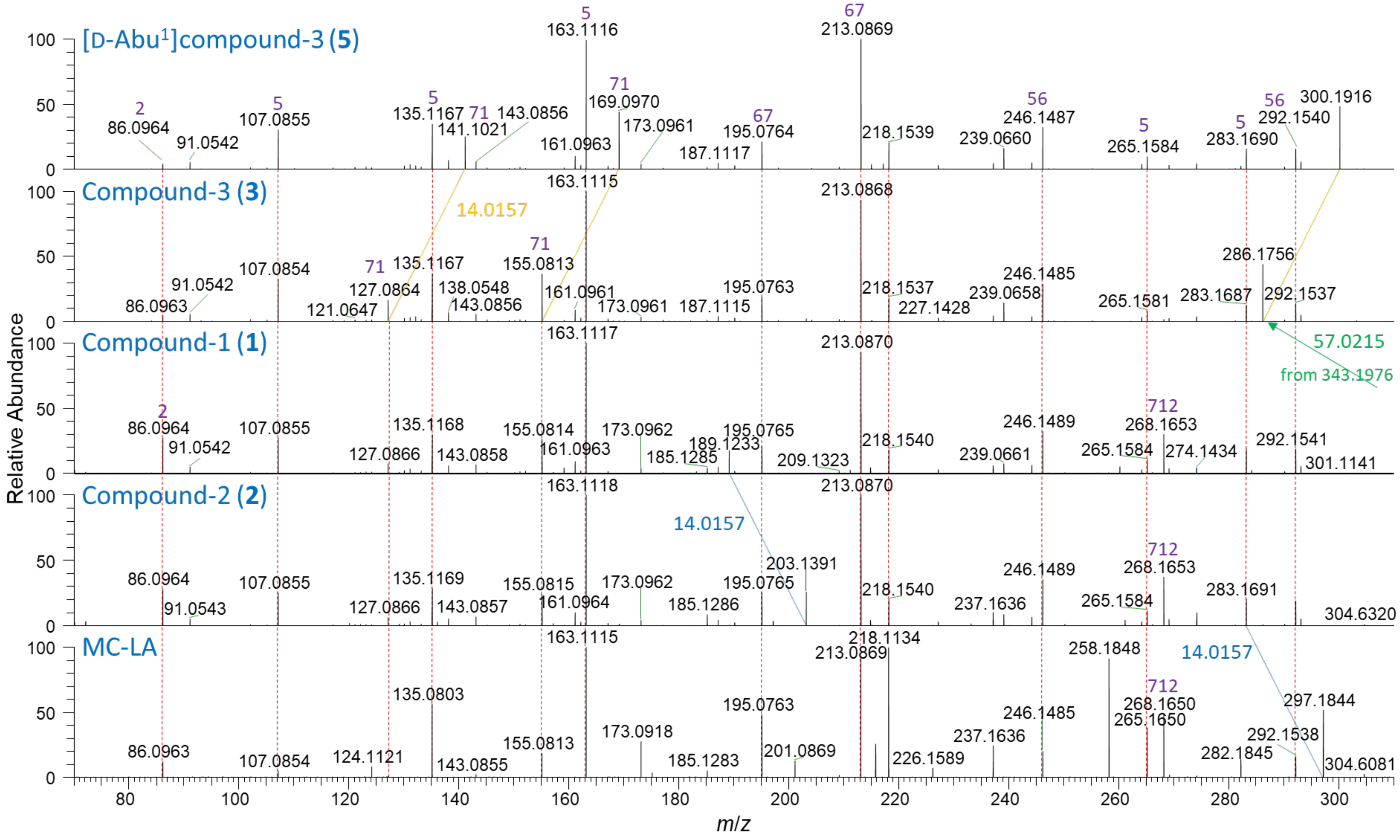

Figure S34. LC–HRMS/MS (method B) spectra of $[M + H]^+$ of the truncated DMAdda⁵-microcystin analogues **1**–**3**, **5**, and MC-LA (*m*/*z* 70–310 from Figure S27). Note the presence of many shared product ions arising from the $Adda^5$–D-Glu^6–$Mdha^7$–D-Ala^1–Leu^2 portion of MC-LA. Amino acid residues attributable to selected product ions are numbered in purple, and ions linked by blue and orange lines differ by 14.0157 Da (CH_2) while those linked by green lines differ by 57.0215 Da (C_2H_3ON; Gly).

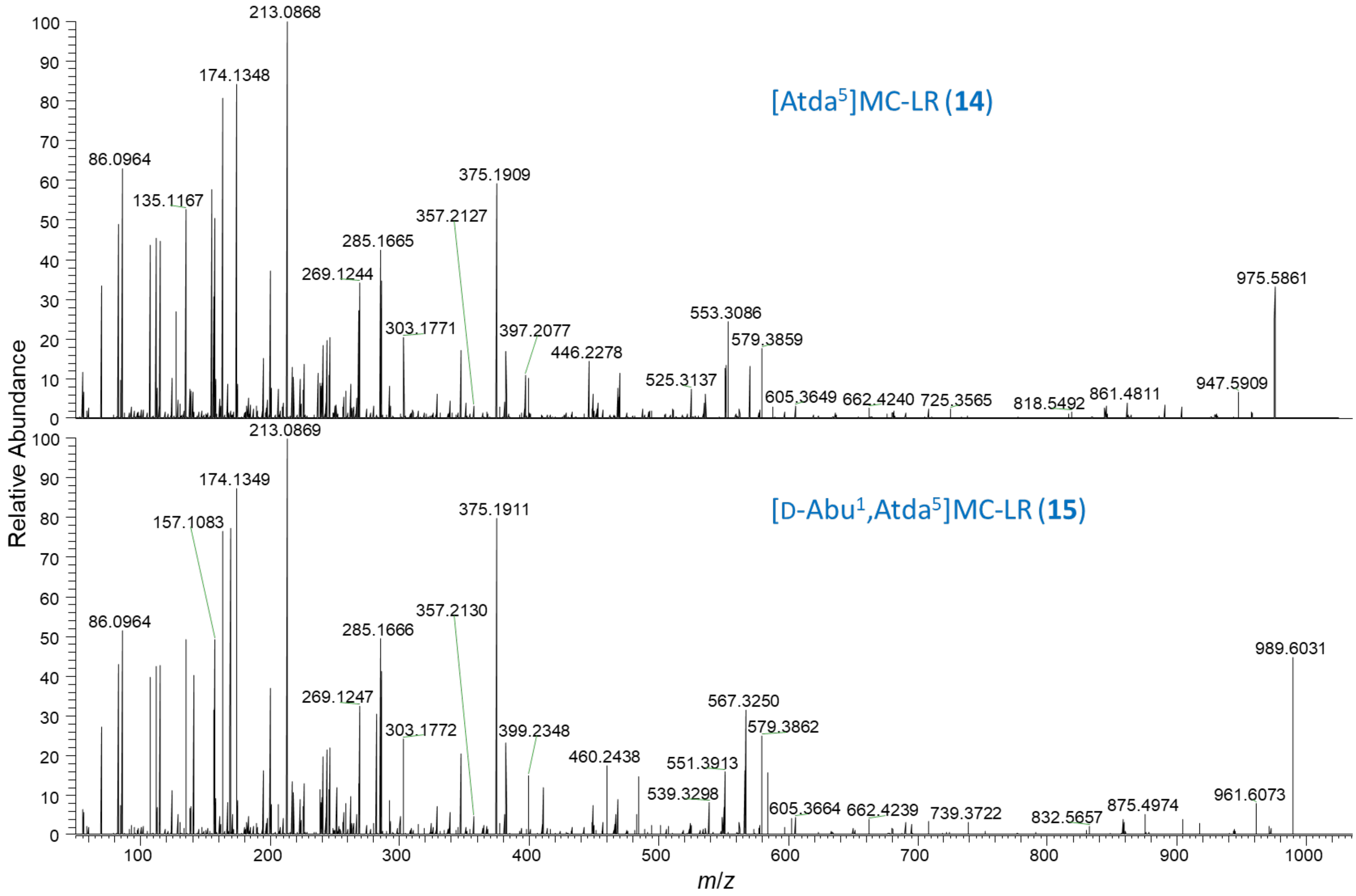

Figure S35. LC–HRMS/MS (method B) spectra of $[M + H]^+$ of [Atda^5]MC-LR (**14**) and [D-Abu^1,Atda^5]MC-LR (**15**).

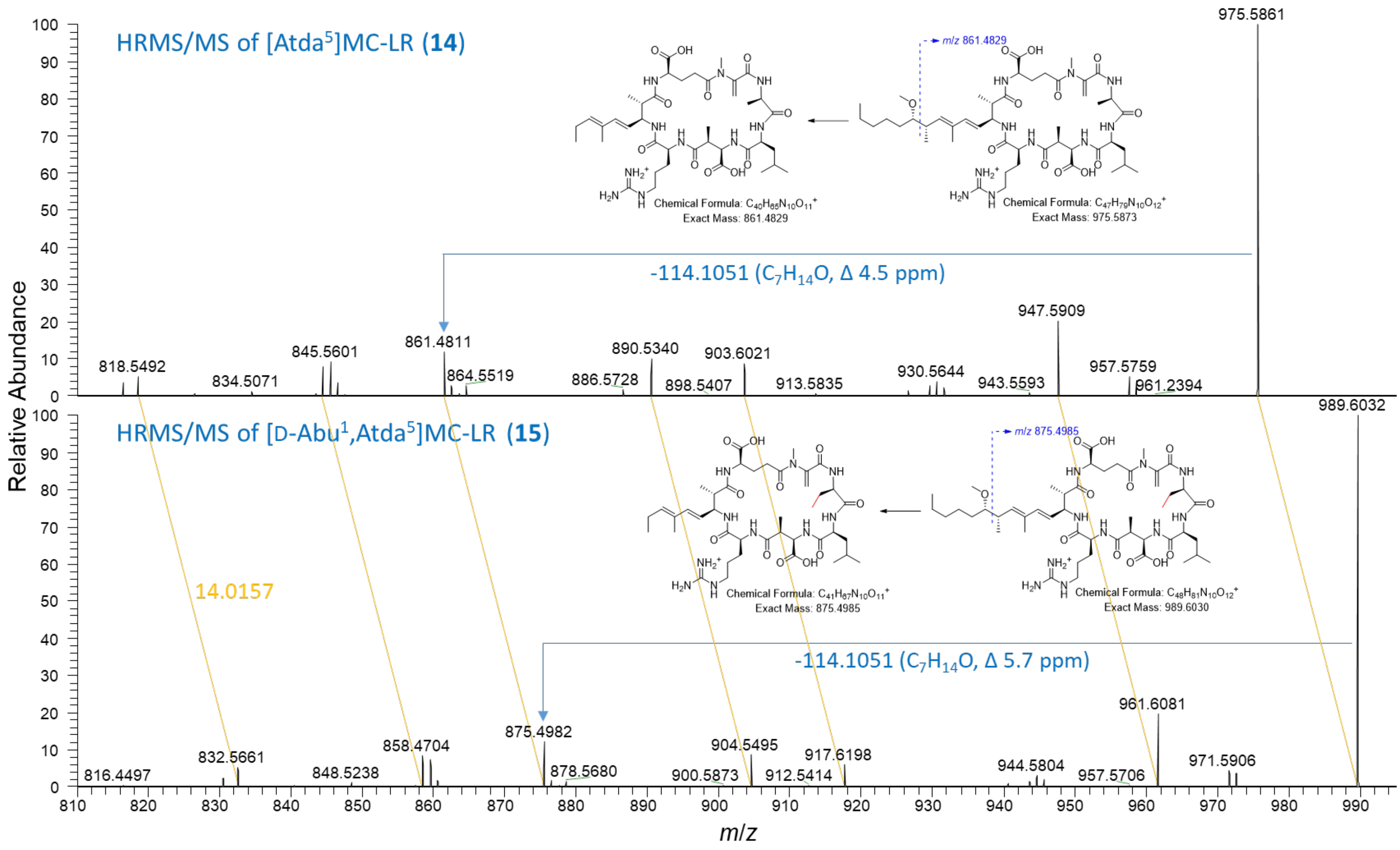

Figure S36. LC–HRMS/MS (method B) spectra of $[M + H]^+$ of [Atda5]MC-LR (**14**) and [D-Abu1,Atda5]MC-LR (**15**), showing an expansion (*m*/*z* 810–995) from Figure S35. Note the neutral loss of the Adda terminus (114.1045 Da). Ions connected with orange lines differ by 14.0157 Da (CH_2), and are attributable to product ions containing the amino acid at position-1.

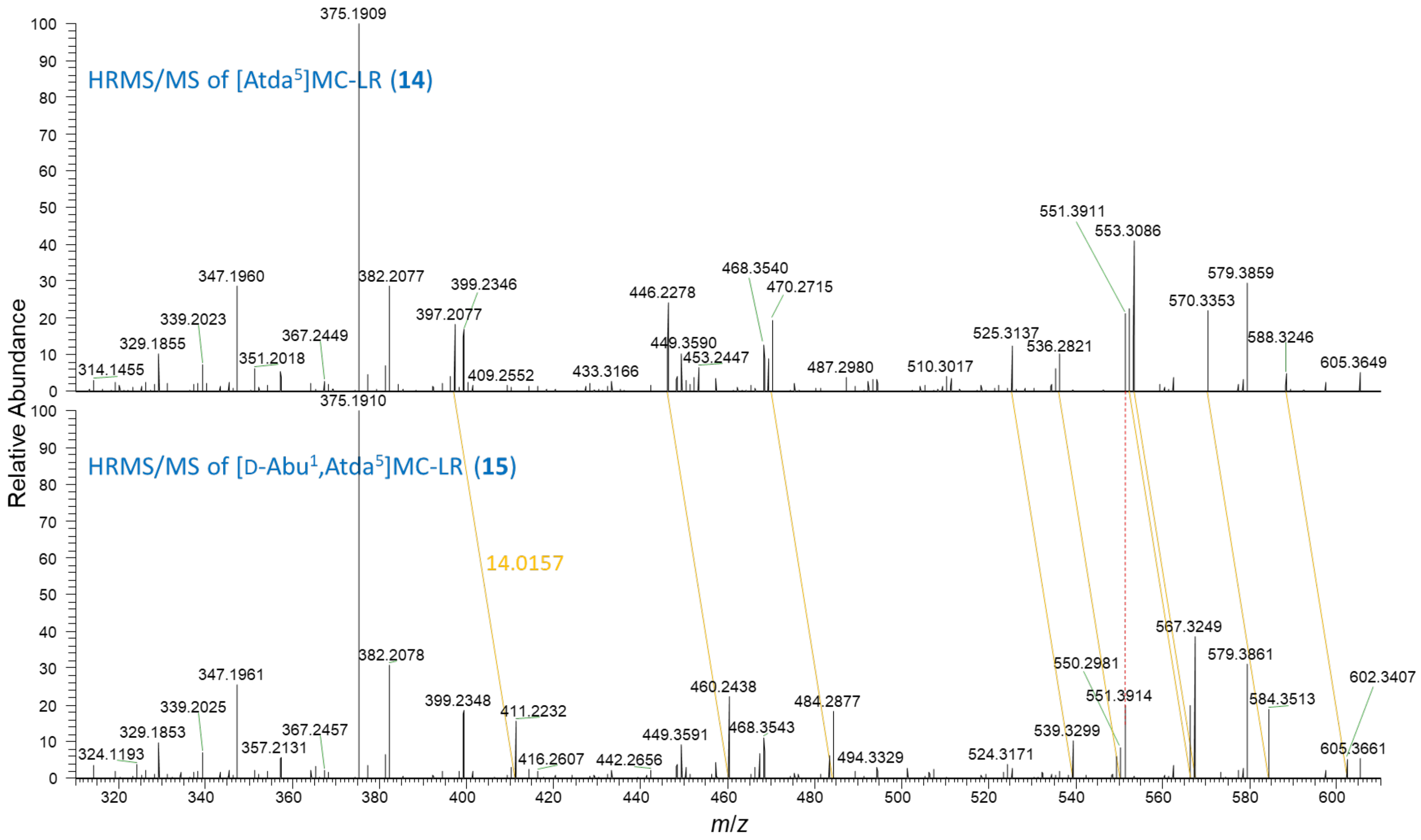

Figure S37. LC–HRMS/MS (method B) spectra of $[M + H]^+$ of [Atda⁵]MC-LR (**14**) and [D-Abu¹,Atda⁵]MC-LR (**15**), showing an expansion (*m*/*z* 310–610) from Figure S35. Ions connected with orange lines differ by 14.0157 Da (CH_2), and are attributable to product ions containing the amino acid at position-1.

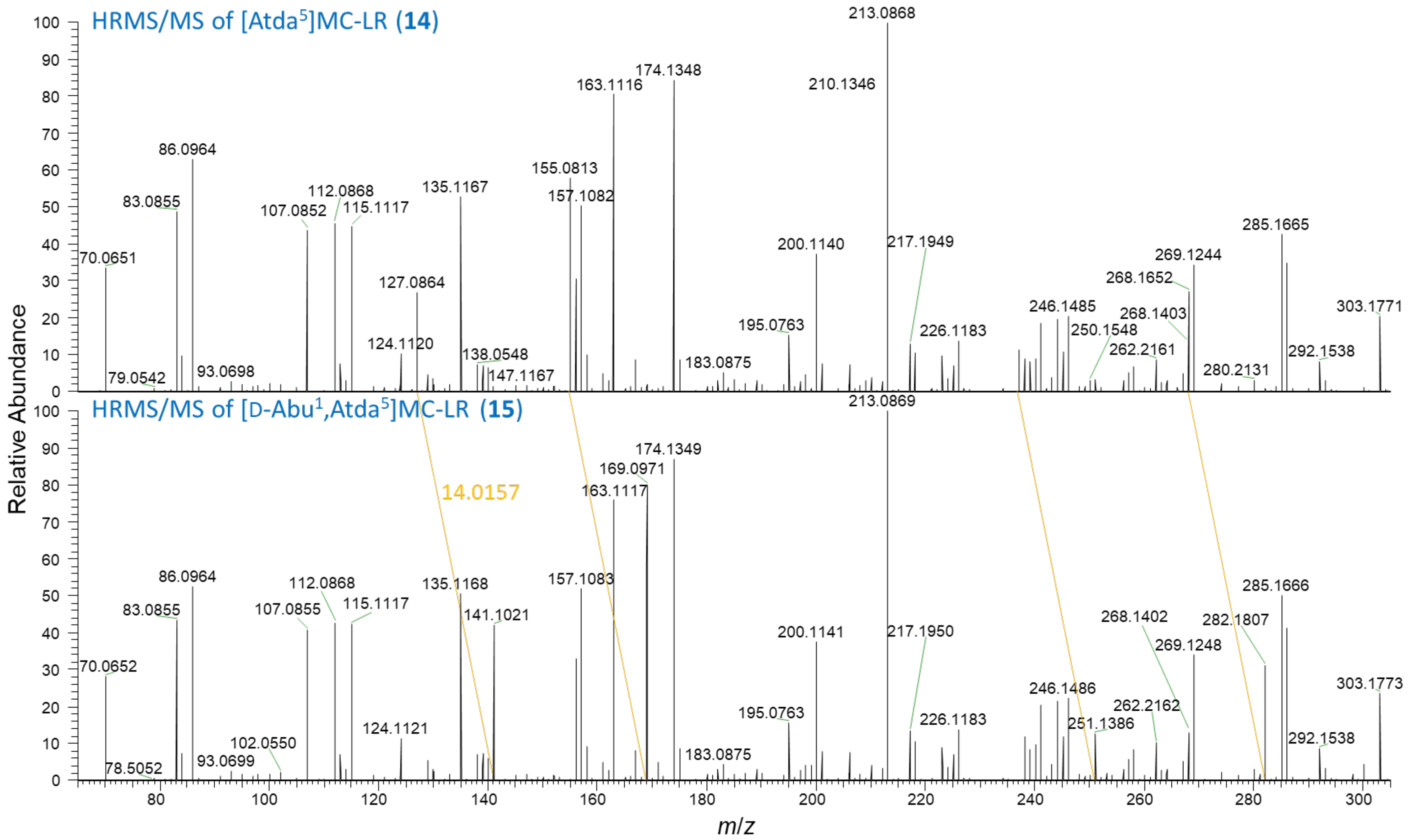

Figure S38. LC–HRMS/MS (method B) spectra of $[M + H]^+$ of [Atda⁵]MC-LR (**14**) and [D-Abu¹,Atda⁵]MC-LR (**15**), showing an expansion (*m*/*z* 65–305) from Figure S35. Ions connected with orange lines differ by 14.0157 Da (CH_2), and are attributable to product ions containing the amino acid at position-1.

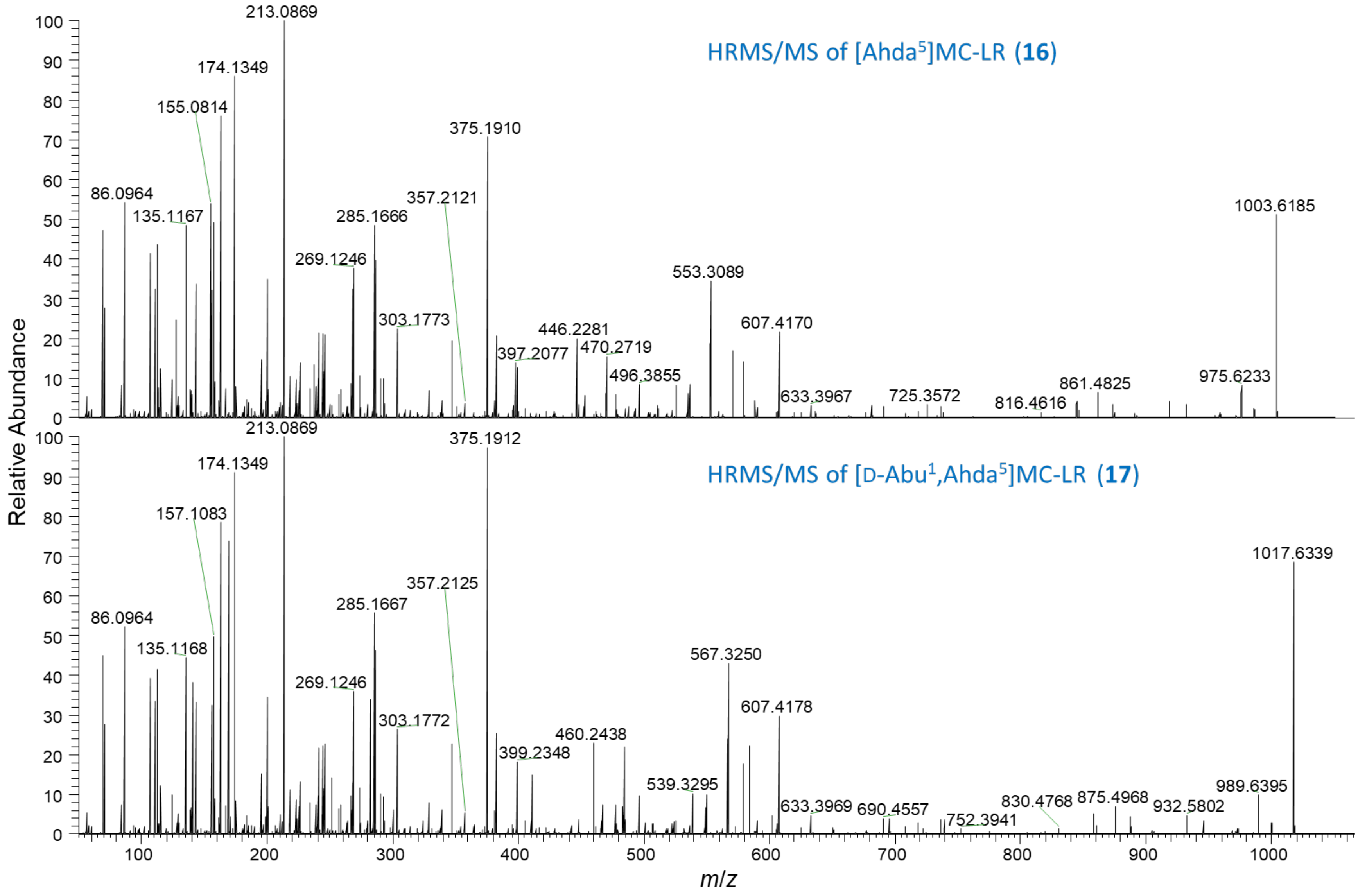

Figure S39. LC–HRMS/MS (method B) spectra of $[M + H]^+$ of [Ahda^5]MC-LR (**16**) and [D-Abu^1,Ahda^5]MC-LR (**17**).

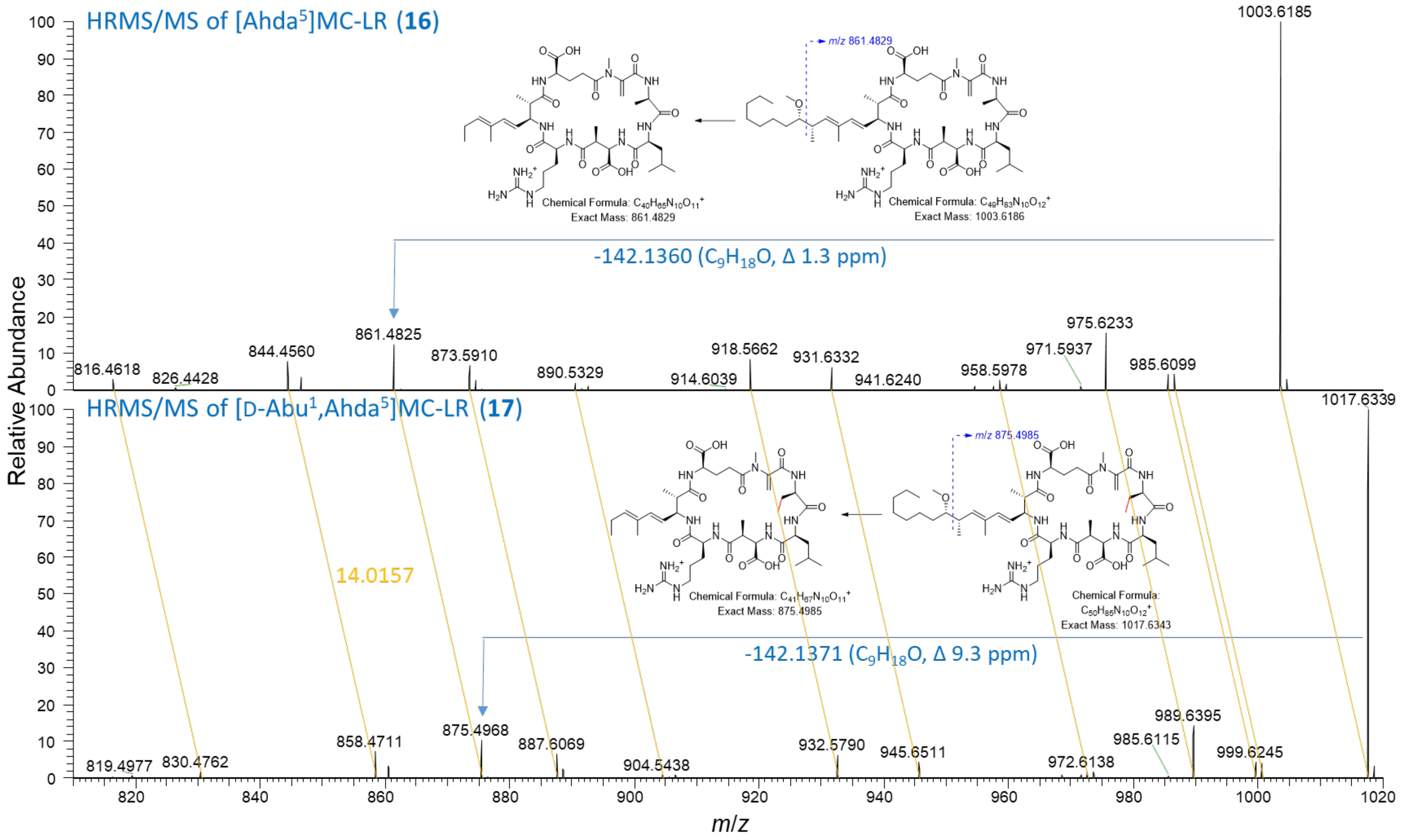

Figure S40. LC–HRMS/MS (method B) spectra of $[M + H]^+$ of [Ahda^5]MC-LR (**16**) and [D-Abu^1,Ahda^5]MC-LR (**17**), showing an expansion (*m*/*z* 810–1020) from Figure S39. Note the neutral loss of the Adda terminus (142.1358 Da). Ions connected with orange lines differ by 14.0157 Da (CH_2), and are attributable to product ions containing the amino acid at position-1.

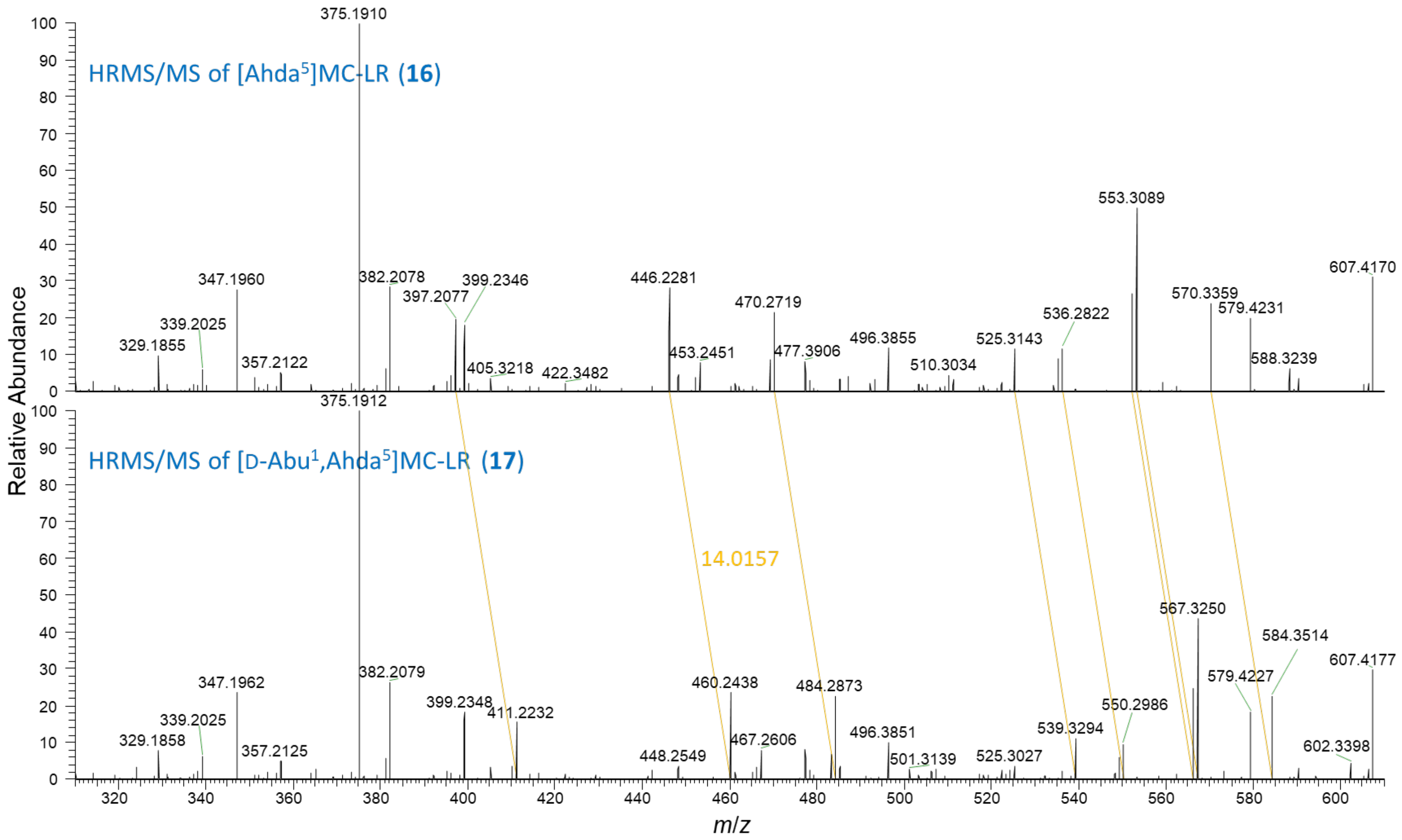

Figure S41. LC–HRMS/MS (method B) spectra of $[M + H]^+$ of [Ahda^5]MC-LR (**16**) and [D-Abu^1,Ahda^5]MC-LR (**17**), showing an expansion (*m*/*z* 310–610) from Figure S39. Ions connected with orange lines differ by 14.0157 Da (CH_2), and are attributable to product ions containing the amino acid at position-1.

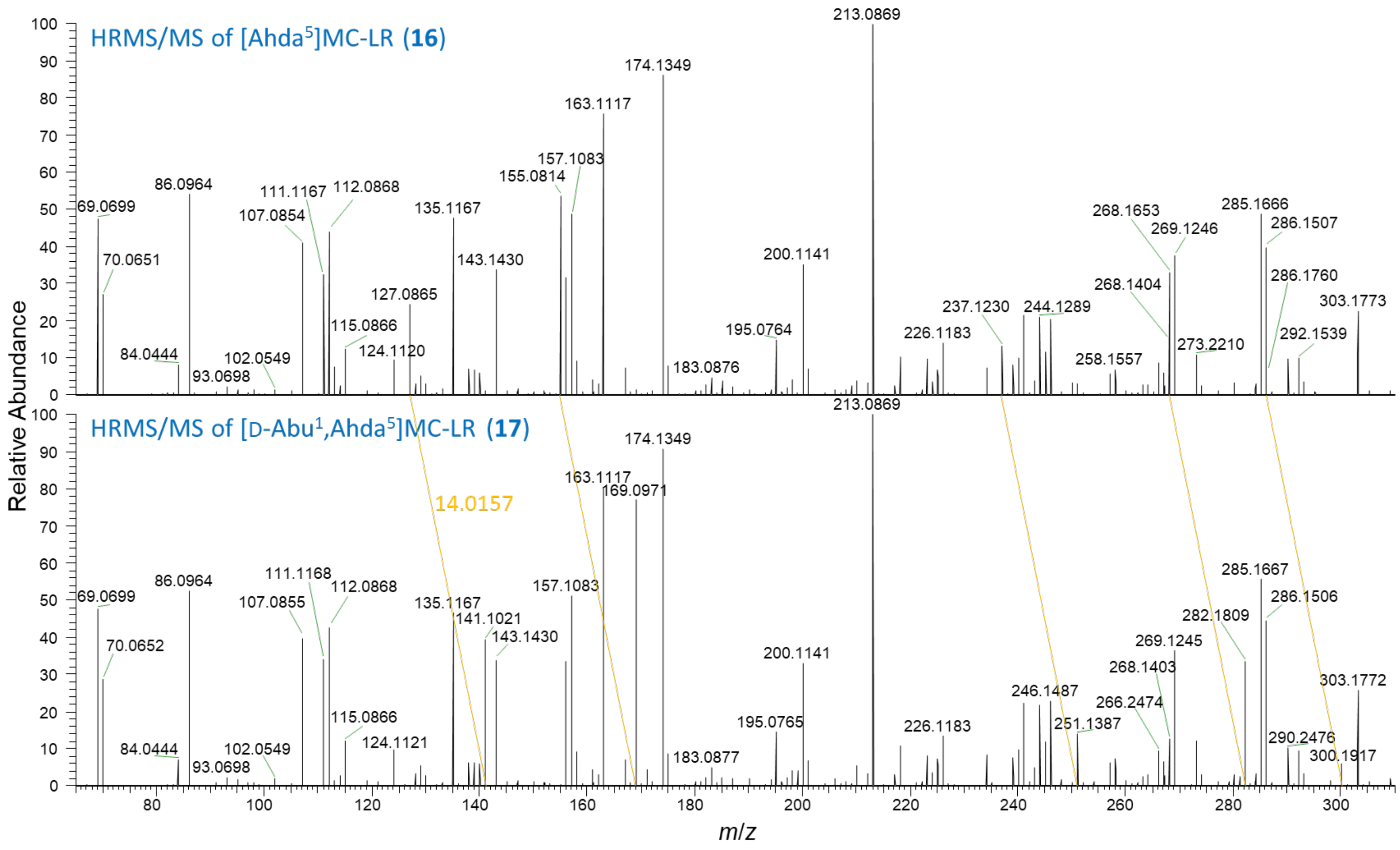

Figure S42. LC–HRMS/MS (method B) spectra of $[M + H]^+$ of [Ahda[5]]MC-LR (**16**) and [D-Abu[1],Ahda[5]]MC-LR (**17**), showing an expansion (*m/z* 75–310) from Figure S39. Ions connected with orange lines differ by 14.0157 Da (CH_2), and are attributable to product ions containing the amino acid at position-1.

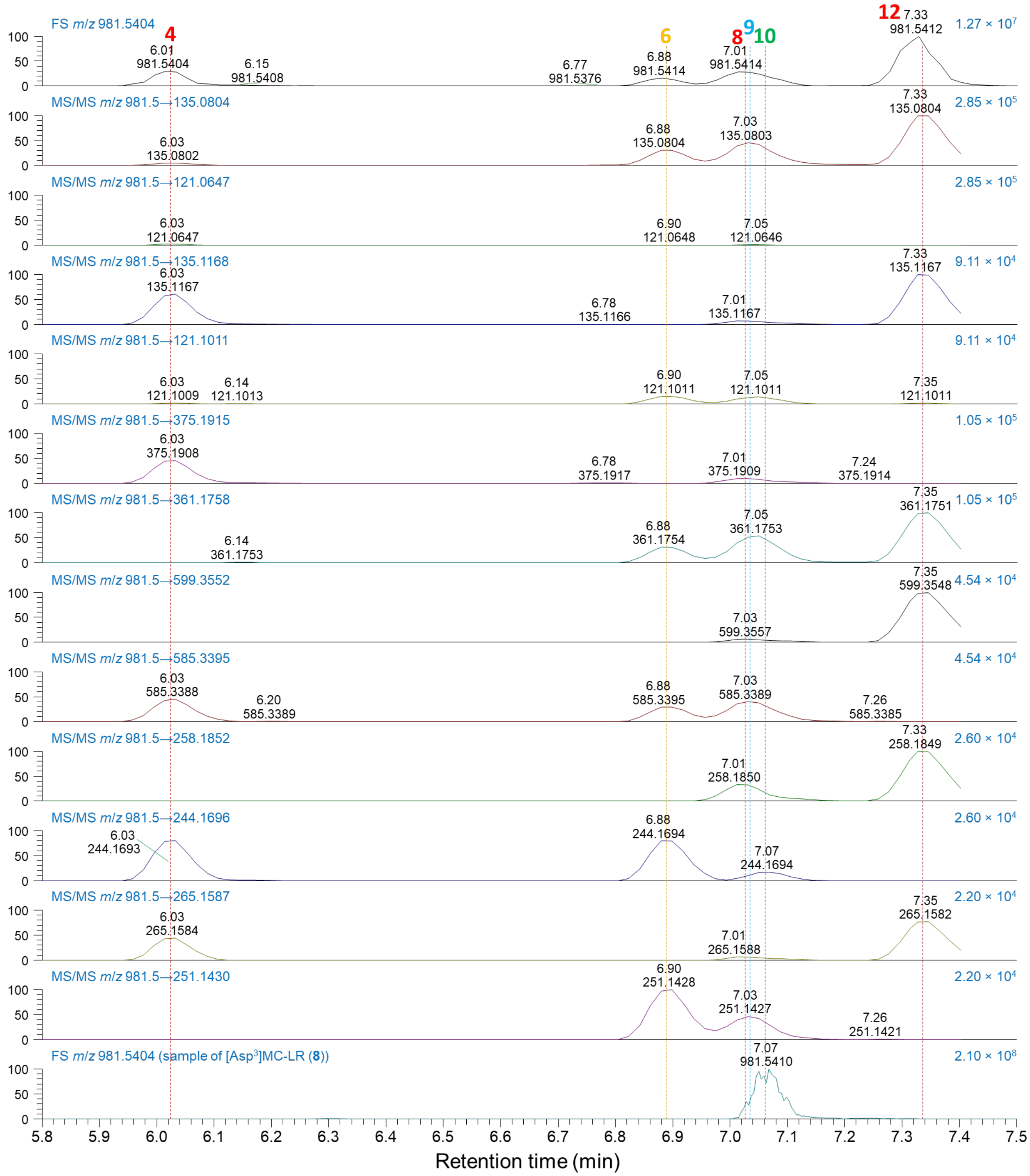

Figure S43. Extracted ion LC–HRMS/MS chromatograms of demethylated MC-LR precursor ion ($[M + H]^+$ at *m*/*z* 981.5), showing the presence of analogues **4**, **6**, **8**–**10**, and **12**. The product ion spectra are extracted at pairs of *m*/*z* values for demethylated and non-demethylated product ions (Table S2), and each resulting pair of chromatograms is shown with the same vertical scale to indicate relative abundances. The top chromatogram is from the full-scan of the extract at *m*/*z* 981.5404, the bottom is from a full-scan chromatogram of a standard of [D-Asp^3]MC-LR (**8**).

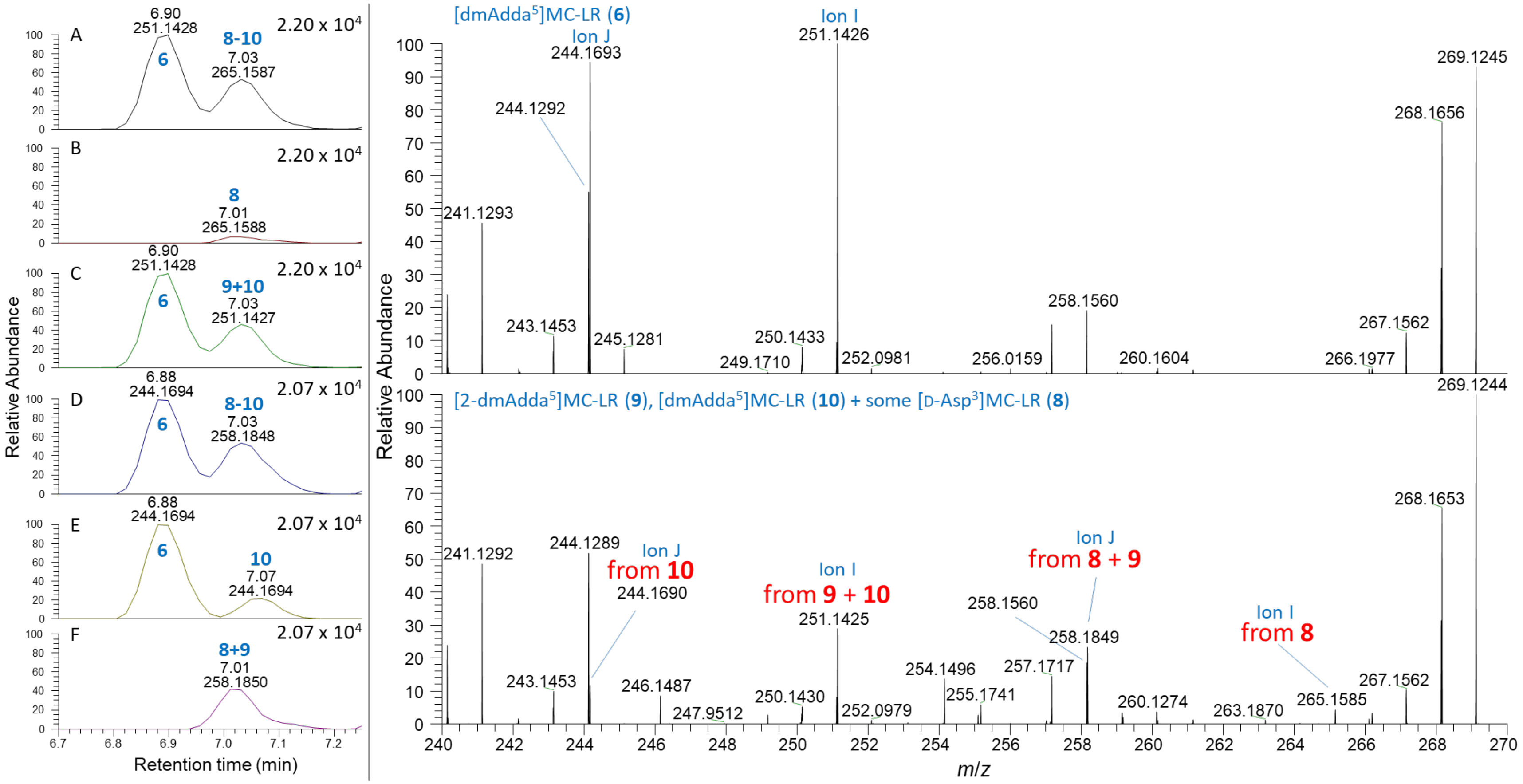

Figure S44. Left, LC–HRMS/MS chromatograms of precursors at $[M + H]^+$ *m/z* 981.5 extracted for ion I at: A, *m/z* 265.1587 + 251.1430; B, *m/z* 265.1587, and; C, *m/z* 251.1430; and for ion J at: D, *m/z* 258.1852 + 244.1696; E, *m/z* 244.1696, and; F, 258.1852. Right, expansion of LC–HRMS/MS spectra for the peaks at 6.88 ([6- or 8-dmAdda^5]MC-LR (**6**)) and 7.07 min ([Asp^3]MC-LR (**8**), [2-dmAdda^5]MC-LR (**9**), and [6- or 8-dmAdda^5]MC-LR (**10**)). Table S2 contains possible structures, and a list of expected *m/z* values, for product ions (positive ionization mode) of a range of Adda variants of MC-LR. See Figure S49 for a similar analysis, conducted retrospectively, of LC–HRMS/MS data obtained from a dietary supplement during a recent study.

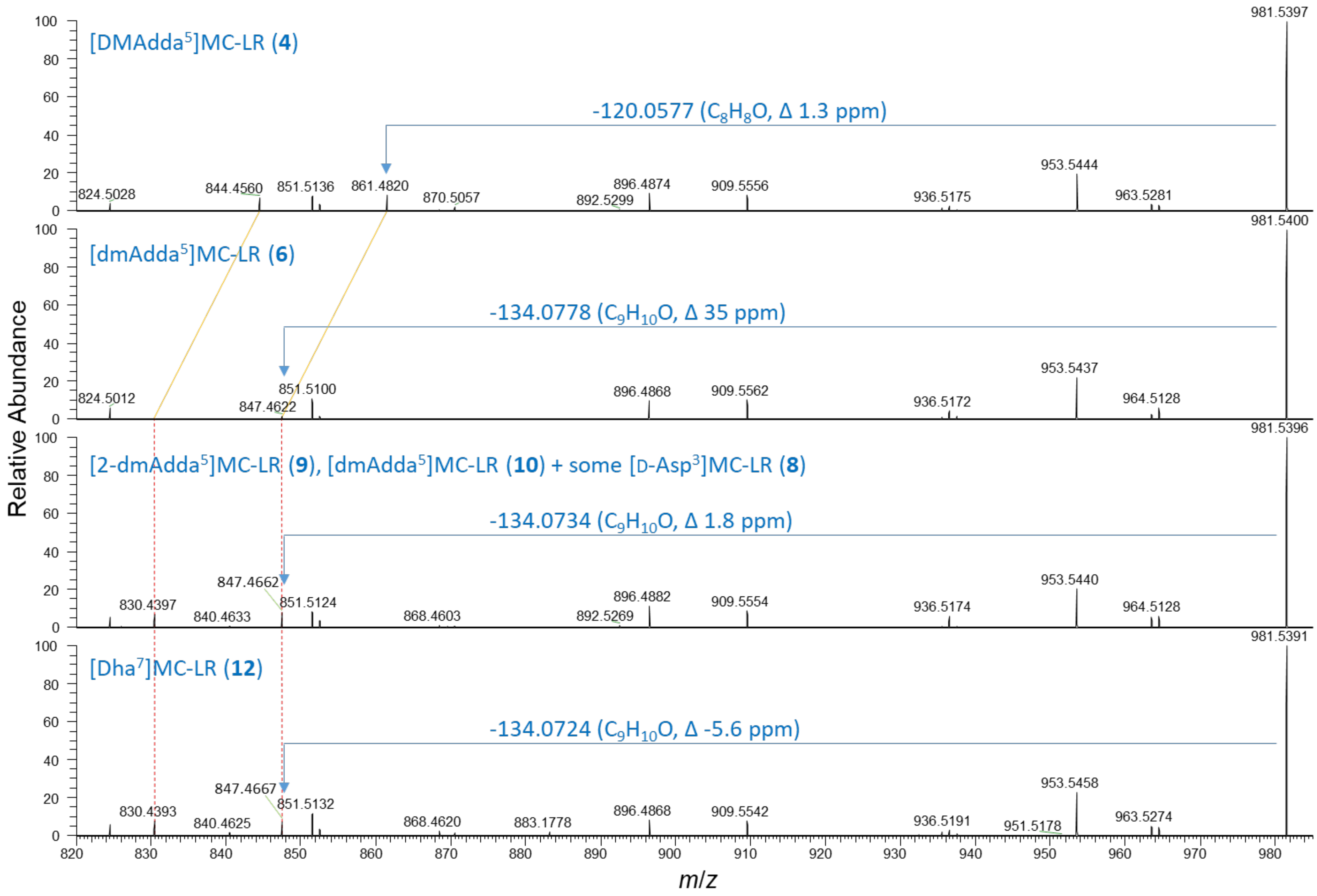

Figure S45. LC–HRMS/MS (method B) spectra of $[M + H]^+$ of demethylated MC-LR analogues **4**, **6**, **8**–**10** and **12**.

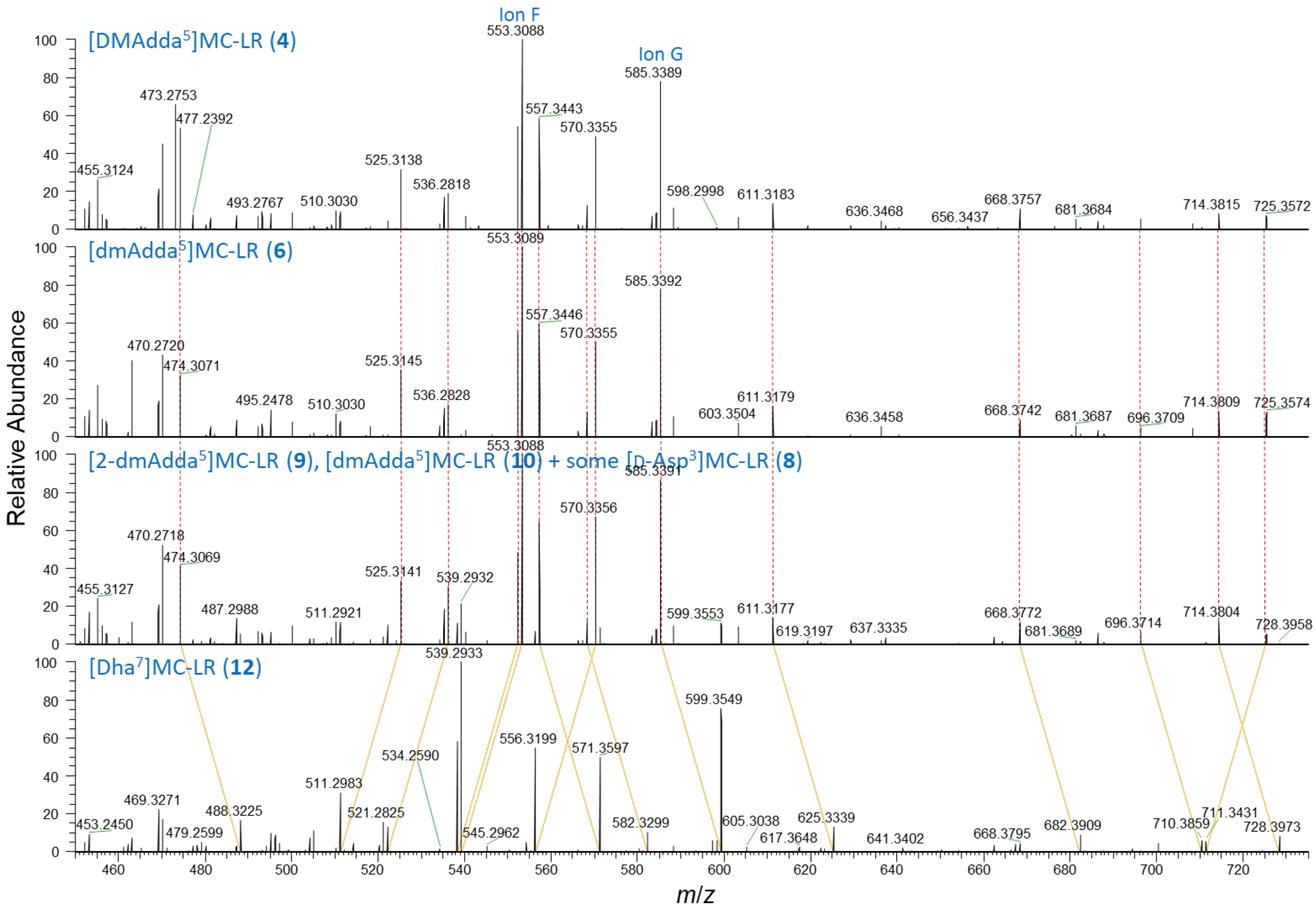

Figure S46. LC–HRMS/MS (method B) spectra of $[M + H]^+$ of demethylated MC-LR analogues **4**, **6**, **8**–**10** and **12**, showing an expansion (*m*/*z* 450–735) from Figure S41. Ions connected with orange lines differ by 14.0157 Da (CH_2).

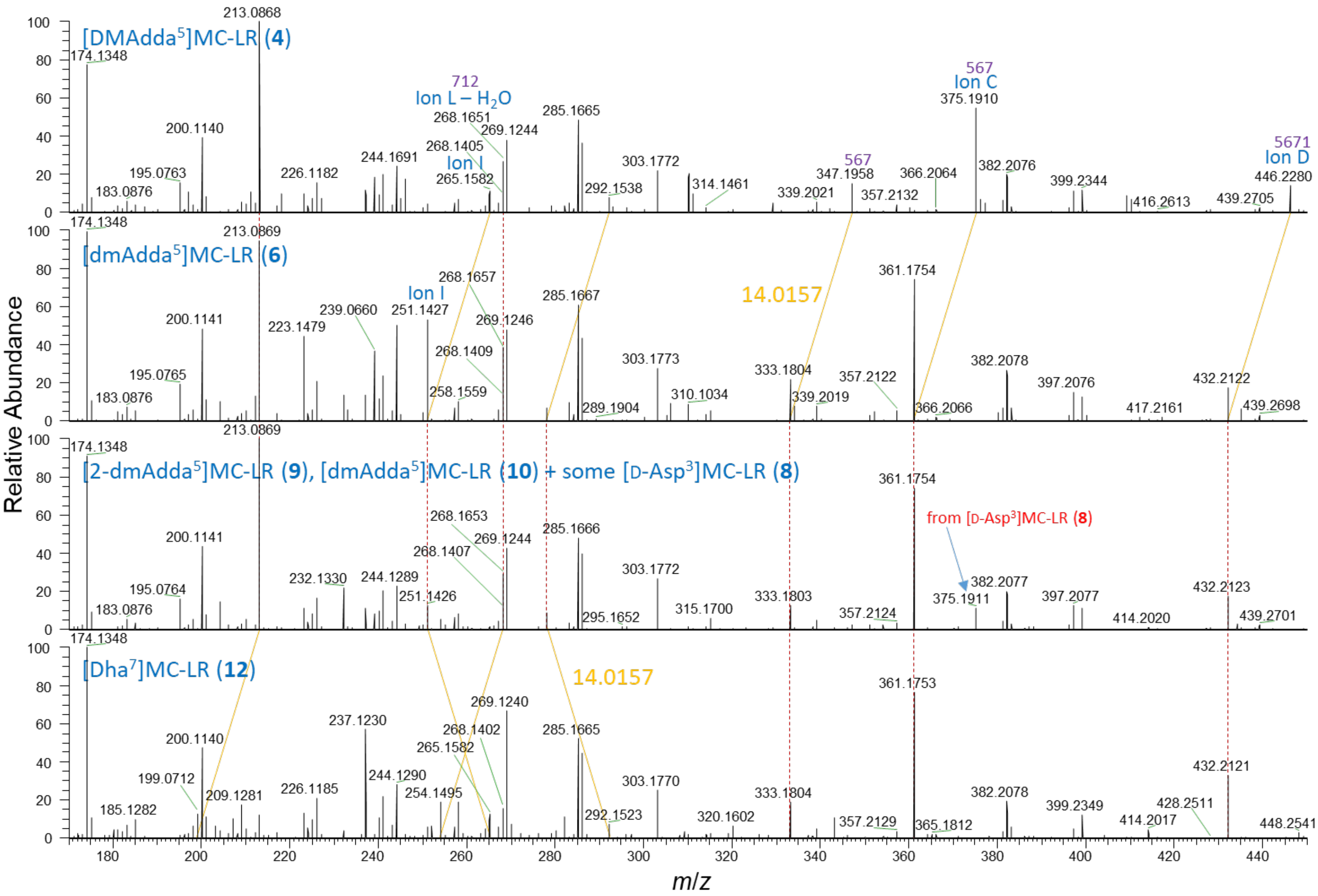

Figure S47. LC–HRMS/MS (method B) spectra of $[M + H]^+$ of demethylated MC-LR analogues **4**, **6**, **8**–**10** and **12**, showing an expansion (*m*/*z* 170–450) from Figure S41. Ions connected with orange lines differ by 14.0157 Da (CH_2).

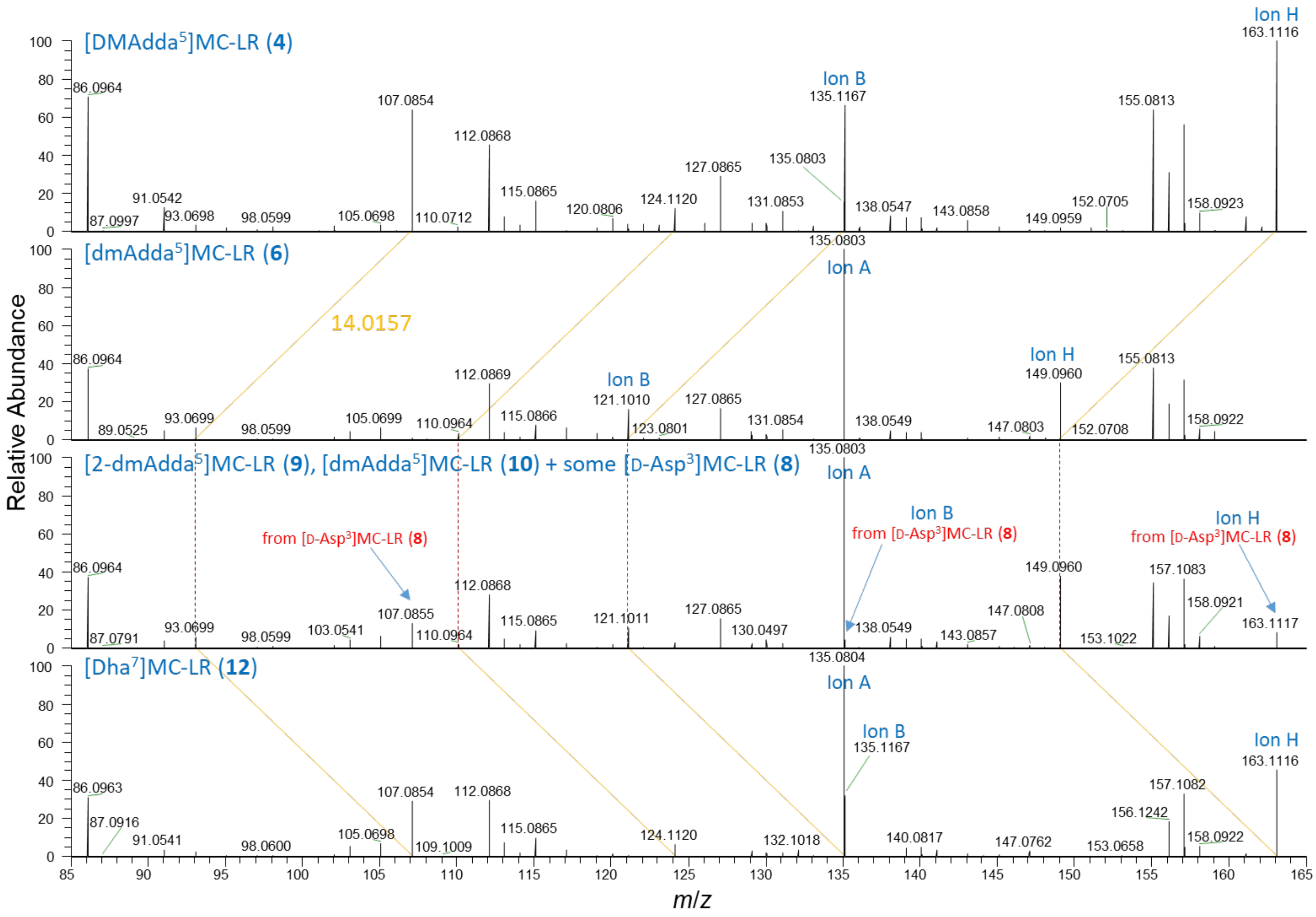

Figure S48. LC–HRMS/MS (method B) spectra of $[M + H]^+$ of demethylated MC-LR analogues **4**, **6**, **8**–**10** and **12**, showing an expansion (*m*/*z* 85–165) from Figure S41. Ions connected with orange lines differ by 14.0157 Da (CH_2).

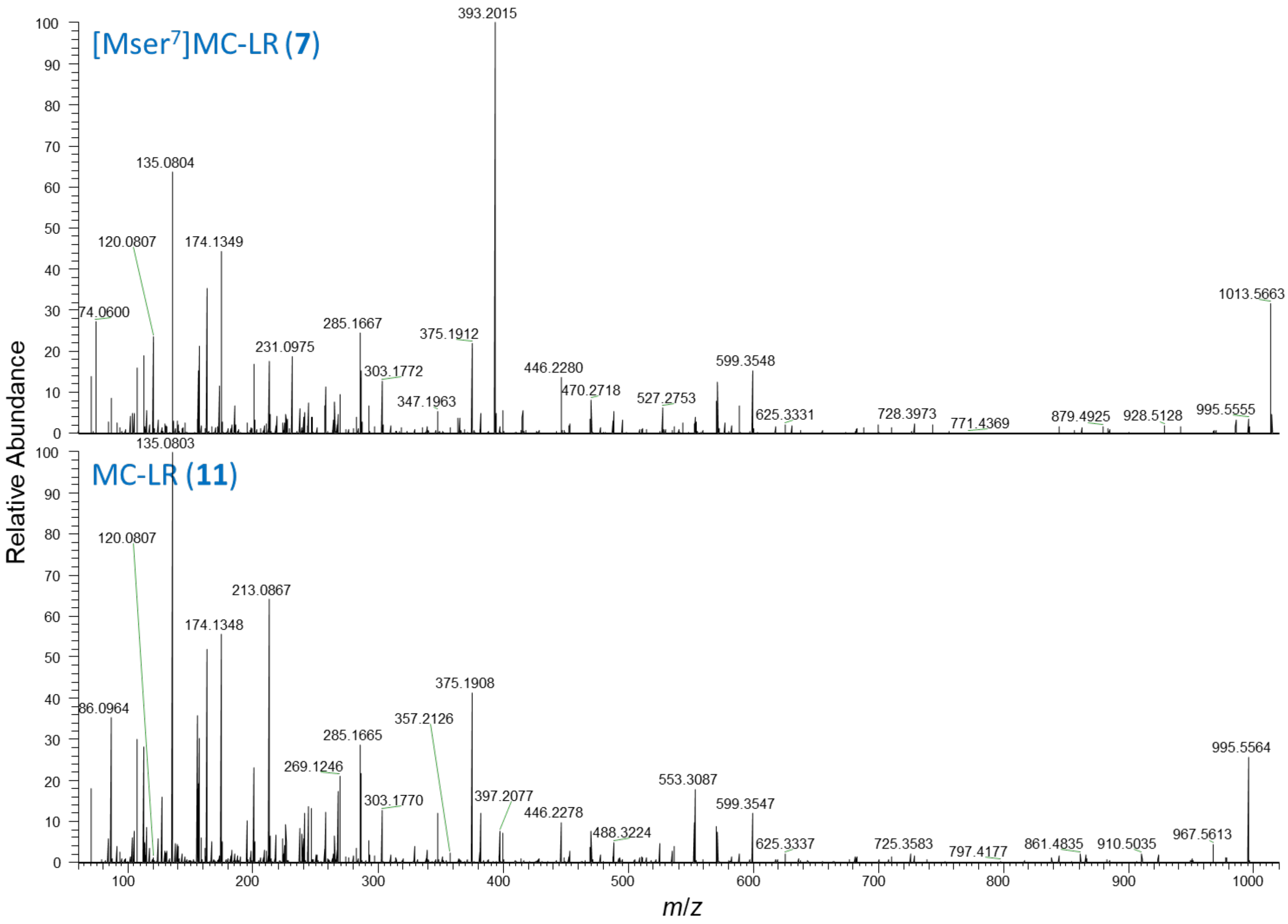

Figure S49. LC–HRMS/MS (method B) spectra of $[M + H]^+$ of: top, [Mser^7]MC-LR (**7**) at *m*/*z* 1013.5, and; bottom, MC-LR (**11**) at *m*/*z* 995.5, in an extract from the heterologous microcystin expression system.

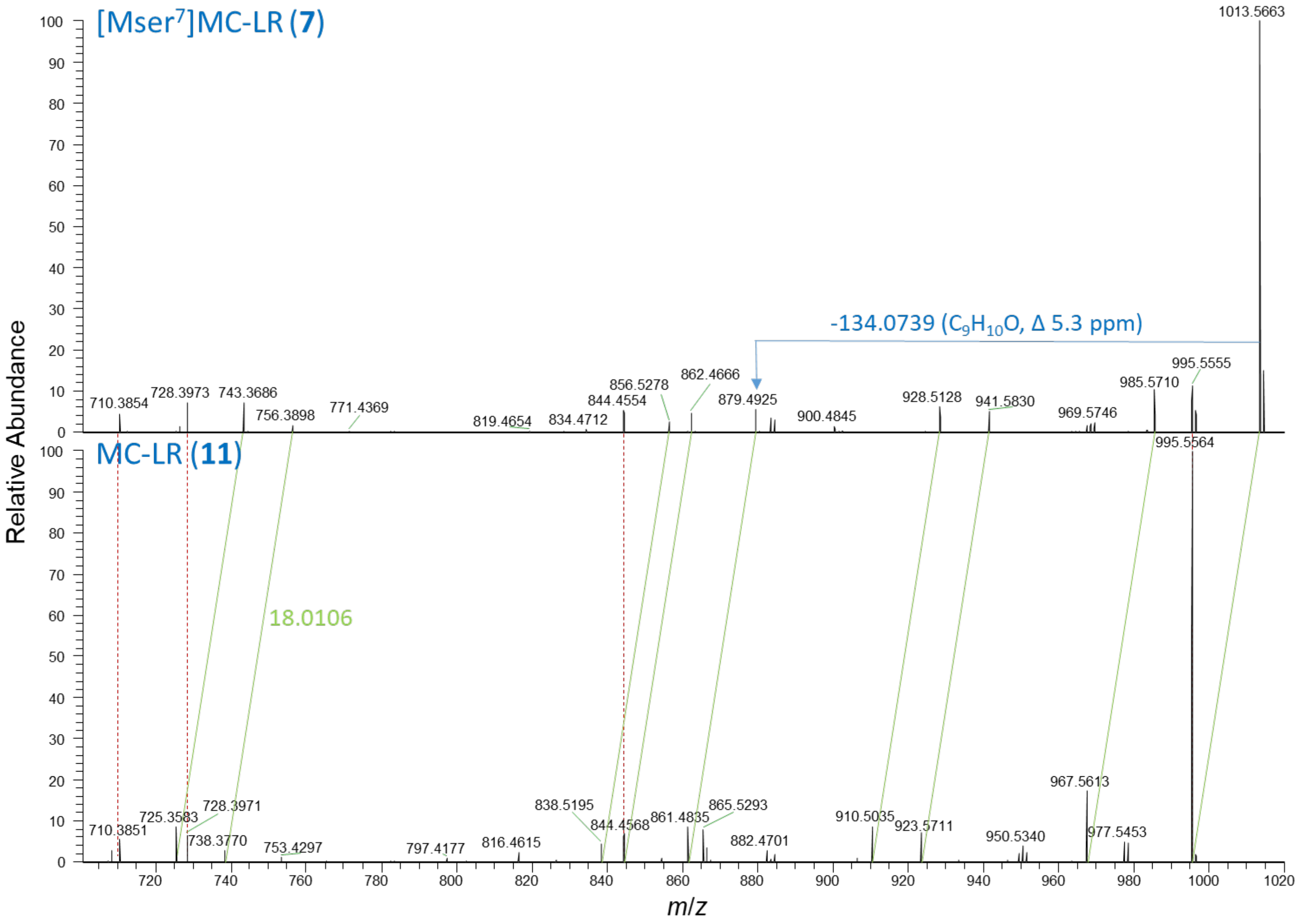

Figure S50. Expansion of the LC–HRMS/MS spectra (*m*/*z* 700–1020) from Figure S45, of $[M + H]^+$ of: top, [Mser^7]MC-LR (**7**) at *m*/*z* 1013.5, and; bottom, MC-LR (**11**) at *m*/*z* 995.5, in an extract from the heterologous microcystin expression system. Ions connected by green lines differ by 18.0106 Da (H_2O) and are attributable to product ions containing the amino acid at position-7.

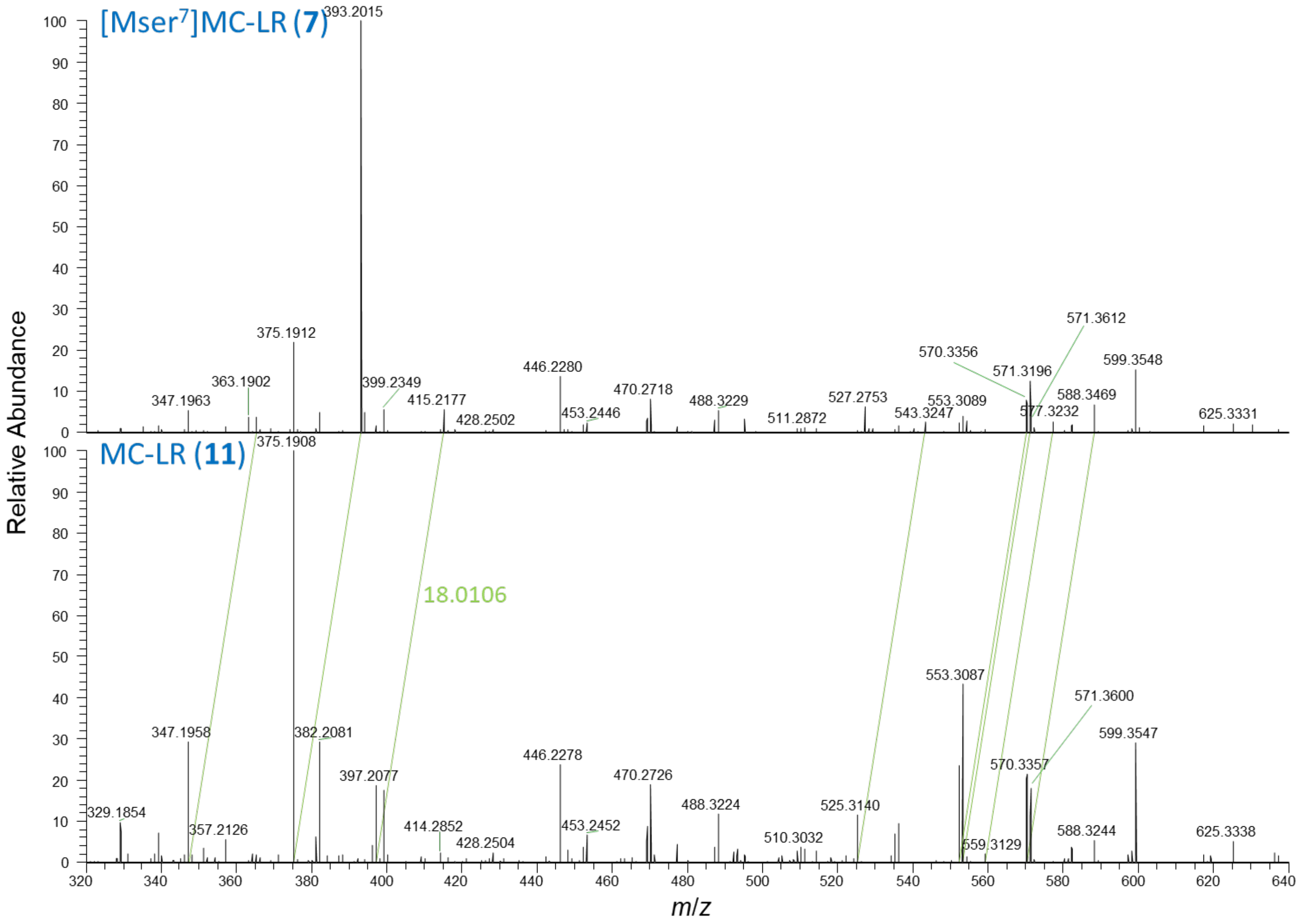

Figure S51. Expansion of the LC–HRMS/MS spectrum (*m*/*z* 320–640) from Figure S45, of $[M + H]^+$ of: top, [Mser^7]MC-LR (**7**) at *m*/*z* 1013.5, and; bottom, MC-LR (**11**) at *m*/*z* 995.5, in an extract from the heterologous microcystin expression system. Ions connected by green lines differ by 18.0106 Da (H_2O) and are attributable to product ions containing the amino acid at position-7.

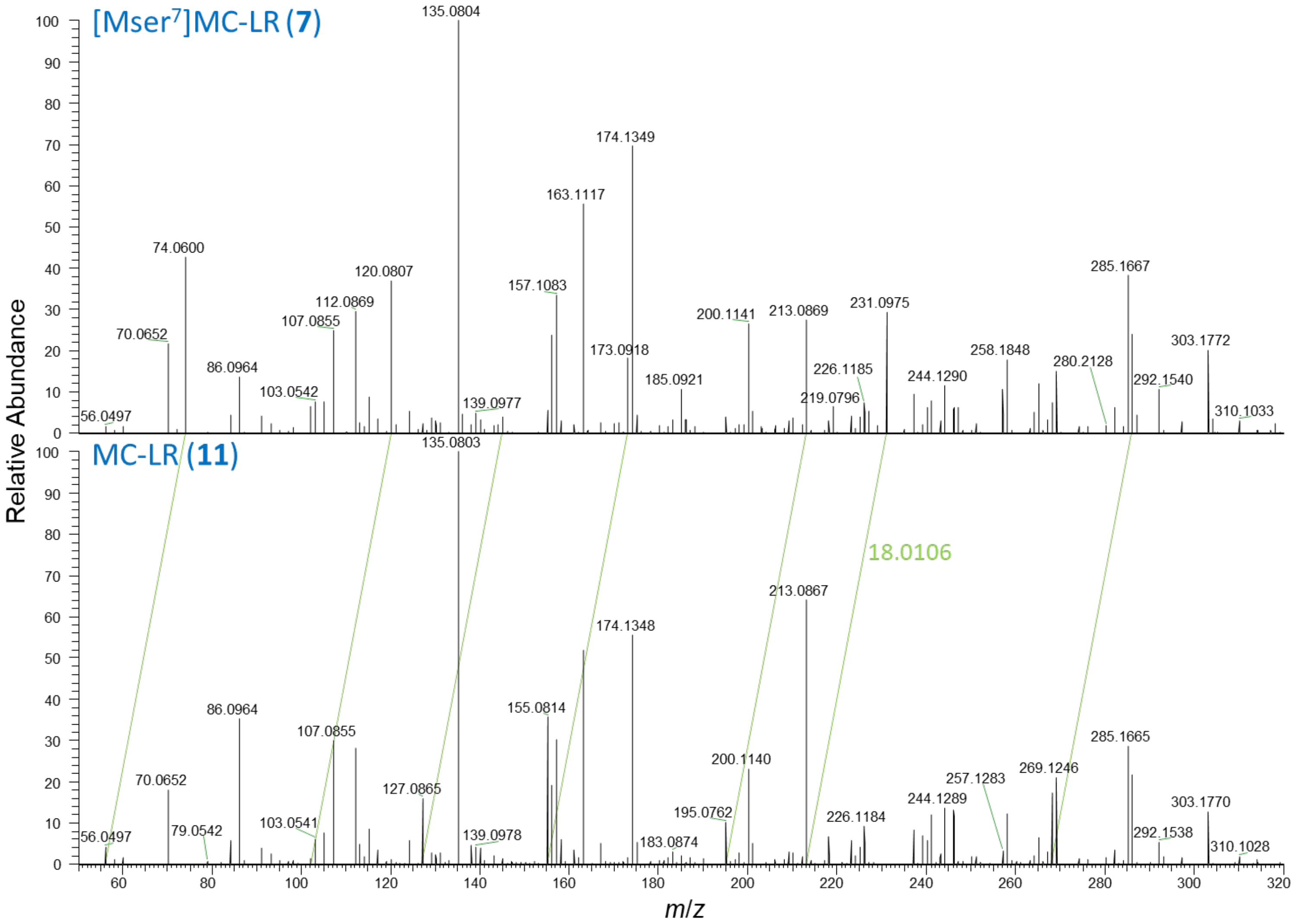

Figure S52. Expansion of the LC–HRMS/MS spectrum (*m*/*z* 50–320) from Figure S45, of $[M + H]^+$ of: top, [Mser^7]MC-LR (**7**) at *m*/*z* 1013.5, and; bottom, MC-LR (**11**) at *m*/*z* 995.5, in an extract from the heterologous microcystin expression system. Ions connected by green lines differ by 18.0106 Da (H_2O) and are attributable to product ions containing the amino acid at position-7.

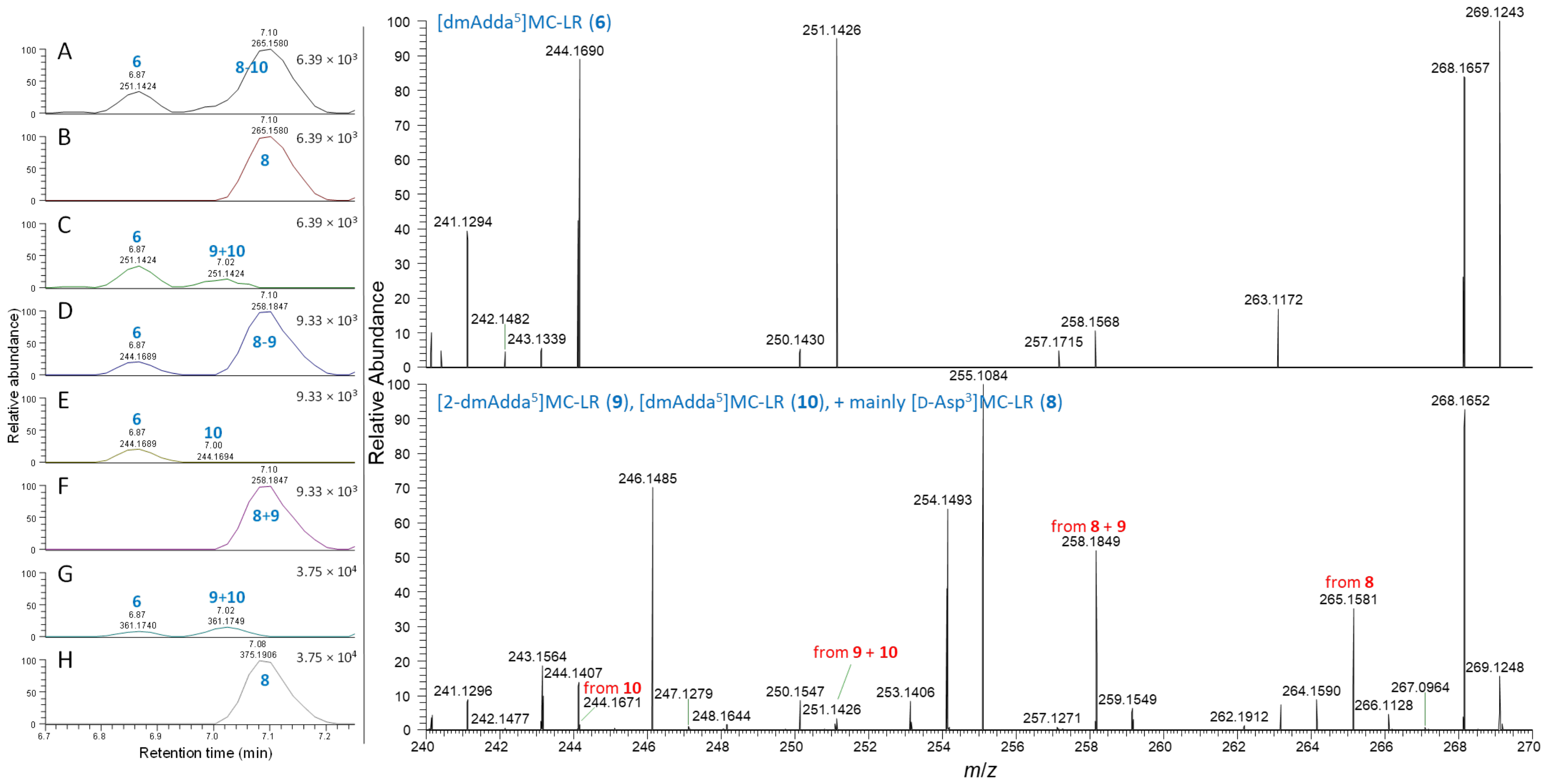

Figure S53. Retrospective analysis of LC–HRMS/MS data for demethylated MC-LR congeners obtained during a study of dietary supplements.* Left, LC–HRMS/MS chromatograms of precursors at $[M + H]^+$ *m/z* 981.5 extracted for ion I at: A, *m/z* 265.1587 + 251.1430; B, *m/z* 265.1587, and; C, *m/z* 251.1430; and for ion J at: D, *m/z* 258.1852 + 244.1696; E, *m/z* 244.1696; F, 258.1852; G, *m/z* 361.1758, and; *m/z* 375.1914. Right, expansion of LC–HRMS/MS spectra for the peaks at 6.87 ([6- or 8-dmAdda^5]MC-LR (**6**)) and 7.10 min ([Asp^3]MC-LR (**8**), [2-dmAdda^5]MC-LR (**9**), and [6- or 8-dmAdda^5]MC-LR (**10**)). See Figure S40 for corresponding data from the present sample.

*Miller, T. R.; Xiong, A.; Deeds, J. R.; Stutts, W. L.; Samdal, I. A.; Løvberg, K. E.; Miles, C. O., Microcystin toxins at potentially hazardous levels in algal dietary supplements revealed by a combination of bioassay, immunoassay, and mass spectrometric methods. *J. Agric. Food Chem*. **2020**, 68, 8016–8025.

Table S2. Selected exact masses of characteristic product ions (observed or expected) involving cleavages related to the Adda^{5}-moiety of microcystins in positive ionization mode. Values in black are the same as for MC-LR (**11**), while those in red are for the specified demethylated analogue and those in blue are for analogues with a modified Adda terminus[a]

Microcystin		Exact *m*/*z* (positive) of diagnostic fragments involving Adda						
		Ion A	Ion B	Ion H	Ion C	Ion G	Ion J	Ion I
MC-LR	**11**	135.0804	135.1168	163.1117	375.1914	599.3552	258.1852	265.1587
[DMAdda^{5}]MC-LR[b]	**4**	121.0647[c]	135.1168	163.1117	375.1914	585.3395	244.1696	265.1587
[8-dmAdda^{5}]MC-LR	**6/10**	135.0804	121.1011	149.0961	361.1758	585.3395	244.1696	251.1430
[6-dmAdda^{5}]MC-LR	**6/10**	135.0804	121.1011	149.0961	361.1758	585.3395	244.1696	251.1430
[2-dmAdda^{5}]MC-LR	**9**	135.0804	121.1011	149.0961	361.1758	585.3395	258.1852	251.1430
[D-Asp^{3}]MC-LR	**8**	135.0804	135.1168	163.1117	375.1914	599.3552	258.1852	265.1587
[Dha^{7}]MC-LR	**12**	135.0804	135.1168	163.1117	361.1758	599.3552	258.1852	265.1587
[Atda^{5}]MC-LR	**14**	115.1117	135.1168	163.1117	375.1914	579.3865	238.2165	245.1900
[Ahda^{5}]MC-LR	**16**	143.1430	135.1168	163.1117	375.1914	607.4178	266.2478	273.2213

[a]Possible structures for selected product ions involving Adda^{5} are shown below. The exact masses match the observed accurate masses, but locations of unsaturation or the charge may vary. The numbers in blue indicate atom numbering on the Adda side chain, and methyl groups marked in red. [b]I.e. [9-*O*-desmethylAdda^{5}]MC-LR. [c]Only observed at very low intensity.

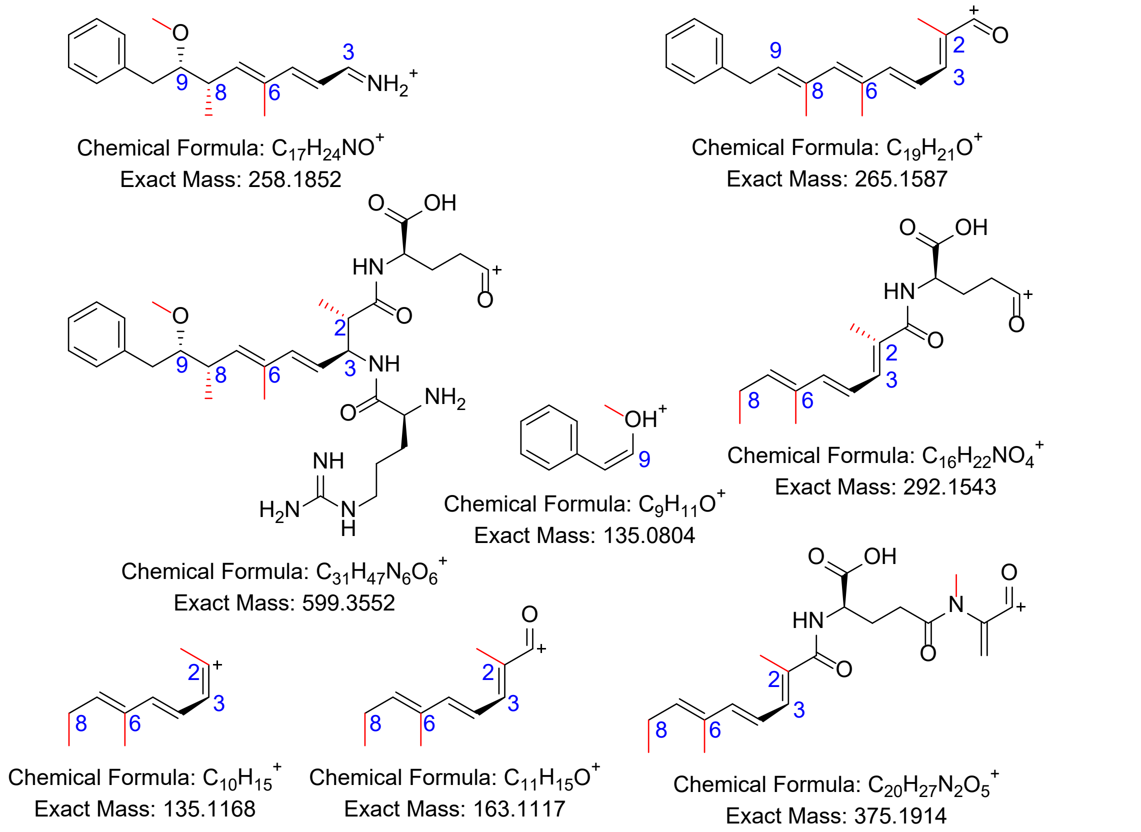

Supplement: Supplementary file 1 — ao4c03332_si_001.pdf [file ao4c03332_si_001.pdf]
